# Oxygen-Unaffected Temperature Sensing in Air-Saturated Aqueous Dispersion with Oxygen-Sensitive Luminescent Metal Complexes Encapsulated in l‑Arginine-Type Silica Networks

**DOI:** 10.1021/acsmeasuresciau.6c00124

**Published:** 2026-08-10

**Authors:** Viktoriia Osipova, Christian Homann, Arne C. Weber, Lingcong Ge, Isabella Tavernaro, Elena Meßmer, Katja Heinze, Michael R. Reithofer, Christoph Kerzig, Ute Resch-Genger

**Affiliations:** † Federal Institute for Materials Research and Testing (BAM), Division 1.2 Biophotonics, Richard-Willstaetter-Str. 11, Berlin 12489, Germany; ‡ Free University Berlin, Institute for Chemistry and Biochemistry, Takustr. 3, Berlin 14195, Germany; § Department of Chemistry, 9182 Johannes Gutenberg University Mainz, Duesbergweg 10-14, Mainz 55128, Germany; ∥ Institute of Inorganic Chemistry, Faculty of Chemistry, 27258 University of Vienna, Waehringer Str. 42, Vienna 1090, Austria

**Keywords:** photoactive chromium(III) complexa, photoactive ruthenium(II) complex, luminescence, optical spectroscopy, oxygen shielding, silica nanoparticles, temperature sensing

## Abstract

Bioimaging, sensing, and photonic applications of oxygen-sensitive luminophores such as near-infrared (NIR) emissive spin-flip complexes of earth-abundant chromium (Cr^III^) and triplet metal-to-ligand charge transfer complexes like ruthenium (Ru^II^), osmium (Os^II^) or iridium (Ir^III^) compounds in air require efficient strategies for their complete protection from luminescence quenching by oxygen (O_2_). Here, we explored the applicability of our l-arginine-type sol–gel nanoparticle synthesis route developed for complete oxygen shielding of a molecular ruby-type luminophore [Cr­(ddpd)_2_]­(BF_4_)_3_ (**Crddpd**) to a representatively chosen Ru^II^ complex [Ru­(phen)_3_]­Cl_2_ (**Ruphen**) and a Ru^II^ pyrene dyad [Ru­(phen)_2_(phenpy)]­Cl_2_ (**Ruphenpy**) with luminescence lifetimes of 1.1 μs and ∼50 μs in the absence of O_2_, thus confirming its versatility. Temperature-dependent steady-state and time-resolved luminescence measurements with the Ru^II^ and Cr^III^ complexes in air- and argon (Ar)-saturated aqueous solution and encapsulated in silica nanoparticles (SiO_2_ NPs) prepared by the l-arginine and the classical Stöber method demonstrated the preserved temperature sensitivity of these luminophores in both silica networks. These measurements highlight the importance of complete O_2_ protection for reliable temperature sensing with O_2_-sensitive luminophores in air. These findings, together with the enhanced photostability observed for l-arginine SiO_2_ NPs-encapsulated **Ruphen** and **Ruphenpy**, can pave the road for many applications of O_2_-sensitive systems in air in the life and material sciences.

## Graphical abstract

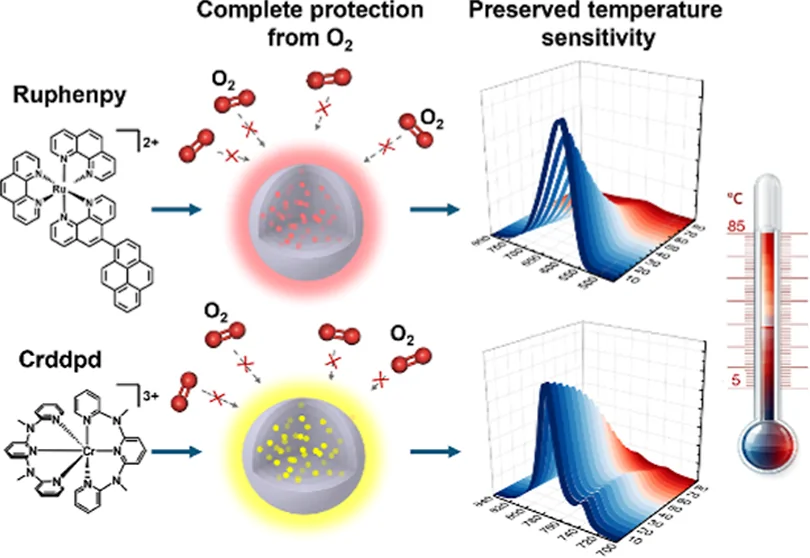

## 1 Introduction

Luminescent complexes of transition metal ions such as Ru^II^, Ir^III^, Os^II^, and Re^I^ with emission in the visible and near-infrared (NIR) regions, controlled by sophisticated ligand design, are frequently used as luminescent probes and reporters in sensing, bioimaging, materials science, and photonic applications.^1–10^ Particularly, Ru^II^ compounds have been widely utilized as building blocks in sensors for contactless and minimally invasive measurements of environmental parameters such as pressure and oxygen (O_2_), as well as temperature in the life and materials sciences and aerospace.^11–15^ These applications exploit the responsivity of their photoluminescence (PL) quantum yields (PLQY), their PL intensities, and their relatively long PL lifetimes in the range of typically several hundred nanoseconds to changes in these parameters.^16–18^ For applications such as time-gated luminescence, triplet–triplet annihilation upconversion (TTA-UC), and sensitizing solar cells as well as the determination of other environmental parameters or analytes, a pronounced O_2_ sensitivity of PLQY, PL intensity, and PL decay kinetics or lifetime can present a disadvantage. This calls for concepts for the simultaneous determination of oxygen and temperature. Typical approaches are the combination of combining luminophores varying in O_2_ and temperature sensitivity such as an O_2_- sensitive Ru^II^ complex and a quantum dot^19–24^ or efficient O_2_ shielding.^16,25–27^ For accurate and reliable sensor performance, commonly, complete O_2_ protection is preferred to avoid signal-distorting interferences.^12^ Concepts for O_2_ protection rely on preventing physical contact between the excited luminophore and O_2_, which is required for Dexter-type energy transfer. This includes the synthetically challenging usage of bulky ligands or counterions, which, however, only partly hinders O_2_ diffusion to the spin-flip emissive chromium center^28^ and is difficult to employ with charge-transfer emitters due to their excited state function extending over large parts of the ligand framework. On the other hand, luminophores can be encapsulated in matrices with a very low O_2_ permeability, such as polyacrylonitrile, poly-α-pinene, or poly­(vinyl alcohol).^29–34^ Alternatively, biocompatible silica nanomaterials and films have been utilized for O_2_ protection. These silica materials were typically prepared by the well-established Stöber route.^35–38^ For most matrix-embedded O_2_-sensitive luminophores, the O_2_ permeability and shielding efficiency of the matrix has not been quantified despite its impact on sensor performance.^12,39^ This allows O_2_ interference with the measurement of other parameters or analytes and can hamper comparability across different sensors, i.e., luminophores and matrices.

Complete O_2_ protection of two O_2_-sensitive luminescent complexes of earth-abundant Cr^III^ with lifetimes in the order of 1 ms has been recently achieved by us with amorphous nonporous silica nanoparticles (SiO_2_ NPs) prepared by l-arginine mediated silica formation.^25^ In the present study, we explore the versatility and general applicability of our l-arginine silica-oxygen shielding approach for transition metal complexes such as a common Ru^II^ complex, here [Ru­(phen)_3_]^2+^ (**Ruphen**), and the metal complex-organic chromophore dyad (**Ruphenpy**). The latter consists of a [Ru­(phen)_3_]^2+^ complex covalently linked to a pyrene moiety (Figure 1).^40^ This dyad was chosen because of its unusually long PL lifetime of about 50 μs in the absence of O_2,_ which is exceptionally long for a Ru^II^ complex^40–42^ but can be explained by the intermediate population of the long-lived organic triplet state (Figure 1, middle). Further, we assessed the temperature sensitivity of the PL properties of both SiO_2_ NP-encapsulated Ru^II^ compounds and the temperature-responsive Cr^III^ complex [Cr­(ddpd)_2_]­(BF_4_)_3_ (**Crddpd**). The latter has previously been employed as a self-referenced ratiometric optical Boltzmann thermometer in the temperature range of 5 to 85 °C in air- and Ar-saturated solution.^43^ We now compared the results with those obtained for the corresponding molecular systems. The present metal complexes differ in the nature of the respective excited state (spin-flip, ^3^MLCT, ^3^π–π*), O_2_ PL quenching dynamics, and temperature sensing mechanism. The two long-lived lowest-energy spin-flip states of the Cr^III^ complex (see middle right panel of Figure 1), which allow ratiometric temperature sensing, are more shielded from the environment due to the metal-centered spin-flip nature of the excited state. These Cr^III^ complexes show O_2_ PL quenching rate constants orders of magnitude below the diffusion limit.^28^ In contrast, the lowest excited triplet states of the Ru-based compounds, i.e., the ^3^MLCT state for **Ruphen** and a ^3^π–π* state for **Ruphenpy**, are quenched by O_2_ at rate constants close to the diffusion limit, with most collisions with dissolved O_2_ depopulating the excited states.^40,44–46^ As the PL intensity and PL lifetime or decay kinetics are also the parameters exploited for temperature sensing, the presence of O_2_ can be detrimental for reliable optical temperature measurements for luminophores such as the ruthenium chromophores, which is frequently underestimated.

**Figure 1:**
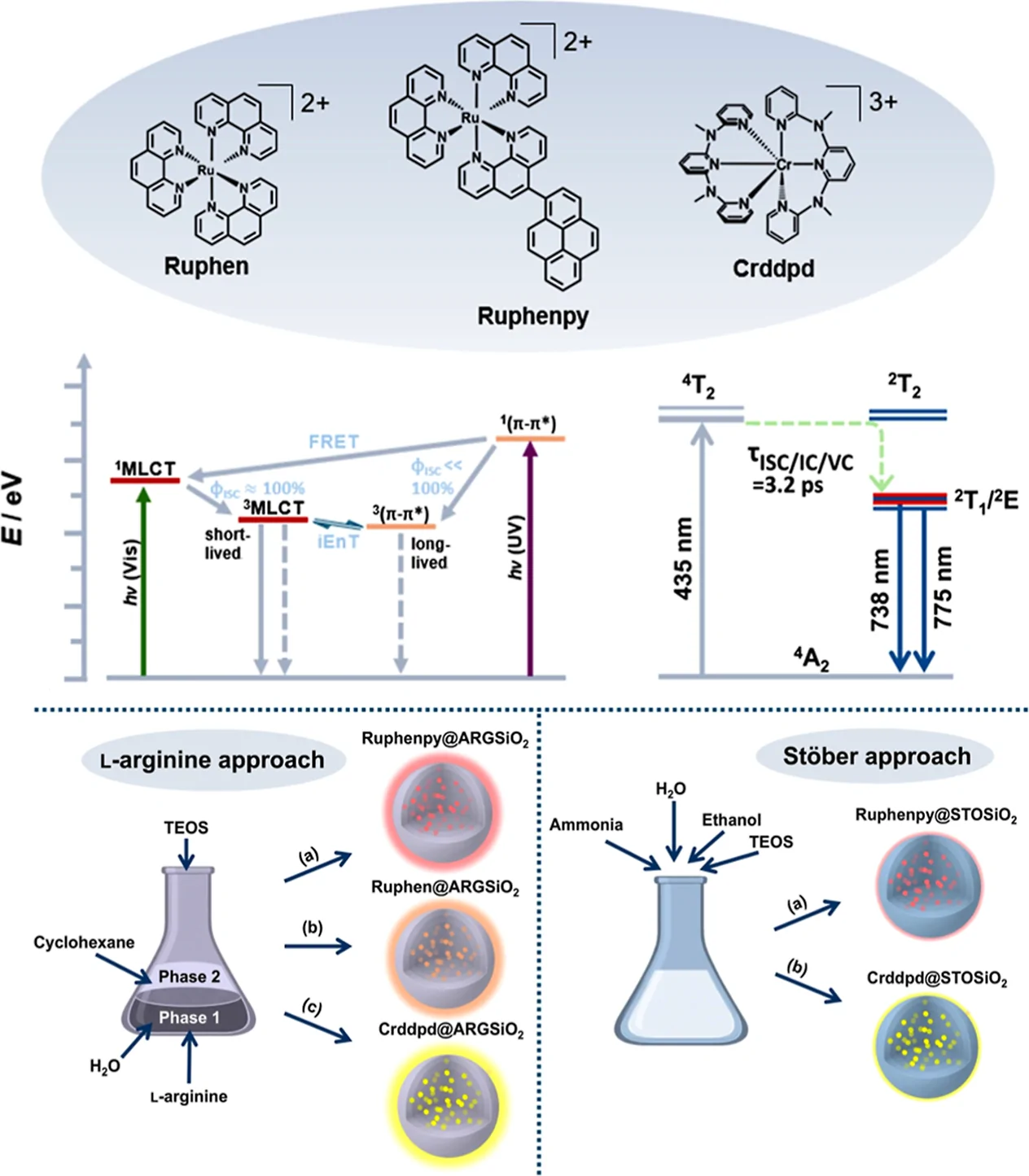
Overview of the luminophores, luminescence mechanism photophysics, and silica-encapsulation routes. Top: molecular structures of the Ru^II^ complexes **Ruphen** and **Ruphenpy** and the Cr^III^ complex **Crddpd**. Middle: simplified energy level scheme of **Ruphen** and **Ruphenpy** showing the photoexcitation of the Ru^II^ complex, intersystem crossing (ISC), and relaxation pathways (^1^MLCT → ^3^MLCT/^3^(π–π*) states), along with subsequent intramolecular energy-transfer processes (FRET/iEnT), with the respective energies as well as the simplified energy level diagram of the Cr^III^ spin-allowed excitation (^4^A_2_ → ^4^T_2_; 435 nm = 2.85 eV), IC (internal conversion), VR (vibrational relaxation), ISC and NIR emission from the ^2^T_1_/^2^E qmanifold (738 nm = 1.68 eV and 775 nm = 1.60 eV). Bottom: schematic presentation of the encapsulation of (a) **Ruphenpy**, (b) **Ruphen**, and (c) **Crddpd**, in silica nanoparticles (SiO_2_ NPs) prepared by two sol–gel routes. Left: l-arginine-catalyzed approach in a biphasic system (l-arginine, 0.52 mmol; H_2_O/cyclohexane = 15.3:1; TEOS, 24.63 mmol; 65 °C, 20 h) and right: Stöber process (TEOS, 0.55 mL; NH_3_(aq) 25%, 0.5 mL; EtOH/H_2_O = 57.5:1; r.t. (*T* = 23 ± 2 °C), 2 d, yielding **Ruphenpy@ARGSiO**_**2**_, **Ruphen@ARGSiO**_**2**_, **Crddpd@ARGSiO**_**2**_, **Ruphenpy@STOSiO**_**2**_, and **Crddpd@STOSiO**_**2**_.

The aim of our spectroscopic studies is to confirm the general applicability of our l-arginine silica shielding concept for O_2_ photosensitive metal complexes. This can pave the route to many new applications in air previously hampered by O_2_ induced PL quenching. In addition, we reveal on highlighting the influence of O_2_ on temperature sensing with O_2_-sensitive ratiometric luminophores to underline the importance of quantifying the O_2_ shielding sensitivity of matrices used for the design of sensor systems with O_2_-responsive luminophores. This presents an important prerequisite for accurate and comparable sensor performance and needs to be accordingly considered.

## 2 Experimental Section

### 2.1 Materials

TEOS (≥99%), acetonitrile (ACN, 99.9%), cyclohexane (99.5%), and **Ruphen** ([Ru­(phen)_3_]­Cl_2_, 98%) were purchased from Sigma-Aldrich (Germany), while ethanol (EtOH, absolute, 99.9%) was purchased from Labsolute (Th. Geyer, Germany), l-arginine (≥98.5%) from Carl Roth GmbH (Germany), and ammonia (25%) from abcr GmbH (Germany). All chemicals, reagents, and solvents were used as received, unless otherwise stated. All aqueous solutions utilized for the syntheses of the SiO_2_ NPs were prepared with Milli-Q-water (0.055 μS m^–1^; Merck Milli-Q IQ 700). All materials required for the synthesis of the molecular O_2_-sensitive luminophores **Ruphenpy** ([Ru­(phen)_2_(phenpy)]­Cl_2_) as well as **Crddpd** ([Cr­(ddpd)_2_]­(BF_4_)_3_) and the synthesis procedures are given in the Supporting Information (Section S3. Particle Synthesis and Characterization).

### 2.2 Methods

Dynamic light scattering (DLS) and zeta potential (ζ-potential) measurements were performed using a Zetasizer Nano ZS (Malvern Panalytical, Germany) equipped with a 630 nm laser. Transmission electron microscopy (TEM) images were recorded on a F200S microscope (ThermoFisher Scientific, USA), while high-resolution TEM (HR-TEM), high-angle annular dark-field STEM (HAADF-STEM), and energy-dispersive X-ray (EDX) spectroscopy were carried out on a JEOL JEM-2800 S/TEM at the Electron Microscopy Facility at IST Austria. More details on the DLS and TEM measurements and data evaluation are provided in the Supporting Information.

PL spectra and PL decay curves used to determine the O_2_ shielding efficiency of the two different silica networks were obtained with a calibrated FSP920 fluorescence spectrometer (Edinburgh Instruments, UK) at room temperature (r.t.; *T* = 22 ± 2 °C) and fitted using FAST software (Edinburgh Instruments, UK), employing *X* ^2^ as the quality criterion for the fit evaluation for the Cr^III^ complex in solution and incorporated in silica NPs. For the measurement of the shorter PL decay times of **Ruphen** and **Ruphenpy** in solution at r.t., the lifetime spectrofluorometer FSP920 (Edinburgh Instruments, UK) was used. A detailed description of the fitting/analysis procedure used for the multiexponential PL decay kinetics of **Ruphenpy@ARGSiO**_**2**_ and **Ruphenpy@STOSiO**_**2**_ is provided in the Supporting Information (Section S4. Optical properties, subsection S4.1 Methods). All temperature-dependent PL spectra measurements were recorded with the spectrofluorometer FSP920 over a temperature range of 5–85 °C, using an equilibration time of 12 min for each temperature. The time interval between the temperature cycles did not exceed 20 min. The calculation of the thermometric performance parameters luminescence intensity ratio (LIR), relative sensitivity (S_r_), and thermal resolution or temperature uncertainty (Δ*T*) is detailed in the Supporting Information (Section S5. Temperature sensing properties).

### 2.3 Synthesis of the **Crddpd**, **Ruphen** and **Ruphenpy**-Stained Core and Core/Shell SiO_2_ NPs

Details on the syntheses of the SiO_2_ NPs stained with **Crddpd**, **Ruphen**, and **Ruphenpy** and their structure-analytical and spectroscopic characterization are summarized in Figure 1 and in the Supporting Information (Figures S4 and S5). For **Ruphen**, we solely focused on l-arginine-type SiO_2_ NPs. The synthesis of **Crddpd** was previously reported.^47,48^

## 3 Results and Discussion

Before exploring the general applicability of our previously developed complete oxygen shielding strategy relying on l-arginine silica networks to other transition metal complexes such as **Ruphen** and dyad **Ruphenpy** (Figure 1), we first determined the PL decay kinetics of both Ru^II^ compounds, displaying broad MLCT-type emission bands with maxima around ∼600 nm upon 450 nm excitation in air-and in Ar-saturated solution to evaluate our O_2_ removal procedure by flushing with Ar for 60 min. Time-resolved PL measurements (405 nm excitation) show monoexponential decays for **Ruphen** with lifetimes of ∼443 ns and ∼1 μs in air- and Ar-saturated water (Supporting Information, Figure S31a,b). The PL decay kinetics of **Ruphenpy** in aqueous solution are monoexponential with PL lifetimes of ∼1.6 μs and ∼57 μs in air- and Ar-saturated aqueous solution, respectively (Figure 2j). The long unquenched PL lifetime of **Ruphenpy** originates from a mainly pyrene-localized triplet state, populated by fast intramolecular Dexter-type energy transfer from the initially excited **Ruphen** unit in **Ruphenpy** (Figure 1, middle).^40,49^ The good match of the PL lifetime data in Ar-saturated solution with the previously reported lifetimes of 1 and 52.6 μs confirms the suitability of our O_2_ removal procedure.^40^ These PL lifetimes were subsequently used as reference values for Ru^II^ compounds, which are effectively protected from O_2_ quenching.

**Figure 2:**
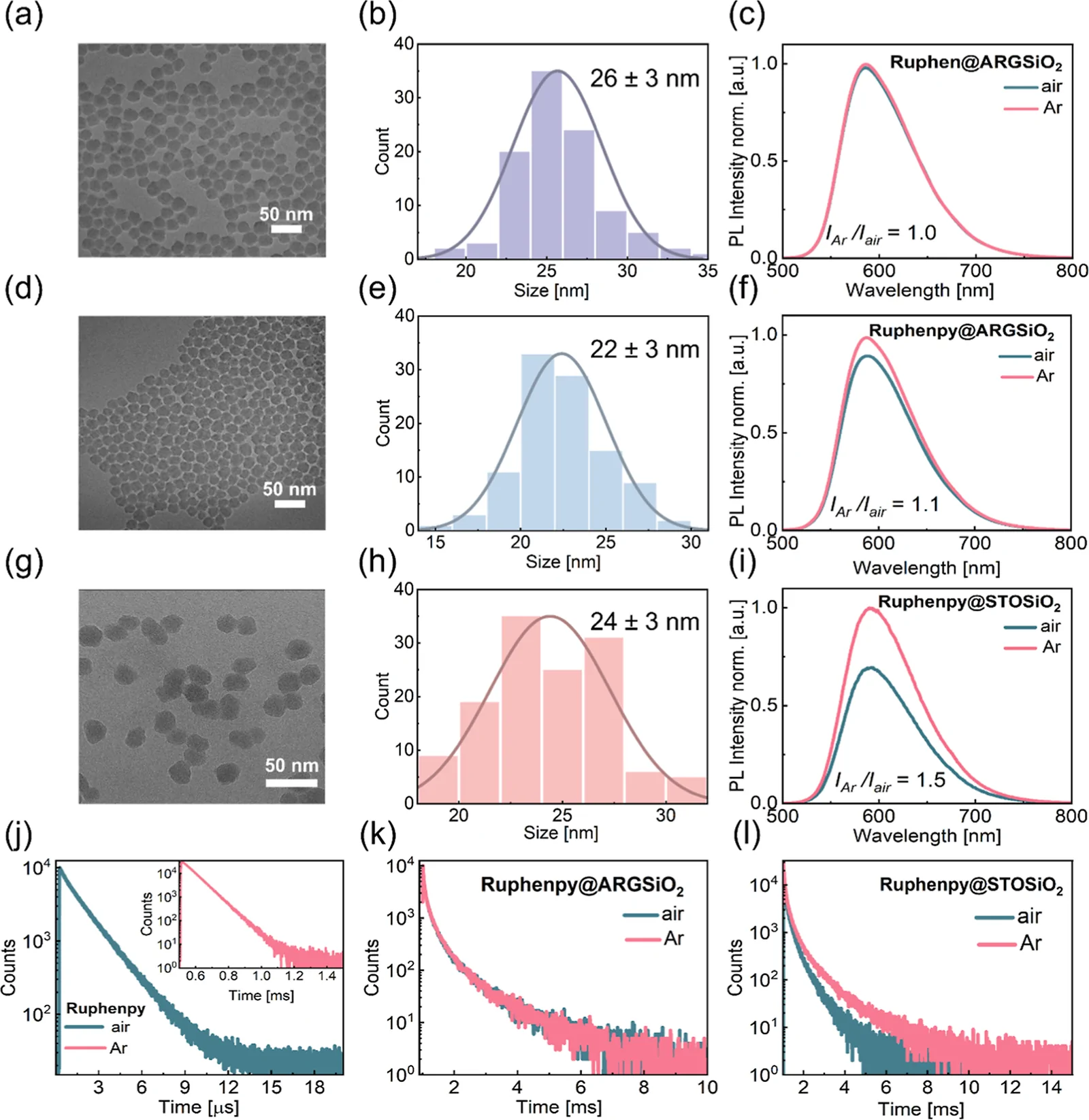
HR-TEM images of SiO_2_ NPs prepared with the l-arginine approach (**ARGSiO**_**2**_ NPs) loaded with **Ruphen** (**Ruphen@ARGSiO**_**2**_, top) and **Ruphenpy** (**Ruphenpy@ARGSiO**_**2**_, bottom). Scale bars: 50 nm. (b,e) TEM particle size distributions of the **Ruphen** and the dyad **Ruphenpy** loaded l-arginine SiO_2_ NPs derived from log–normal fits, yielding mean diameters of 26 ± 3 nm for **Ruphen@ARGSiO**_**2**_ and 22 ± 3 nm for **Ruphenpy@ARGSiO** _2_ . (c,f) Normalized PL spectra of **Ruphen@ARGSiO**_**2**_ and **Ruphenpy@ARGSiO**_**2**_ recorded under atmospheric (air-saturated, dark green) and anaerobic conditions (Ar-saturated, pink). The corresponding intensity ratios are *I*_Ar_/*I*_air_ = 1.0 and 1.1. (g) HR-TEM image of SiO_2_ NPs prepared with the Stöber approach (**STOSiO**_**2**_ NPs) loaded with **Ruphenpy** (**Ruphenpy@STOSiO**_**2**_). Scale bars: 50 nm. (h) TEM particle size distributions of **Ruphenpy**-loaded Stöber SiO_2_ NPs derived from Gaussian fits yielding mean diameters of 24 ± 3 nm for **Ruphenpy@STOSiO**_**2**_ (i) Normalized PL spectra of **Ruphenpy@STOSiO**_**2**_ recorded under atmospheric (air-saturated, dark green) and anaerobic conditions (Ar-saturated, pink). The corresponding intensity ratios are *I*_Ar_/*I*_air_ = 1.5. (j–l) PL decay kinetics of **Ruphenpy** (j) in water under atmospheric and anaerobic conditions (inset) and encapsulated in (k) l-arginine-type SiO_2_ NPs, and (l) Stöber-type SiO_2_ NPs in air- and Ar-saturated solution.

### 3.1 Assessing the Versatility of our SiO_2_-Encapsulation Strategy

Next, we incorporated **Ruphen** and **Ruphenpy** into an amorphous, nonporous SiO_2_ core and core/shell NPs using the respective chloride salts and the l-arginine-mediated biphasic sol–gel synthesis route, which had provided complete oxygen protection for the Cr^III^ complexes [Cr­(ddpd)_2_]­(PF_6_)_3_ and [Cr­(ddpd)_2_]­(BF_4_)_3_.^25^ We also prepared **Crddpd**-stained SiO_2_ NPs for comparative studies on the luminescence sensing behavior of metal complex-stained SiO_2_ NPs presented in a later section. The resulting metal complex-stained SiO_2_ NPs were characterized by electron microscopy, DLS, and zeta potential measurements, revealing TEM average particle diameters of 26 ± 3 nm, 22 ± 3 nm, and 23 ± 3 nm for the **Ruphen**, **Ruphenpy**, and **Crddpd**-stained l-arginine-mediated SiO_2_ NPs, respectively, along with a narrow size distribution, near-spherical morphology, and a smooth surface (Figure 2 panels a,b,d,e,g,h and Figures S6a,S8a,S9a in the Supporting Information). The strongly negative zeta (ζ)-potentials of the **Ruphen**, **Ruphenpy**, and **Crddpd**-stained l-arginine SiO_2_ NPs samples (−22.1 ± 1.2 mV, −22 ± 1.2 mV, and −23.8 ± 2.5 mV, respectively; Figures S12,S13 and S15 and Table S3 in the Supporting Information) indicate a high colloidal stability under the applied conditions. The unstained l-arginine SiO_2_ particles exhibited an average diameter of 24 ± 1 nm and a ζ-potential of −21.3 ± 3.9 mV. Interestingly, incorporation of **Ruphen**, **Ruphenpy**, and **Crddpd** barely affects the size, morphology (shape/surface roughness), and the negative surface charge of the SiO_2_ NPs. In addition, the negative ζ-potential values support the effective removal of free, positively charged Ru^II^ and Cr^III^ complexes via dialysis. In the present study, we did not determine pore-size distributions for all luminophore-loaded SiO_2_ as N_2_ sorption/BET analysis requires comparatively large amounts of dried material, which was not available for all stained nanoparticle samples. Moreover, the SiO_2_ NPs prepared by the Stöber and l-arginine routes are amorphous, non templated SiO_2_ nanomaterials and are not expected to contain ordered mesopores, as supported by our previous BET measurements of similarly prepared silica NPs.^25^

In addition, dyad **Ruphenpy** was embedded into SiO_2_ NPs using the conventional Stöber-type silica synthesis (**Ruphenpy@STOSiO**_**2**_, d_TEM_: 24.4 ± 2.9 nm) commonly used for the incorporation of such metal complexes into silica networks for comparison to literature systems^35,50^ and to our previously reported Cr^III^-stained Stöber-type SiO_2_ NPs.^25^ Also, unstained Stöber SiO_2_ NPs (**US-STO@SiO**_**2**_, d_TEM_: 24 ± 3 nm, ζ-potential of −32.1 ± 4.4 mV; synthesis scheme shown in the Supporting Information, Figure S1) were prepared following the same synthesis protocol to confirm that the particle size and size distribution of the SiO_2_ NPs are unaffected by particle staining. The very negative ζ-potential of **Ruphenpy@STOSiO**_**2**_ of −36.3 ± 0.7 mV (Supporting Information, Figure S14 and Table S3) indicates a high colloidal stability and complete removal of free positively charged Ru^II^ complexes by particle purification via dialysis. The observed differences in ζ-potential of the SiO_2_ NPs prepared by the l-arginine and Stöber method are attributed to the different surface chemistry resulting for the two sol–gel synthesis routes. Stöber SiO_2_ particles are formed by ammonia-catalyzed hydrolysis and condensation of TEOS in EtOH/H_2_O, whereas the l-arginine-derived particles are formed in a biphasic water/cyclohexane system using l-arginine as catalyst. These different reaction conditions can affect the number and accessibility of negatively charged silanolate groups at the particle surface.^51^

Successful loading of the l-arginine and Stöber SiO_2_ NPs with the Ru^II^ and Cr^III^ complexes was confirmed by ICP–MS measurements of dissolved luminophore-stained SiO_2_ NPs provided in the Supporting Information (Tables S1 and S2). The number of incorporated luminophores was estimated from the Cr or Ru content determined by ICP–MS, assuming one metal center per complex. Thereby, the amount of complex was divided by the calculated number of NPs, derived from the particle mass concentration, TEM diameter, and silica density. The incorporation efficiency was determined by comparing the amount of detected complex after purification with the amount initially used for synthesis. EM combined with EDX (Supporting Information, section on electron microscopy (EM) measurements) was employed in addition but not used for the determination of luminophore loading concentrations as the Ru Lα line is difficult to distinguish in EDX due to its low energy and its close proximity to the Cl Kα line at about 2.62 keV. Determination of the encapsulation efficiency of the **Ruphen**, **Ruphenpy**, and **Crddpd** in the l-arginine SiO_2_ NPs by ICP–MS (Table S2 in the Supporting Information) reveals about 2, 0.5, and 1 luminophores per SiO_2_ NP on average. This corresponds to an overall loading (incorporation) efficiency of about 19%, 29%, and 64% for **Ruphen@ARGSiO**_**2**_, **Ruphenpy@ARGSiO**_**2**_, and **Crddpd@ARGSiO**_**2**_, respectively. Successful loading of the Stöber SiO_2_ NPs with **Ruphenpy** was also confirmed by ICP–MS measurements (Supporting Information, Tables S1 and S2), yielding on average about 5 complexes per SiO_2_ NP. This equals a loading or incorporation efficiency of about 33%.

Next, the PL properties of the luminophore-stained SiO_2_ NPs and the O_2_ protection efficiency of the luminophores in the SiO_2_ NPs were assessed and quantified by steady-state and time-resolved PL measurements in air and Ar-saturated dispersions at room temperature.

As detailed in the Supporting Information (Section 4.1 methods), the O_2_ shielding efficiency of the different SiO_2_ NPs was quantified by comparing the ratio of the steady state and time-resolved PL signals measured for the same samples under ambient air and after purging with argon (Ar-saturated dispersions), with the latter serving as benchmark values for the respective nonquenched luminophore in a deoxygenated environment. For samples exhibiting multiexponential decay, the shielding efficiency is calculated from the integrated areas of the PL decay curves rather than from individual lifetime components. The PL lifetimes of the molecular systems in Ar-saturated solution were employed as reference values for completely protected Ru^II^ compounds.

The emission spectra of the molecular systems and **Ruphen@ARGSiO**_**2**_, **Ruphenpy@ARGSiO**_**2**_, and **Ruphenpy@STOSiO**_**2**_ in air- and Ar-saturated solution are shown in Figure 2. Encapsulation in l-arginine and Stöber SiO_2_ NPs results in slight spectral shifts of the emission bands of **Ruphen** and **Ruphenpy** from about 600 nm to about 590 nm (Supporting Information, Figure S38). This suggests small environment-induced changes in the energy landscape of the SiO_2_-matrix encapsulated Ru^II^ compounds and increased ^3^MLCT energies by about 0.035 eV. The size of the PL intensity changes upon O_2_ removal correlates with the O_2_ sensitivity of **Ruphen** and **Ruphenpy**.^40^ Whereas a conventional Ru^II^ complex such as **Ruphen** reveals only a 2-fold enhancement (*I*_Ar_/*I*_air_ ≈ 2) in Ar-saturated aqueous solution compared to air-saturated solution, the dyad shows a ∼20-fold higher luminescence intensity (*I*_Ar_/*I*_air_ ≈ 20) upon O_2_ removal. Encapsulation in l-arginine-type SiO_2_ NPs effectively protects both complexes from O_2_-induced PL quenching as revealed by the nearly identical PL intensities of **Ruphen@ARGSiO**_**2**_ and **Ruphenpy@ARGSiO**_**2**_ in air- and Ar-saturated solution (Figure 2, panels (c) and (f)), and the representatively shown PL decay curves closely match with the PL decay kinetics of **Ruphenpy@ARGSiO**_**2**_ in the presence and absence of O_2_ (Figure 2k). From the measured PL intensity ratios of the molecular Ru^II^ compounds in Ar-saturated solution (*I*_Ar_) and the encapsulated Ru^II^ compounds in air-saturated solution (*I*_air_) of 1.01 and 1.1, the O_2_ protection efficiency of the l-arginine-type silica network was calculated to 99.0% and 90.9% for **Ruphen@ARGSiO**_**2**_ and **Ruphenpy@ARGSiO**_**2**_, respectively. In contrast, Stöber-type SiO_2_ NPs provide only partial shielding of the Ru^II^ complexes from O_2_ quenching, as reported in the literature for such silica networks.^35,50^ For **Ruphenpy@STOSiO**_**2**_, the intensity ratio *I*_Ar_/*I*_air_ of 1.5 corresponds to an O_2_ protection efficiency of 69.4%, confirming the previously observed reduced O_2_ protection efficiency of this type of silica network for the Cr^III^ complex [Cr­(ddpd)_2_]­(PF_6_)_3_ of 50%. Time-resolved PL measurements of air-saturated aqueous dispersions of **Ruphen@ARGSiO**_**2**_ reveal biexponential decay kinetics of encapsulated **Ruphen** and yield an average PL decay time of 3 μs. This value exceeds the previously reported lifetime of **Ruphen** of 1.1 μs in Ar-saturated water by a factor of almost three and implies complete O_2_ shielding in the l-arginine-type SiO_2_ NPs. The additional enhancement is tentatively attributed to the rigid silica matrix and the rigidification of **Ruphen** prolonging the PL lifetime. For time-resolved PL measurements of **Ruphenpy** in different environments, the time window to record the full PL decays had to be considerably expanded as O_2_ removal and encapsulation into silica matrix led to an increase of the time window from about 20 μs in air-saturated water to about 1 ms for the free dyad in Ar-saturated solution and about 10 ms for **Ruphenpy@ARGSiO**_**2**_ in air (Figure 2, panels (j) and (k)). This reflects the strongly prolonged PL lifetimes under conditions where PL quenching by O_2_ is prevented. As displayed in Figure 2 (panel (k)) for **Ruphenpy@ARGSiO**_**2**_, multiexponential PL decay kinetics are obtained (4-exponential reconvolution fit was applied) that indicate a distribution of emitting species or microenvironments rather than a single well-defined species/environment. The four-exponential fits present empirical descriptions of the heterogeneous decay kinetics. The different PL components are not assigned to specific molecules or environments within the SiO_2_ NPs as they may arise from several effects. This includes different pyrene–metal complex separations, pyrene/Ru complex conformations, dye localization, aggregation state, and heterogeneous environments within the SiO_2_ NPs. Therefore, no attempts were made to calculate O_2_ protection efficiencies from PL lifetime measurements, and PL lifetime measurements were not used for temperature-sensing studies detailed in the following section. Instead, we compared the integrated areas under the PL decay curves measured in air and under Ar atmosphere to derive O_2_ protection efficiencies from time-resolved PL measurements. This approach, which was also employed for lifetime-based temperature measurements, was later validated by comparison with PL intensity measurements and the results of fitted PL decay kinetics. The calculated Ar/air ratio of 0.99 derived from the almost identical PL decay profiles in the presence and absence of O_2_ indicates complete protection from PL quenching by O_2_. In contrast, for **Ruphenpy** encapsulated in Stöber-type SiO_2_ NPs (**Ruphenpy@STOSiO**_**2**_), the integrated PL decay areas markedly differ between Ar- and air-saturated dispersions, yielding an Ar/air ratio of 1.9 (Figure 2, panel (l)). This corresponds to an O_2_ shielding efficiency of about 50%. Please note that the O_2_ shielding efficiency calculated from the integrated PL decay curves and from steady-state PL measurements is expected to yield values that closely match within the respective measurement uncertainties, but not identical values. The uncertainties of these measurements are estimated to be in the order of 5%.

As in our previous work, **Ruphenpy**-containing core–shell architectures were also prepared by l-arginine-mediated silica regrowth, yielding either core-loaded particles with an unstained shell **(Ruphenpy@ARGSiO**_**2**_ **)@(US-ARGSiO**_**2**_ **)**, or unstained cores with a **Ruphenpy**-containing shell **(US-ARGSiO**_**2**_ **)@(Ruphenpy@ARGSiO**_**2**_ **)** (see Supporting Information for DLS: Figure S11; ζ-potential: Figure S16; TEM: Figures S26–S28). ICP–MS revealed a more efficient Ruphenpy incorporation during shell growth, with an average loading concentration of 27 complexes per particle for the shell-stained sample. Ar/air comparisons showed highly efficient O_2_ shielding for these particles, with ratios in the measured PL intensity or integral PL decay of about 99% as derived from steady-state intensity and time-resolved decay-area analysis (Figure S30a,c). For the core-loaded particles coated with an unstained shell, the steady-state air/Ar signal ratio amounted to about 95% and 84% (Figure S30b,d) for the integral PL intensity and PL decay curves. Possible reasons for the lowered protection for **(Ruphenpy@ARGSiO**_**2**_ **)@(US-ARGSiO**_**2**_ **)** could be the trapping of residual O_2_ in the SiO_2_ NPs during SiO_2_ core formation or changes in the local luminophore environment caused by the additional shell-growth step.

### 3.2 Influence of l-Arginine SiO_2_ NPs Encapsulation on the Photostability of the Ru^II^ Complexes

The molecular ruby **Crddpd** is characterized by an outstanding inherent photostability, such that decomposition issues are essentially absent even upon prolonged irradiation with intense light sources.^47,52^ In contrast, tris­(diimine) complexes of Ru^II^ are prone to photodegradation. Mechanistically, this is caused by dissociation of the bidentate ligand upon thermal population of higher-lying metal-centered triplet states ^3^MC, see energy diagram in the right panel in Figure 3b.^53–57^ As the resulting bis­(diimine) solvent ruthenium­(II) complexes do not show the characteristic ^3^MLCT emission after photosubstitution, the loss in PL intensity can be used to monitor complex photostability.^58–60^ The photostability studies were performed with a beam-expanded blue cw laser to ensure well-defined homogeneous excitation at an intensity that does not permit two-photon absorption processes (447 nm, 0.5 W cm^–2^, 20 °C, see Supporting Information for a description of the setup and experimental details).^61^ In these measurements, the ^3^MLCT emission at ∼600 nm was monitored for diluted aqueous Ru^II^ complex solutions as a function of illumination time. As first experiments with **Ruphenpy** in air-saturated solution indicated the formation of a new emitting species resulting from the reaction between **Ruphenpy** and reactive oxygen species produced by the quenching of the Ru^II^-centered triplet state by O_2_ (Figures S35–S37), all subsequent photostability measurements were performed in Ar-saturated solution under conditions that ensured a homogeneous light absorption and circumvention of an illumination-induced increase in temperature (the absorption at the laser excitation wavelength was always below 0.1, see Supporting Information for the corresponding UV/vis spectra). Under these conditions, the decrease in ^3^MLCT luminescence directly reflects the relative photodegradation quantum yields. CW laser illumination of **Ruphen** in aqueous solution for 110 min leads to a reduction in PL intensity by about 50%, indicating efficient photodecomposition. However, similar photostability studies with **Ruphen** encapsulated in l-arginine-type SiO_2_ NPs, revealed a considerably enhanced photostability of **Ruphen** by a factor of more than five as reflected by a PL intensity decrease of only 10% under similar conditions (Figure 3a).

**Figure 3:**
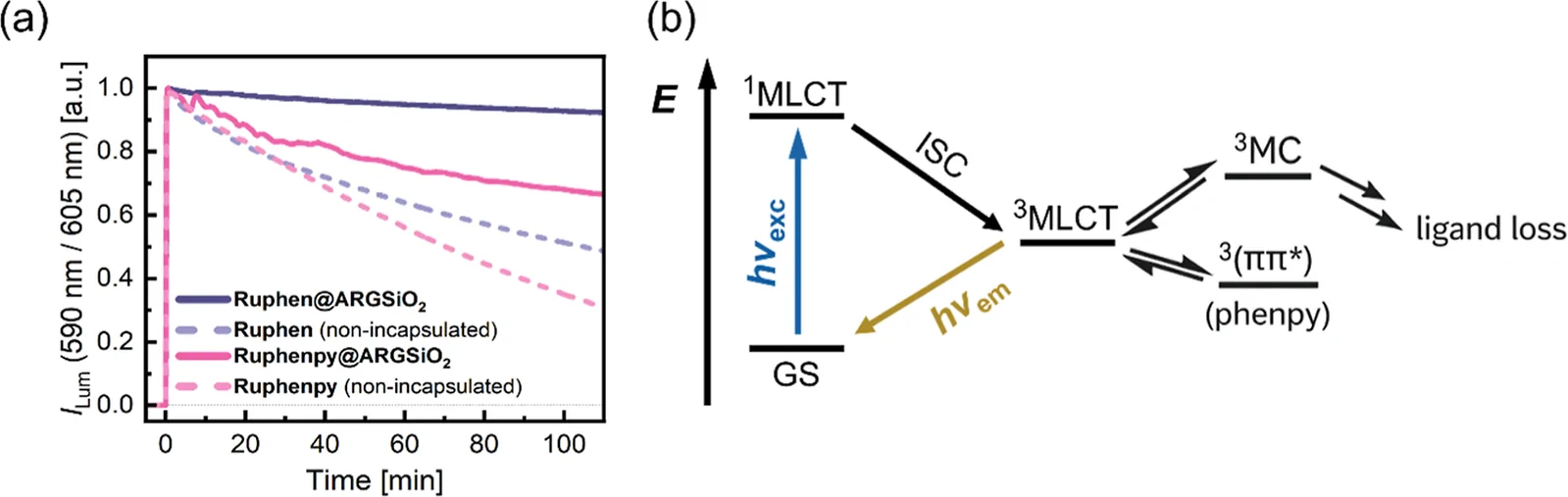
(a) PL stability measurements of nonencapsulated (3 μM) and l-arginine SiO_2_–NP-encapsulated **Ruphen** and **Ruphenpy** complexes in Ar-saturated aqueous solutions/dispersions under cw laser excitation at 447 nm (0.5 W cm^‑2^) using the setup shown in Figure S32. The temporal evolution of the normalized emission intensity at 590 nm for **Ruphen@ARGSiO**_**2**_ and at 605 nm for nonencapsulated **Ruphen**, **Ruphenpy**, and encapsulated **Ruphenpy@ARGSiO**_**2**_ solutions/dispersions during illumination. (b) The simplified energy-level diagram of **Ruphenpy**, indicating the deactivation pathways and photodegradation following photoexcitation. The corresponding UV/vis absorption spectra are shown in the Supporting Information. The concentration of the nonencapsulated Ru^II^ compounds used as chloride salts was 3 μM.

**Ruphenpy** is even much less photostable than **Ruphen** as the efficiency of the photodegradation of Ru^II^ complexes by ligand dissociation frequently increases with the triplet lifetime.^55,60^ As depicted in Figure 3b, the lowest triplet state of **Ruphenpy** is a long-lived pyrene-localized ^3^(π–π*) state,^40^ which is in a fast equilibrium with the emissive ^3^MLCT state at room temperature, that is the starting point for the thermal population of dissociative ^3^MC states.^54,57,62^ Photostability studies with **Ruphenpy** in Ar-saturated solution and encapsulated in l-arginine NPs also revealed a beneficial effect of the silica network on dyad photostability. Under similar conditions as used for the photostability studies of **Ruphen**, the dyad shows 70% photodegradation (Figure 3a). The photodegradation was reduced by a factor of 2 (35% instead of 70%, see Figure 3a) and Figures S33 and 34 in the Supporting Information for **Ruphenpy** incorporated in l-arginine silica NPs. This is very encouraging, considering the extremely long lifetime of the Ru^II^-dyad under these conditions. Overall, our findings agree well with photostability studies of well-explored [Ru­(bpy)_3_]^2+^ (bpy = 2,2'-bipyridine) and related compounds incorporated into SiO_2_ NPs.^35,50,63^ A likely explanation for the significantly improved photostability is the rigid structure of the silica network, which considerably hampers elongation of the Ru–N bonds required for ligand dissociation and facilitates recoordination of dissociated ligands. These findings demonstrate that encapsulation in l-arginine-type SiO_2_ NPs improves the photostability of photolabile Ru^II^ compounds, which, together with the strongly increased PL efficiency in air, improves and expands the application potential of such Ru^II^ complexes. Based on the excellent O_2_ shielding efficiencies of **Ruphen@ARGSiO**_**2**_ and **Ruphenpy@ARGSiO**_**2**_ (shown in Figure 2), we believe that ROS-induced decomposition, which has been observed for the unprotected dyad in homogeneous solution, see Supporting Information, Figure S36) only plays a minor role for **Ruphenpy** encapsulated in l-arginine-type SiO_2_ NPs. Therefore, we regard the above-mentioned ligand loss as the only decomposition pathway for the encapsulated compounds.

### 3.3 Luminescence Temperature Sensing with Nonencapsulated and SiO_2_-Embedded **Ruphen** and **Ruphenpy** in the Presence and Absence of Oxygen

Transition metal compounds such as Ru^II^ complexes are frequently used for contactless luminescence-based measurements of local temperature in air-saturated environments in the life and material sciences, thereby exploiting temperature-induced changes in PL intensity and lifetime, governed by the temperature dependence of PLQY in the respective environment.^16,39^ To minimize PL quenching by O_2_, which can interfere with temperature sensing, Ru^II^ compounds are typically encapsulated in O_2_ shielding matrices.^12,29,35,36^ Literature data show that SiO_2_-based matrices often do not provide efficient, i.e., complete O_2_ shielding. In several sol–gel/ormosil systems, the reported Stern–Volmer constants indicate a substantial O_2_ accessibility of the ruthenium luminophore to O_2_ PL quenching. This makes these systems better suited for O_2_ sensing than for O_2_-independent thermometry. In contrast, Wencel et al. reported almost O_2_-insensitive TEOS-based SiO_2_ NPs, which exhibit PL intensity ratios of *I*_0_/*I*_20_ = 1.01, corresponding to a 99% emission retention at an O_2_ concentration of 20% (oxygen fraction in the O_2_/N_2_ gas mixture applied to the aqueous nanoparticle suspension), while TEOS–MTEOS hybrid particles showed a higher O_2_ permeability.^35^ Polymer matrices such as PVA and PCA were reported to effectively suppress O_2_ cross-sensitivity. In these cases, the observed O_2_ shielding was only qualitatively described rather than by Stern–Volmer constants.

However, the O_2_ shielding efficiency of most matrices has not been quantified and is often not complete, i.e., quantitative. In addition, also a possible influence of the encapsulation chemistry and the matrix’s composition, structure, and rigidity on luminophore temperature sensitivity has to be considered.^16^ To explore the applicability of our SiO_2_ shielding approach for PL thermometry with O_2_-sensitive molecular compounds in air and to assess possible matrix effects arising from the different silica networks, we investigated the temperature dependence of the PL properties of free and SiO_2_ NP-encapsulated **Ruphen** and **Ruphenpy**. Nanoparticles prepared by both the l-arginine and Stöber sol–gel routes were studied over the temperature range of 5–85 °C by steady-state and time-resolved PL spectroscopy in air- and Ar-saturated solutions. All measurements were performed in three successive heating–cooling cycles of thermally equilibrated samples. Deliberately, no measures were taken to ensure that the initial O_2_ concentration present for the first cycles with air-saturated solutions was restored for the starting conditions of the lowest temperature of 5 °C. These experimental conditions were chosen to model those used for temperature sensing in many life sciences studies, in which the PL response of the O_2_-sensitive luminophore is affected by temperature as well as by O_2_ concentration, and to highlight the importance of complete O_2_ shielding. Please note that the temperature-dependent solubility of O_2_ in water and other environments leads to a steady decrease of O_2_ concentration with increasing temperature, resulting in reduced PL quenching. Simultaneously, the increase in temperature favors a reduction of luminophore PLQY and PL lifetime. For temperatures ≥65 °C, O_2_ is expected to have almost completely disappeared from solution. These opposite effects of temperature and temperature-dependent O_2_ concentration affect the measured PL response of O_2_-responsive luminescence thermometers, especially in the first heating cycle, while in the second and third heating cycles, the O_2_ concentration is reduced compared to the first heating cycles as back diffusion of O_2_ into solution is slow. Hence, the influence of O_2_ sensitivity and O_2_ shielding efficiency on PL temperature sensing is best reflected by the results of the first heating cycle and the comparison of air- and Ar-saturated solutions and l-arginine and Stöber type SiO_2_ NPs. As highlighted in Figure 4 for **Ruphenpy**, chosen for detailed investigation across different environments, the third heating cycle and the corresponding measurements of the nonencapsulated compound under an Ar atmosphere provide insights into the influence of O_2_, the shielding efficiency of the SiO_2_ matrix, and matrix effects on the dyad́s temperature sensitivity. The reversibility of the samples follows, e.g., from a comparison of the PL spectra and PL intensities of the O_2_-protected systems. Temperature-dependent PL studies of less O_2_- sensitive **Ruphen** embedded in l-arginine SiO_2_ NPs, recorded over three consecutive heating cycles, are provided in the Supporting Information (Figures S56 and S57) and reveal similar trends.

**Figure 4:**
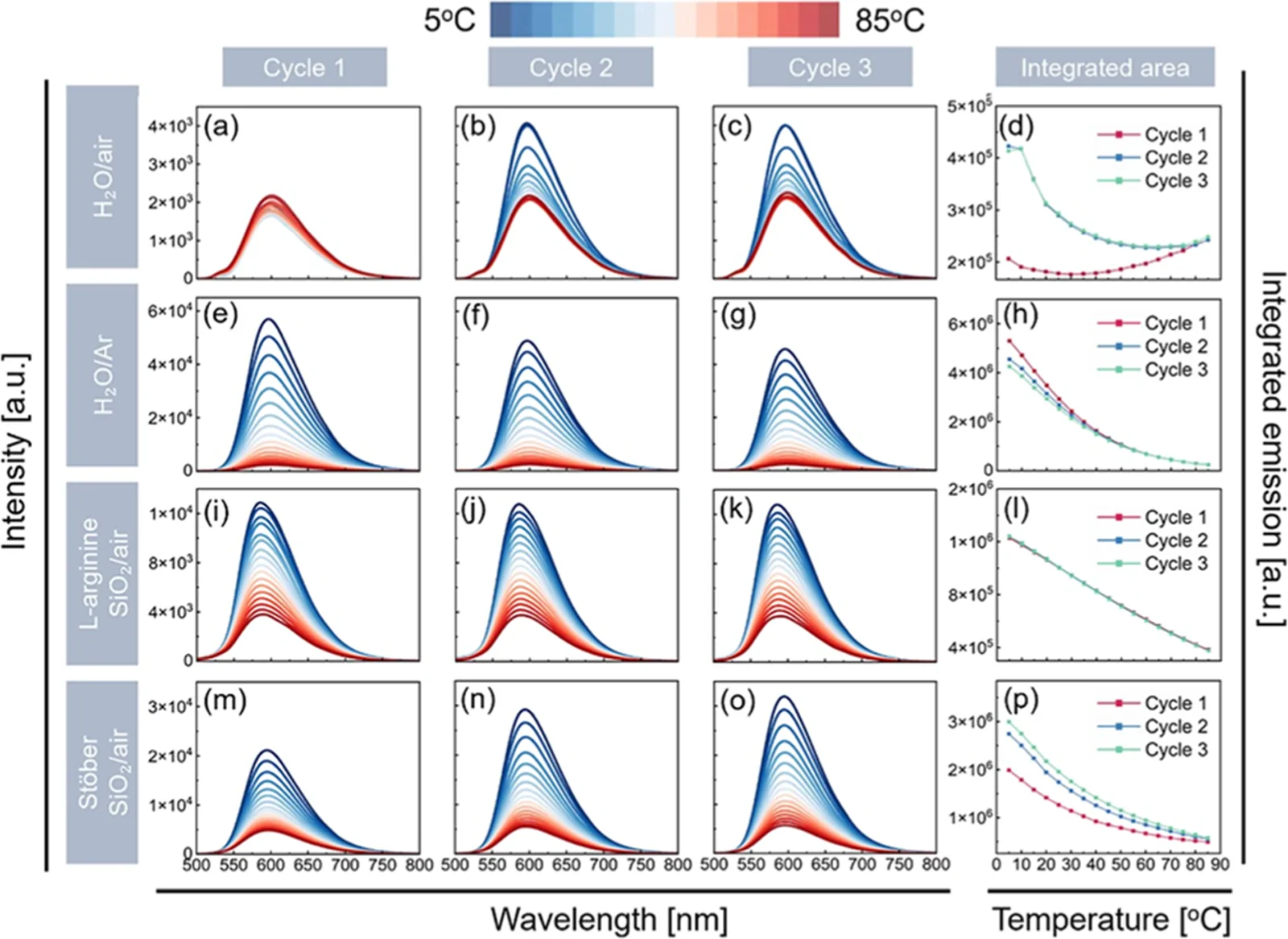
(a–d) Temperature-dependent PL spectra of the **Ruphenpy** complex in H_2_O/air recorded over three consecutive heating–cooling cycles. The corresponding integrated emission areas for each cycle are provided in the right column. (e–h) Analogous data for H_2_O/Ar. (i–l) Temperature-dependent PL spectra and integrated emission intensities of **Ruphenpy@ARGSiO**_**2**_ in aerated aqueous dispersion; (m–p) corresponding data for **Ruphenpy@STOSiO**_**2**_. The integration limits were set from 500 to 800 nm. Aiming for a comparison of environment effects on the temperature dependence of the integral PL of the **Ruphenpy** complex in different environments, we chose a constant wavelength scale to assess the temperature-dependence of the integral PL of the **Ruphenpy** complex in different environments and not an energy scale. The equilibration time for each temperature was 12 min. After returning to 5 °C, a break of about 20 min was applied before starting the next heating–cooling cycle.

#### 3.3.1 PL Intensity-Based Temperature Sensing

The temperature-dependent PL intensity measurements with the strongly O_2_-responsive dyad **Ruphenpy** in different environments (Figure 4) suggests two competing effects: thermally induced PL quenching, which decreases the emission intensity, and a temperature-induced decrease in dissolved O_2_ concentration, which reduces O_2_ quenching. The latter results in an increase in PL intensity. This follows from a comparison of the initial and finally reached PL intensities for the three sequentially performed measurement cycles for temperatures ≤65 °C.

The influence of O_2_ is most pronounced for the first heating cycle for conditions, where **Ruphenpy** is not completely protected from O_2_ quenching, i.e., for the dyad in air-saturated water and encapsulated in Stöber-type SiO_2_ NPs and for temperatures between 5 °C and≤ 65 °C, where the solubility of O_2_ is higher compared to temperatures ≥65 °C. Due to heating-induced O_2_ depletion, the PL intensities steadily increase from cycle to cycle for temperatures between 5 °C and ≤65 °C and are already identical for the second and third heating cycles. For temperatures ≥65 °C, the PL intensities match across all heating cycles. On the contrary, under conditions where **Ruphenpy** is completely protected from O_2_ quenching, i.e., for measurements under an Ar atmosphere or for **Ruphenpy** encapsulated in l-arginine-type SiO_2_ NPs, the PL intensities do not vary between cycles. The minimal changes in initial PL intensity observed for the former system are ascribed to traces of O_2_ introduced by back diffusion of O_2_ due to the long measurement time of about 13 h for all three cycles.

The temperature-dependent changes in integral PL provided in the right panels of Figure 4, derived from the three heating cycles, also highlight the strong influence of O_2_ on PL temperature sensing for conditions where the PL of **Ruphenpy** can be quenched by O_2_. Especially promising for PL temperature sensing is the almost linear temperature dependence of the PL intensity of **Ruphenpy@ARGSiO**_**2**_, see also next section. Overall, the generally observed matching PL intensities and PL spectra for temperatures ≥65 °C for all three heating cycles and the good match of the PL data at lower temperatures, e.g., at 5 °C reached for cycles 2 and 3 in Figure 4 indicate a good thermal stability of the dyad and the dyad-stained SiO_2_ NPs. In addition, the results, representatively displayed in Figure 4 for **Ruphenpy**, clearly demonstrate the importance of separating temperature and oxygen-induced PL changes in PL intensity for PL thermometers with O_2_-sensitive single-band luminescence.

To facilitate a comparison of the measured PL intensity-based temperature responses, the integrated PL in Figure 4d,h,l,p were normalized to the value at 5 °C for each individual heating cycle. The relative decrease between 5 and 85 °C was calculated using eq 11 (Supporting Information, Section S5.3). The normalized plots are shown in Figure S54e–h, and the extracted values are summarized in Table S15. These results confirm the strong influence of the surrounding medium on **Ruphenpy**’s intensity-based PL response. In air-saturated water, the first cycle shows an apparent intensity increase, corresponding to a relative change of −17%, while cycles 2 and 3 stabilize at 40–43%. In contrast, **Ruphenpy@ARGSiO**_**2**_ displays a highly reproducible response of 69–70% over all cycles. **Ruphenpy@STOSiO**_**2**_ shows a stronger but slightly less stable apparent decrease of 75–81%. The largest relative decrease is observed in Ar-saturated water, reaching 94–96%; however, the requirement for strict O_2_ exclusion limits the practical applicability of the unprotected dyad under these conditions.

#### 3.3.2 Lifetime-Based Temperature Sensing

For single-emission-band sensors lacking an internal reference, PL lifetime sensing is often preferred over PL intensity measurements since the PL intensity can vary with excitation power, collection efficiency, and luminophore concentration. However, in O_2_-responsive systems, O_2_ quenching affects the PL lifetime in the same way as PL intensity, implying that a PL lifetime readout is likewise susceptible to changes in dissolved O_2_ concentration. Therefore, quantitative O_2_ shielding is also essential for reliable lifetime-based PL thermometry. However, in more complex environments such as SiO_2_ NPs, luminophores often reveal complex decay kinetics due to the heterogeneous luminophore microenvironment (see: PL decay curves of **Ruphenpy** embedded in l-arginine SiO_2_ NPs shown in Figure 2k and in the Supporting Information, in Figures S59 and S60). For luminophores in matrices providing complete O_2_ protection, such as l-arginine SiO_2_ NPs, this can hamper simple PL lifetime sensing approaches, which commonly require monoexponential decay kinetics. For **Ruphenpy@ARGSiO**_**2**_ in aerated aqueous dispersion, we pursued two approaches. One approach relies on average PL lifetimes ⟨τ⟩ calculated from 4-exponential reconvolution of the fitted PL decay curves measured at different temperatures (Supporting Information, Section S4.1 methods subsection in Section 4. Optical properties). The results are displayed in Figure 5a; please note that this fitting approach presents only an empirical description of the PL decay shape, and we therefore did not attempt to assign the obtained lifetime components to luminophore molecules in a specific environment or under specific conditions. In addition, we explored the applicability of integral PL decay curves derived from the integration of the normalized PL decay curves measured at different temperatures. While ⟨τ⟩ primarily reports on the kinetic time scale, the integrated PL decay area is directly related to the total number of detected photons and correlates with PL QY for identical excitation and detection conditions. The results of this simpler, yet underexplored approach are shown in Figure 5b. As follows from Figure 5, both approaches yield closely matching trends and agree well across repeated heating cycles. Also, both approaches closely match the trend observed for the integral PL spectra shown in Figure 4 (panel (l)). This is demonstrated in Figure 5c, which displays the linear correlation between the integral PL intensities and integral PL decay curves. This confirms the applicability of our integral PL decay curve approach.

**Figure 5:**
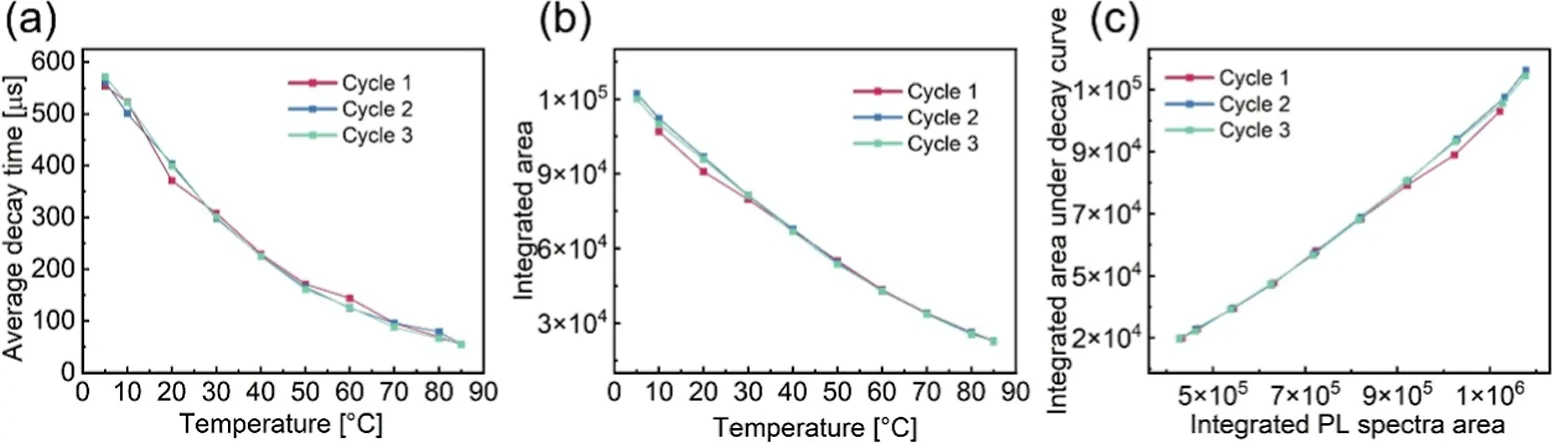
Lifetime-based temperature sensing with **Ruphenpy@ARGSiO**_**2**_ in air-saturated solution. (a) average decay time ⟨τ⟩ derived from a 4-exponential reconvolution fit. (b) Integral PL decay curves obtained by integrating the area below the normalized PL decay curves. (c) Correlation between the temperature dependence of the integral PL decay curves and the integral PL intensities measured for three heating cycles.

### 3.4 Luminescence Temperature Sensing with Dual Emissive Cr^III^ Complexes in the Presence and Absence of Oxygen

Especially attractive for contactless, minimally invasive PL temperature measurements are so-called Boltzmann thermometers, which rely on the temperature-dependent relative population ratio of two closely spaced, thermally equilibrated excited electronic states of a luminescent material.^64^ The energy difference (Δ*E*) between these spectrally distinguishable emissive states is commonly in the order of the thermal energy.^64^ The PL bands for these two close-lying states must be sufficiently small to allow for reliable discrimination. This provides the basis for ratiometric PL temperature measurements, directly probing the temperature-dependent population distribution of these excited states governed by the Boltzmann distribution, independent of fluctuations in the luminophore concentration, overall PL intensity and excitation intensity. This is often only feasible in a limited temperature operating range^15^ or can require special fitting models to exploit a higher energy gap for high relative sensitivities at appreciably low temperatures.^65^ Examples of dual emissive Boltzmann thermometers that span the visible and NIR spectral range and are highly susceptible to temperature variations are lanthanide-based nanoparticles and phosphors containing, e.g., Er^III^, Nd^III^, and Pr^III^ as well as phosphors relying on Cr^III^ in strong ligand fields.^15,64,66–68^ However, molecular species rarely display such behavior. Exceptions include Cr^III^ complexes such as the first generation of the molecular rubies, where earth-abundant Cr^III^ is in a very strong ligand field and exhibits dual NIR PL over a wide temperature range.^43,69^

Cr^III^ complexes with different counteranions and varying solubility have been previously examined by us in environments varying in O_2_ solubility and permeability including organic and aqueous solvents, polystyrene nanoparticles (PSNP), and SiO_2_ NPs prepared by the l-arginine and Stöber sol–gel synthesis routes.^43,48,70^ Temperature sensing with dual emissive Cr^III^ complexes has been demonstrated by us before in different environments varying in O_2_ concentration or permeability, as well as composition, morphology, and rigidity,^43,48,70^ yet not in silica networks. Hence, we also assessed the temperature sensing performance of **Crddpd** in air- and Ar-saturated solution as well as encapsulated in l-arginine- and Stöber-type SiO_2_ NPs in air-saturated dispersion in three analogously performed heating cycles and evaluated the corresponding changes in integral PL intensity.

As shown in Figure 6a–d, repeated heating–cooling cycles in environments allowing for O_2_ quenching of the **Crddpd** luminescence revealed similar trends of the measured PL intensities, as previously observed for **Ruphenpy**. For Cr^III^ complexes, which are not completely shielded from O_2_, the PL intensity strongly changed from cycle to cycle, albeit with intensity trends different from those as observed for the Ru^II^ dyad (Figure 4). However, the spectrally well-separated PL bands at 738 and 775 nm are similarly affected, rendering ratiometric temperature sensing with Cr^III^-based systems O_2_-independent. However, the O_2_ concentration-dependent decrease in the intensities of both PL bands of **Crddpd** at temperatures ≤65 °C is likely to reduce the relative sensitivity of temperature measurements with such Cr^III^ complexes, as detailed in a subsequent section. For **Crddpd** protected from O_2_ quenching, i.e., under Ar atmosphere or encapsulated in l-arginine type SiO_2_ NPs, the cycle-dependent intensity variations are strongly reduced compared with unprotected **Crddpd** in air-saturated water, although small residual differences can still be observed. These remaining differences may originate from thermal-history-related effects, minor changes in the local microenvironment during repeated heating, or, in the case of Ar-saturated water, traces of O_2_ introduced during the long measurement time. To further quantify the magnitude and reproducibility of the intensity-based temperature response, the integrated PL-intensity areas were normalized to the value obtained at 5 °C for each heating cycle, and the relative decrease between 5 and 85 °C was calculated (Figure S54a–d and Table S15). In air-saturated water, the relative decrease increases from 64% to 78% over three cycles. This confirms a cycle-dependent intensity response. In contrast, the behavior of **Crddpd@ARGSiO**_**2**_ is reproducible with a constant decrease of 56% in all cycles. **Crddpd@STOSiO**_**2**_ exhibits a lower first-cycle response of 49%, followed by higher values of 67–69% in cycles 2 and 3. The most pronounced temperature-induced PL quenching is observed in Ar-saturated water, with an 86–87% decrease.

**Figure 6:**
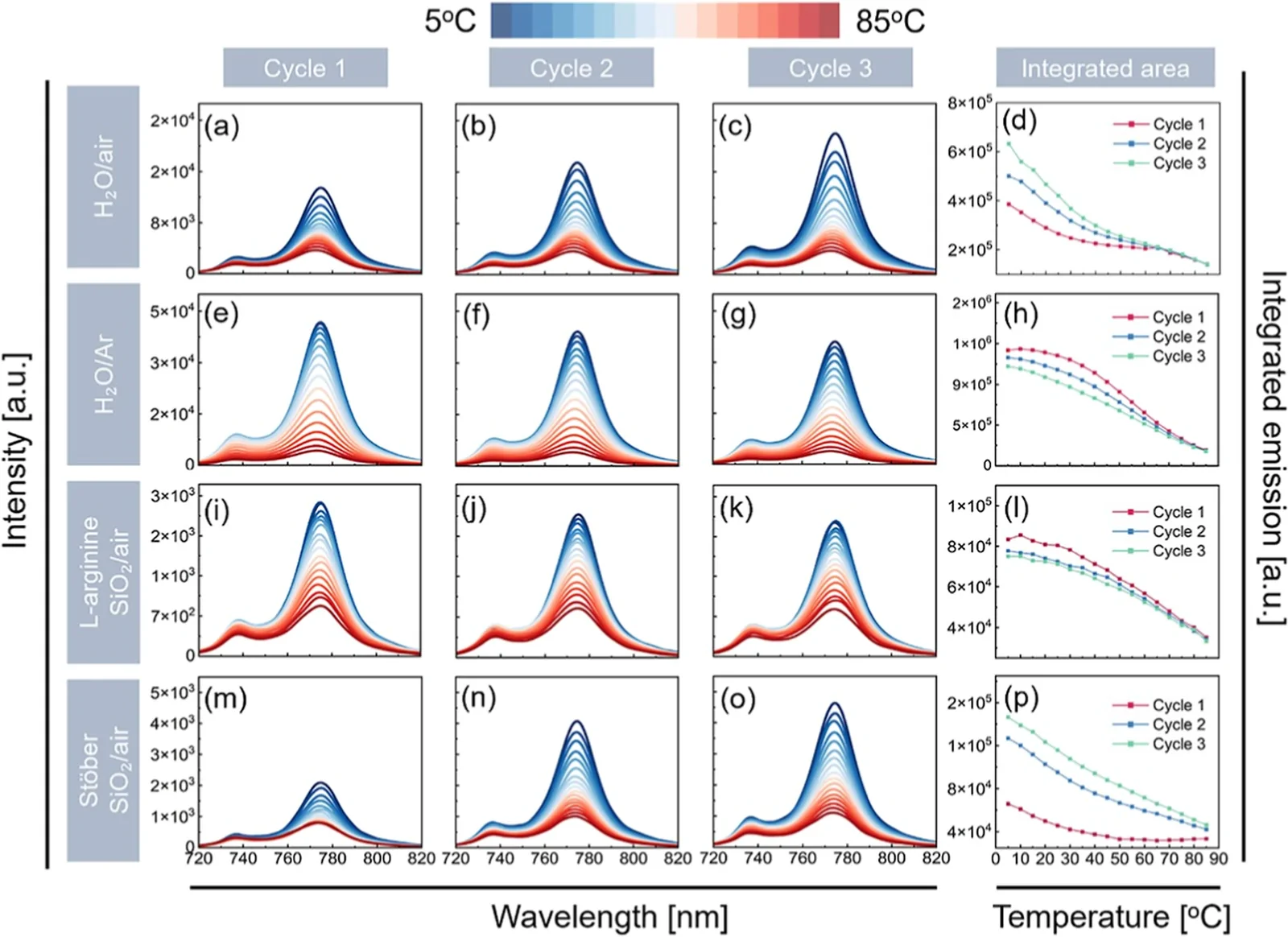
Temperature-dependent PL spectra of the **Crddpd** complex in H_2_O/air recorded over three consecutive heating–cooling cycles, with the corresponding integrated PL for each cycle. (e–h) Analogous data for H_2_O/Ar. (i–l) Temperature-dependent PL spectra and integrated emission areas of **Crddpd@ARGSiO**_**2**_ in aerated aqueous dispersion. (m–p) Corresponding spectra and integral emission intensities for **Crddpd@STOSiO**_**2**_ in air. The integration limits were set from 720 to 820 nm. Aiming for a comparison of environment effects on the temperature dependence of the integral PL of the Cr^III^ complex in different environments, we chose a constant wavelength scale to assess the temperature-dependence of the integral PL of the Cr^III^ complex in different environments and not an energy scale. The equilibration time for each temperature was 12 min. After returning to 5 °C, a break of about 20 min was applied before starting the next heating cycle.

The corresponding Boltzmann plots (slopes: H_2_O/air: −883 ± 2 K; H_2_O/Ar: −885 ± 2 K), yielding energy gaps (Δ*E*) of 614.5 ± 0.5 cm^–1^, are shown in the SI (Figures S40 and S42; Tables S4 and S5). Encapsulation in silica networks slightly affects the temperature response and the energy gaps. **Crddpd@ARGSiO**_**2**_ displays a slope of −824 ± 8 K and an energy gap Δ*E* of 573 ± 6 cm^–1^, and **Crddpd@STOSiO**_**2**_ a slope of −859 ± 8 K and an energy gap of 597 ± 6 cm^–1^. The Boltzmann slope scales with −Δ*E* and the less negative slopes of **Crddpd@ARGSiO**_**2**_ and **Crddpd@STOSiO**_**2**_ indicate a smaller effective energy separation between the thermally coupled states governing the readout band ratio. This likely reflects an influence of the microenvironment within the silica particles originating from modified crystal fields, and interactions with the heterogeneous silica network acting as counteranions for the Cr^III^ complex.

### 3.5 Thermometric Performance Parameters Luminescence Intensity Ratio, Relative Sensitivity

For PL (nano)­thermometry, ratiometric approaches are widely applied that are less affected by luminophore concentration compared to thermal sensing based on PL intensity measurements. Examples include lanthanide NPs, that exploit the PL of lanthanide ions with thermally coupled energy levels separated by an energy gap in the range of 200 cm^–1^ to 2000 cm^–1^, such as the two green-emitting energy levels of Er^3+^, which are referred to as Boltzmann thermometers.^68^ The thermometric performance of PL nanothermometers such as lanthanide NPs and Cr^III^- and transition metal ion-containing phosphors is commonly quantified by different thermometric parameters.^15,64,66–68^ This includes the relative thermal sensitivity (S_r_), which correlates the change in PL intensity ratio (LIR) with a temperature change and is used to assess and compare the performance of different PL (nano)­thermometers. In addition, the thermal resolution or temperature uncertainty (δT) is used. δT represents the minimum statistically achievable temperature change that can be resolved. However, for molecular PL thermometers, commonly LIR, S_r_, and δT values are barely reported. Moreover, it is important to note that the overall achievable precision of a luminescent (Boltzmann) thermometer is a compromise between the appreciable relative sensitivity S_r_ and the signal-to-noise ratio from both emission bands.^71^

#### 3.5.1 Thermometric Performance of **Crddpd** in Different Environments

For the calculation of the thermometric figures of merit S_r_, LIR, and δT for dual emissive **Crddpd** in air- and Ar-saturated solution and encapsulated in l-arginine- and Stöber-type SiO_2_ NPs in air-saturated dispersion, shown in Figure 7 (top), data derived from triplicate measurements of the first heating cycle (Figure 6) were used because the variability between the consecutive heating cycles was larger than the replicate-to-replicate variation within a single cycle. This also enabled a direct comparison with the values subsequently derived for **Ruphenpy**. First, each PL spectrum of the Cr^III^ complex was normalized at 736 nm, the maximum of the high-energy PL band and then divided into two spectral windows *I*_1_(690 – 744 nm) and *I*_2_(745–840 nm). The LIR data were then calculated as the ratio of the subsequently integrated areas of *I*_1_ and *I*_2_. S_r_, and δT were calculated as detailed in the Supporting Information. LIR, S_r_, and δT values calculated from data obtained for all three consecutive heating–cooling cycles are provided in the Supporting Information (Figure S55).

**Figure 7:**
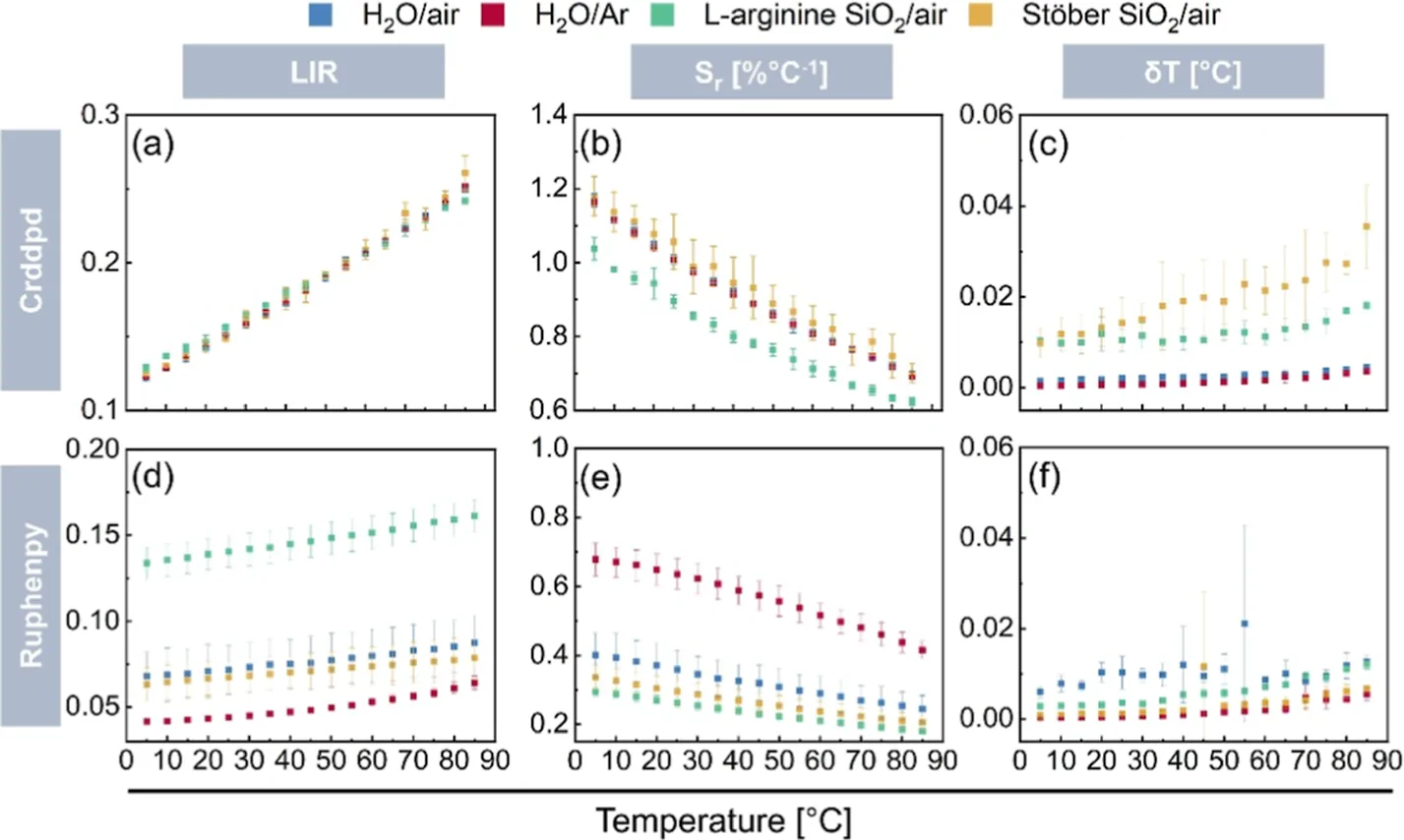
Top: temperature dependence of (a) the PL intensity ratio (LIR), (b) the relative sensitivity (S_r_), and (c) the thermal resolution (temperature uncertainty, δT) of **Crddpd** in air- and Ar-saturated aqueous solution and encapsulated in l-arginine- and Stöber-type SiO_2_ NPs dispersed in air-saturated water. The LIR, S_r_, and δT values were calculated from triplicate measurements of the first heating cycle (Figure 6). Bottom: temperature dependence of (d) LIR, (e) S_r_, and (f) δT of **Ruphenpy** in air- and Ar-saturated aqueous solution and encapsulated in l-arginine and Stöber-type SiO_2_ NPs dispersed in air-saturated water. The LIR, S_r_, and δT values were calculated from triplicate measurements of the first heating cycle (Figure 4).

As shown in Figure 7 (top), dual emissive **Crddpd** exhibits nearly identical LIR values in all environments, with only slightly increased error bars observed for the Stöber system at temperatures exceeding 30 °C. This indicates that the ratiometric calibration of **Crddpd** is largely independent of O_2_, because both PL bands are similarly affected by O_2_ quenching. However, O_2_ quenching still reduces the absolute PL intensity. This can influence the practical thermometric performance through a lower signal-to-noise ratio, reduced sensitivity, and increased uncertainty of PL-based temperature measurements, even if the LIR calibration remains nearly unchanged. The temperature dependence of the LIR follows the expected Boltzmann-type behavior for two thermally coupled emissive states (see Supporting Information, Figures S40–S44). For **Crddpd**, the S_r_ values in aqueous solution in air and under Ar atmosphere and embedded in Stöber-type SiO_2_ NPs in air closely match, reaching values of about 1.2% °C^–1^ at 5 °C and decreasing to about 0.7% °C^–1^ at 85 °C. For **Crddpd** encapsulated in l-arginine–type SiO_2_ NPs, the S_r_ curve decreases from about 1.0% °C^–1^ at 5 °C to about 0.6% °C^–1^ at 85 °C, respectively. These values are in the lower-to-mid range reported for Cr^III^ and Ru^II^ luminescence thermometers at ambient or higher temperatures^72^ with the slight differences in S_r_ (S_r_ = Δ*E*/(*k*_B_ *T* ^2^) originating from the slightly different local crystal fields in the different environments of **Crddpd**. The reduction in S_r_ of **Crddpd** in the l-arginine type silica network could possibly point to changes in the vibrational freedom in this matrix. For **Crddpd** in air- and Ar-saturated aqueous solutions, the δT values remain very small over the entire temperature range assessed. The δT values of **Crddpd** embedded in SiO_2_ NPs are slightly higher, especially for Stöber-type SiO_2_ NPs, and increase with increasing temperature. Overall nonencapsulated and SiO_2_ NP-encapsulated **Crddpd** perform very well as luminescent molecular or nanothermometers. Temperature-dependent time-resolved PL studies (Supporting Information, Section S5.2) reveal that especially **Crddpd** in l-arginine–type SiO_2_ NPs shows the smallest response to thermal quenching in the temperature range, together with an excellent reproducibility (Supporting Information, Figure S50).

A comparison of the different encapsulated **Crddpd**-based PL thermometers assessed in this study to established solid-state PL Boltzmann thermometers relying on Cr^III^ in a strong ligand field, such as ruby (α-Al_2_O_3_/Cr^3+^) or AlB_4_O_6_N/Cr^3+^,^3,64^ reveals a similar performance of all these systems in terms of S_r_ in the temperature range of 0 to 85 °C. This reflects the similar energy separation between the two lowest-energy doublet states ^2^T_1_(^2^G) and ^2^E­(^2^G) of Cr^III^ in these systems. Ruby and AlB_4_O_6_N/Cr^3+^ show a good brightness, which is relevant for the overall achievable (statistical) precision of a luminescent Boltzmann thermometer, and only a limited influence of thermal quenching over a very wide temperature range as reflected by the long radiative decay times τ_rad_ of the ^2^E­(^2^G) state of Cr^III^ of about 6 and 30 ms, respectively. This is beneficial for a wide dynamic working range.^64,71,73^ Moreover, with respect to the time scale of thermalization, nonradiative coupling between the ^2^T_1_(^2^G) and ^2^E­(^2^G) state of Cr^III^ is very fast^64,74,75^ and outperforms classical Boltzmann thermometers relying on the green-emitting excited levels of Er^III^.^76^ However, the much smaller size of molecular rubies and derived NP systems is advantageous for temperature sensing, especially in the life sciences. In addition, the more covalent nature of the Cr–N bonds in the complexes strongly reduces interelectronic repulsion and shifts the spin-forbidden narrow-line PL in complexes such as **Crddpd** to longer wavelengths compared to the R line PL in ruby and AlB_4_O_6_N/Cr^3+^ (λ_em_ ≈ 694 or 683 nm, respectively).^77^

#### 3.5.2 Thermometric Performance of **Ruphenpy** in Different Environments

Subsequently, we determined the parameters LIR, S_r_, and δT from the measured temperature-dependent PL data of free and SiO_2_ NP-encapsulated **Ruphenpy** measured in air-saturated dispersion using the data from the first heating cycle (Figure 7, bottom row). The data derived from all three consecutive heating cycles are shown in the SI in Figure S55. We utilized a pseudoratiometric spectral-window analysis for the single-band emitter **Ruphenpy** following the example of other luminophores with noncoupled energy levels (single-band ratiometric approach) such as Ag_2_S quantum dots that even exhibit temperature-induced spectral shifts and changes in spectral bandwidth.^68,78^ First, the temperature-dependent PL spectra were normalized to the integrated area (integration limits were set from 500 to 800 nm, wavelength scale was used for integration) of each PL spectrum to enable consideration of changes in spectral shape. Next, the wavelength at which the normalized PL spectra cross was determined (a near-isothermal crossing region), and the PL spectra were normalized to the PL intensity at this near-isothermal crossing region average point, which served as the boundary for defining two spectral regions (*I*_1_ and *I*_2_) for this single-emissive system (Supporting Information, Figure S39). LIR values were then calculated as *I*_1_/*I*_2_, while S_r_ and δT values were determined according to the equations given in the Supporting Information (Section S5). As displayed in Figure 7 (bottom), the LIR and S_r_ data derived for the **Ruphenpy** systems reveal the same trends independent of O_2_ PL quenching or O_2_ protection, yet the values differ. The highest LIR values were obtained for **Ruphenpy@ARGSiO**_**2**_ in air-saturated dispersion, and the lowest values for the nonencapsulated dyad in Ar-saturated aqueous solution. The LIR values derived for the free dyad and **Ruphenpy@STOSiO**_**2**_ in air-saturated water, where O_2_ PL quenching is feasible, are nearly identical within the experimental uncertainty. The highest S_r_ values are found for the dyad in water/Ar with S_r_ reaching about 0.7% °C^1–^ at 5 °C and 0.4% °C^1–^ at 85 °C. These results agree well with the performance of other previously reported Ru^II^ thermometers such as [Ru­(dpp)_3_]^2+^ and [Ru­(bpy)_3_]^2+^ in solution (dpp = 4,7-diphenyl-1,10-phenanthroline).^79–81^ For the other three **Ruphenpy** NP systems, the S_r_ values are very similar, and there is no significant difference between the S_r_ values obtained for the l-arginine- and Stöber-type SiO_2_ NPs. This is tentatively ascribed to similar interactions between **Ruphenpy** and the silica matrix for both SiO_2_ NPs. Interestingly, the comparison of the temperature dependence of S_r_ for **Ruphen@ARGSiO**_**2**_ and **Ruphenpy@ARGSiO**_**2**_ (Supporting Information, Figure S56) reveals consistently higher S_r_ values for **Ruphen@ARGSiO**_**2.**_ The δT values obtained for **Ruphenpy** in different environments closely match, except for water/air, where larger uncertainties were observed at 40 °C, 45 °C, and 55 °C. This is tentatively ascribed to O_2_ escape from solution.^79^

### 3.6 Stability and Repeatability Studies with **Ruphenpy** and **Crddpd**

Finally, we assessed the repeatability of optical temperature measurements with **Ruphenpy** and **Crddpd** in different environments of varying O_2_ quenching protection, utilizing the data displayed in Figures 4 and 6 for different temperatures and three consecutive heating–cooling cycles. The results, obtained for **Crddpd** (a–d) and **Ruphenpy** (e–h) in different environments from the normalized integrated PL at 5 °C (red), 60 °C (blue), and 85 °C (green), are displayed in Figure 8. The blue, green, and red symbols refer to the integrated PL intensity measured at the same temperature in each cycle, while the values given in percentage provide measures for the consistency of these repeated measurements. A value close to 100% implies that the signal is highly reproducible from cycle to cycle, whereas lower percentages indicate larger fluctuations and therefore poorer stability/reversibility under the respective conditions, in our cases due to the temperature-dependent solubility of O_2_ and its removal within the repeated measurement cycles. Using this description, with values of about 95–99%, the l-arginine SiO_2_ encapsulated samples exhibit the best repeatability for both luminophores, whereas the nonencapsulated luminophores in H_2_O/air show the largest variability, particularly at 5 °C. O_2_ removal by flushing with Ar or encapsulation in Stöber SiO_2_ improves the performance of both luminescence thermometers, especially for **Ruphenpy**. Overall, optical temperature measurements with O_2_-sensitive luminophores can be performed with high repeatability only for efficient O_2_ shielding. In addition, the data shown in Figure 8 confirm the high thermal stability of our luminescence thermometers.

**Figure 8:**
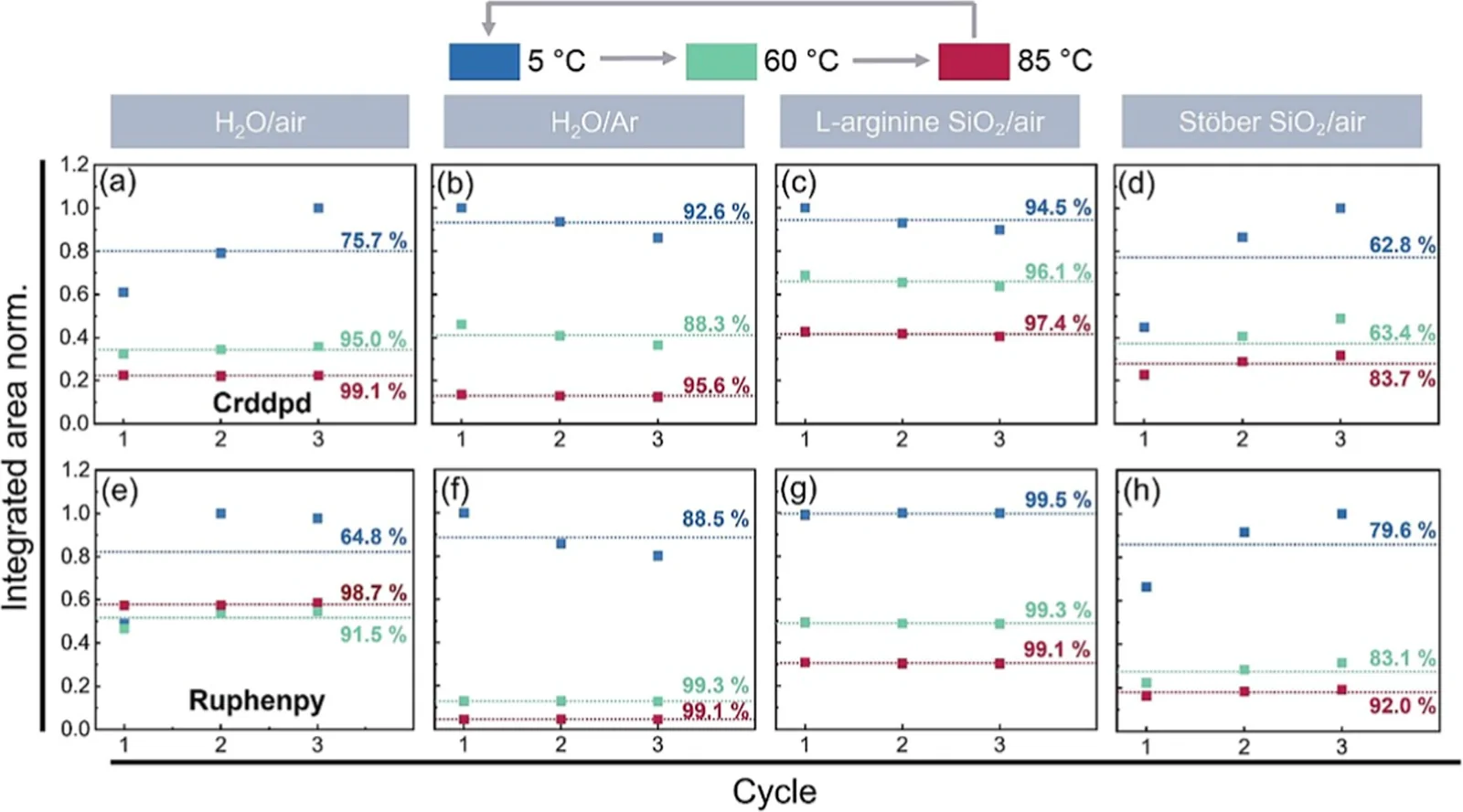
Cycle-to-cycle changes of the normalized integral emission at 5 °C, 60 °C, and 85 °C derived for **Crddpd** (a–d) and **Ruphenpy** (e–h) in different environments: H_2_O/air, H_2_O/Ar, l-arginine–mediated SiO_2_/air, and Stöber SiO_2_/air. These data provide estimates for the good stability and reversibility of these luminescence thermometers.

Also, we performed a similar analysis as well as aging studies, representatively for **Crddpd** using time-resolved PL measurements (Supporting Information, Section S5.2). The results derived from steady state and time-resolved measurements show very similar temperature-dependent trends (Figures 6d,h,i,p and S50a–d) despite the different acquisition times for PL spectra and PL decay kinetics. A reversibility test performed representatively for **Crddpd@STOSiO**_**2**_ (three cycles, reoxygenation with pressed air, followed by an additional heating cycle) confirmed that the PL decay times resulting after reintroducing O_2_ closely match the values measured in the first heating cycle (Supporting Information, Figure S51). Representative aging studies of **Crddpd@STOSiO**_**2**_ stored in air-saturated Milli-Q water (pH 6.9), monitored by time-resolved PL measurements, revealed a steady increase in the average PL lifetime and integral PL decay curve under atmospheric conditions already within 1 week (Supporting Information, Figure S52). This finding agrees well with the results of our previous studies with Cr^III^ compounds in different silica networks.^25^ This behavior is attributed to ongoing silica aging, most likely caused by the condensation of surface silanol groups, which can change the silica network/surface chemistry and thereby reduce the O_2_ permeability of the Stöber- type SiO_2_ NPs. This underlines the importance of silica surface chemistry for efficient O_2_ shielding. In contrast, **Crddpd@ARGSiO**_**2**_ retains a stable oxygen-shielded PL behavior over at least 28 days under the applied storage and measurement conditions (Supporting Information, Figure S53).

## 4 Conclusion and Outlook

Incorporation of the representatively chosen Ru^II^ complex **Ruphen** and the long-lived Ru^II^ dyad **Ruphenpy** into silica nanoparticles (SiO_2_ NPs) by the l-arginine mediated method (**Ruphen@ARGSiO**_**2**_ and **Ruphenpy@ARGSiO**_**2**_) and the classical ammonia-catalyzed Stöber method (**Ruphenpy@STOSiO**_**2**_) led to monodisperse **Ruphen**- and **Ruphenpy**-stained SiO_2_ NPs with sizes of about 25 nm. The resulting PL properties in aqueous dispersion in air, observed for the former and latter type of silica network, verify the previously reported complete O_2_ shielding of O_2_-responsive luminescent Cr^III^ complexes by l-arginine- type silica networks. The observed partial protection from PL quenching by O_2_ in Stöber-type SiO_2_ NPs agrees well with the results reported by other research groups for Ru^II^ compounds^70,82^ and by us for two molecular rubies.^25^ Remarkable differences in O_2_ shielding efficiency of the l-arginine and Stöber-type silica networks underline the influence of the sol–gel synthesis route on the O_2_ permeability of the resulting silica materials on silica matrix O_2_ protection efficiency, which is attributed to different densities of the resulting SiO_2_ NPs, together with a slightly different surface chemistry as supported by the different zeta potentials and stability studies with Stöber-type SiO_2_ NPs. The broad applicability of our simple l-arginine-type silica shielding concept for the protection of O_2_- sensitive luminophores from collisional PL quenching by O_2_ can pave the road for many new applications of O_2_- sensitive compounds in air in bioimaging, sensing, and energy conversion studies as well as in multiplexed or time-gated detection schemes and spectral shifting, that all require high PL efficiency. In addition, this encapsulation strategy can enhance the stability of photolabile compounds such as Ru^II^ complexes as demonstrated for **Ruphen** and **Ruphenpy**.

Temperature-dependent PL measurements of **Crddpd** and the two Ru^II^ complexes and the luminophores encapsulated in l-arginine- and Stöber-type SiO_2_ NPs in air- and argon-saturated solutions demonstrate the almost completely preserved temperature sensitivity of all luminophores in both silica networks. In addition, our studies demonstrate the importance of complete O_2_ protection for reliable and comparable temperature and analyte sensing with O_2_-sensitive single wavelength emissive luminophores in air utilizing changes in PL intensity and PL decay kinetics or lifetime. For Boltzmann-type dual emissive Cr^III^ compounds, the influence of O_2_ concentration on the reliability of optical temperature sensing is negligible, however, the signal intensities and hence the sensitivity of the unshielded Cr^III^ PL thermometer is slightly reduced. These findings highlight the importance of determining the matrix O_2_ shielding efficiency to identify matrices and synthetic procedures, which provide optimum PL properties and brightness of O_2_-sensitive luminophores, and to eliminate interferences from temperature-dependent O_2_ solubility and to improve the comparability of different sensors with non-Boltzmann- type PL characteristics.

Overall, our results also underline the strong potential of molecular rubies such as **Crddpd** and encapsulated complexes such as **Crddpd** in l-arginine–type SiO_2_ NPs or other nanoparticles for increasingly popular thermometry applications in the life sciences and bioimaging in the physiological relevant temperature range due to their optimally suited energy gap of Δ*E* ≈ 600 cm^–1^ and the Cr^III^ PL in the first biological window (650 - 950 nm). A disadvantage is, however, the excitability of the Cr^III^ complexes at relatively short wavelengths ≤450 nm, resulting in a limited penetration depth of the exciting light, and favoring contributions from scattering and autofluorescence. Such signal distortions can be overcome by PL lifetime measurements and the usage of the demonstrated integral decay curve approach for temperature sensing. Important for temperature sensing is, however, to control quenching pathways of both spin-flip excited states of Cr^III^, which could otherwise affect the accessible dynamic working range of this type of luminescent thermometer. For PL lifetime sensing with matrix-encapsulated luminophores, which often reveal complex PL decay kinetics, normalized integral PL decay curves could be exploited. This simple approach is independent of luminophore concentration and fluctuations of the excitation power densities contrary to PL intensity measurements and additionally avoids time-consuming and error-prone data fitting procedures of PL decay curves.

In the future, we will probe the O_2_ permeability of silica networks prepared with different methods under different conditions with our Cr^III^ compounds to gain an in-depth understanding of the relevant synthetic parameters controlling the O_2_ permeability of these networks. This will provide the basis to correlate silica network O_2_ permeability and preparation method. Thereby, we will exploit the tunability of the dispersibility of these Cr^III^ complexes via the choice of the counteranion without affecting their optical properties. In addition, we will perform systematic long-term aging studies of Ru^II^-loaded l-arginine and Stöber SiO_2_ NPs to support future sensor applications and assess the possibility of PL lifetime tuning with the aid of differently synthesized silica networks for O_2_-sensitive luminophores such as Cr^III^ and Ru^II^ complexes. Intended applications are the design of a set of nanoscale lifetime labels with matching absorption and emission spectra for lifetime multiplexing and encoding in the visible and NIR with single wavelength excitation.

## Supplementary Material



## Notes

All data needed to support the conclusions in the article are presented in the manuscript and the Supporting Information (PDF)
