# Supplementary material for: Oxygen-Unaffected Temperature Sensing in Air-Saturated Aqueous Dispersion with Oxygen-Sensitive Luminescent Metal Complexes Encapsulated in l‑Arginine-Type Silica Networks
Source: ACS Meas Sci Au. 2026 Aug 10;6(5):1597–613. doi: 10.1021/acsmeasuresciau.6c00124 (PMC13693274; doi:10.1021/acsmeasuresciau.6c00124)
Supplement: Supplementary file 1 [file tg6c00124_si_001.pdf]

Supporting information

Oxygen-unaffected temperature sensing in air-saturated aqueous dispersion with oxygen-sensitive luminescent metal complexes encapsulated in L-arginine-type silica networks

Viktoriia Osipova[1,2], Christian Homann[1], Arne C. Weber[3], Lingcong Ge[4], Isabella Tavernaro[1], Elena Meßmer[3], Katja Heinze[3], Michael R. Reithofer[4], Christoph Kerzig[3] and Ute Resch-Genger[1,*]

[1]Federal Institute for Materials Research and Testing (BAM), Division 1.2 Biophotonics, Richard-Willstaetter-Str. 11, 12489 Berlin, Germany.

[2]Free University Berlin, Institute for Chemistry and Biochemistry, Takustr. 3, 14195 Berlin, Germany.

[3]Johannes Gutenberg University Mainz, Department of Chemistry, Duesbergweg 10-14, 55128 Mainz, Germany.

[4]Institute of Inorganic Chemistry, Faculty of Chemistry, University of Vienna, Waehringer Str. 42, 1090 Vienna, Austria.

Corresponding Author

*E-mail: ute.resch@bam.de

Contents

S1. Syntheses of Cr^{III} complex - $[Cr(ddpd)_2](BF_4)_3$ (Crddpd) 3

S2. Syntheses of Ru^{II} complex and Ru^{II}-dyad molecule - $[Ru(phen)_3]Cl_2$ (Ruphen) and $[Ru(phen)_2(phenpy)]Cl_2$ (Ruphenpy) 3

S3. Particle synthesis and characterization 4

S3.1. Syntheses of unstained (US-SiO_2 NPs) and Crddpd- and Ruphenpy-stained SiO_2 NPs using the Stöber approach 4

S3.2. Syntheses of unstained (US-ARGSiO_2 NPs) and Crddpd-, Ruphen-, and Ruphenpy-stained SiO_2 NPs using the L-arginine approach 5

S3.3. Syntheses of core@shell particles by the L-arginine approach 7

S3.4. Increasing Ruphen and Ruphenpy loading concentration in ARGSiO_2 to enable the line-scan detection of Ru^{II} 9

S3.5. Dynamic light scattering (DLS) 9

S3.6. Zeta potential (ζ-potential) measurements 13

S3.7. Electron microscopy (EM) measurements 16

S3.8. Inductively coupled plasma mass spectrometry (ICP-MS) 25

S4. Optical properties 29

S4.1. Methods 29

S4.2 Oxygen sensitivity of Ruphenpy-loaded L-arginine SiO_2 core@shell nanoparticles 35

S4.3 Oxygen sensitivity of Ruphen-loaded L-arginine SiO_2 nanoparticles 36

S4.4 Photostability studies 36

S.4.41 Experimental setup and sample preparation 36

S5. Temperature sensing studies 40

S5.1 Crddpd: luminescence intensity-based temperature sensing 43

S5.2 Crddpd: lifetime-based temperature sensing 46

S5.3. Normalized integrated-emission-area analysis of Crddpd and Ruphenpy during temperature cycling 56

S5.4. Ruphenpy and Ruphen: luminescence intensity-based temperature sensing 58

S5.4 Ruphenpy: lifetime-based temperature sensing 60

References 62

S1. Syntheses of Cr^{III} complex - $[Cr(ddpd)_2](BF_4)_3$ (Crddpd)

The syntheses of the Cr^{III} complex $[Cr(ddpd)_2](BF_4)_3$ (**Crddpd**) was performed according to reported literature procedures [1, 2].

S2. Syntheses of Ru^{II} complex and Ru^{II}-dyad molecule - $[Ru(phen)_3]Cl_2$ (Ruphen) and $[Ru(phen)_2(phenpy)]Cl_2$ (Ruphenpy)

Synthesis of 5-(pyren-1-yl)-1,10-phenanthroline (phenpy)

The ligand phenpy was prepared from 5-bromo-1,10-phenanthroline (1.03 g, 3.96 mmol) and pyrene-1-ylboronic acid (1.08 g, 4.39 mmol) in a two-phase mixture of toluene (160 mL), ethanol (52 mL), and aqueous $Ba(OH)_2$ solution (208 mL), following the procedures previously reported by Castellano et al. [3] and Kerzig et al. [4]. The reaction mixture was refluxed under an argon atmosphere for two days. After extraction of the crude product with toluene and evaporation of the solvent, phenpy was purified by recrystallization from toluene, yielding 1.26 g (3.31 mmol, 83%). The ^1H NMR spectrum is in agreement with that reported in the literature [3, 4].

^1H NMR (400 MHz, $CDCl_3$) δ = 9.32 (dd, J = 4.4, 1.7 Hz, 1H), 9.24 (dd, J = 4.3, 1.7 Hz, 1H), 8.38 – 8.30 (m, 2H), 8.27 (dd, J = 7.7, 1.2 Hz, 1H), 8.22 – 8.14 (m, 3H), 8.11 – 7.89 (m, 4H), 7.84 – 7.71 (m, 2H), 7.62 (d, J = 9.2 Hz, 1H), 7.44 (dd, J = 8.3, 4.3 Hz, 1H) ppm.

Synthesis of Ruphenpy as both PF_6 and Cl salt

The sensitizer **Ruphenpy** was prepared from dichlorobis(1,10-phenanthroline)ruthenium(II) (352 mg, 0.62 mmol) and phenpy (288 mg, 0.76 mmol) in methanol (300 mL), following the procedures previously reported by Castellano et al.[3] and Kerzig et al. [4]. The reaction mixture was refluxed under an argon atmosphere for 4 h in the absence of light. After cooling to room temperature, water (180 mL) was added, and the PF_6 salt (530 mg, 75%) was precipitated by dropwise addition of 10 mL of saturated NH_4PF_6(aq).

The PF_6 salt of the Ru^{II} complex was purified by filtration through silica using methanol to remove the uncoordinated phenpy ligand. To elute the purified complex from the silica, dichloromethane was gradually added to methanol, and the solvent mixture was then treated with a small amount of NH_4PF_6 until a 50:1 DCM:MeOH ratio was reached.

To improve water solubility, a portion of the PF_6 salt was converted to the chloride salt via anion exchange using Amberlite IRA-410 in ethanol. After removal of the solvents under

reduced pressure, the water-soluble chloride salt of **Ruphenpy** was obtained as a red solid. The ^{1}H NMR spectrum was in agreement with that reported in the literature [4].

Ruphenpy (Cl salt)

^{1}H NMR (400 MHz, CD_3CN) δ = 8.77 – 8.60 (m, 5H), 8.51 – 8.17 (m, 12H), 8.17 – 8.03 (m, 7H), 8.02 – 7.86 (m, 2H), 7.86 – 7.78 (m, 1H), 7.77 – 7.61 (m, 4H), 7.43 – 7.36 (m, 1H) ppm.

S3. Particle synthesis and characterization

Silica nanoparticles (SiO_2 NPs) prepared by two-sol gel synthesis routes were selected as matrices for embedding and protecting **Ruphen**, **Ruphenpy** (both as chloride salts) and **Crddpd** from oxygen quenching. SiO_2 NPs offer well-established synthetic protocols that enable tuning of the particle size from a few nanometers to several micrometers with high monodispersity, along with high biocompatibility, excellent chemical inertness, good colloidal stability over a broad pH range (≈3–10), and straightforward surface functionalization that allows, for example, subsequent bioconjugation reactions. In this study, two different synthetic routes were employed for the preparation of SiO_2 NPs (Figure 1(bottom row), i.e., the widely used ammonia-catalyzed Stöber process, which yields SiO_2 NPs with diameters from about 50 nm up to several micrometers [5], and the less common L-arginine-mediated method. The latter is based on the controlled hydrolysis of the silicon precursor in a biphasic cyclohexane/water system and affords monodisperse, amorphous, non-porous SiO_2 NPs with sizes ranging from approximately 5 nm to several hundred nanometers [6, 7].

The size, morphology, particle dispersity, surface charge, and chemical composition of the SiO_2 NPs loaded with the respective luminescent complexes were characterised by dynamic light scattering (DLS), ζ-potential measurements, several electron microscopy (EM) techniques, and inductively coupled plasma mass spectrometry (ICP-MS). In addition, unloaded reference SiO_2 NPs (**US-ARGSiO$_2$ NPs/US-STOSiO$_2$ NPs**) were synthesised. For a subset of samples, an additional silica shell was grown in a subsequent step (Figure S3).

S3.1. Syntheses of unstained (US-SiO$_2$ NPs) and Crddpd- and Ruphenpy-stained SiO$_2$ NPs using the Stöber approach

Unstained SiO$_2$ NPs (25 nm US-STOSiO$_2$ NP) made by the Stöber approach [6]

A reaction mixture containing 0.41 mL of MilliQ-water, 23.57 mL of absolute ethanol (Chemsolute, Th. Geyer, Germany), and 0.50 mL of ammonia (25%, abcr GmbH, Germany) was prepared and stirred at room temperature (r.t., T = 23 ± 2 °C). 0.55 mL (2.5 mmol) of

tetraethyl orthosilicate (TEOS, Sigma Aldrich, Germany) was added. The reaction mixture was stirred for an additional 2 d at r.t., before the particle dispersion was purified via dialysis against water for 24 h (4 L, water exchange after 1 h, 2 h and 4 h) using a regenerated cellulose membrane with a molecular weight cut-off (MWCO) of 10 kDa (Nadir®, Carl Roth GmbH, Germany).

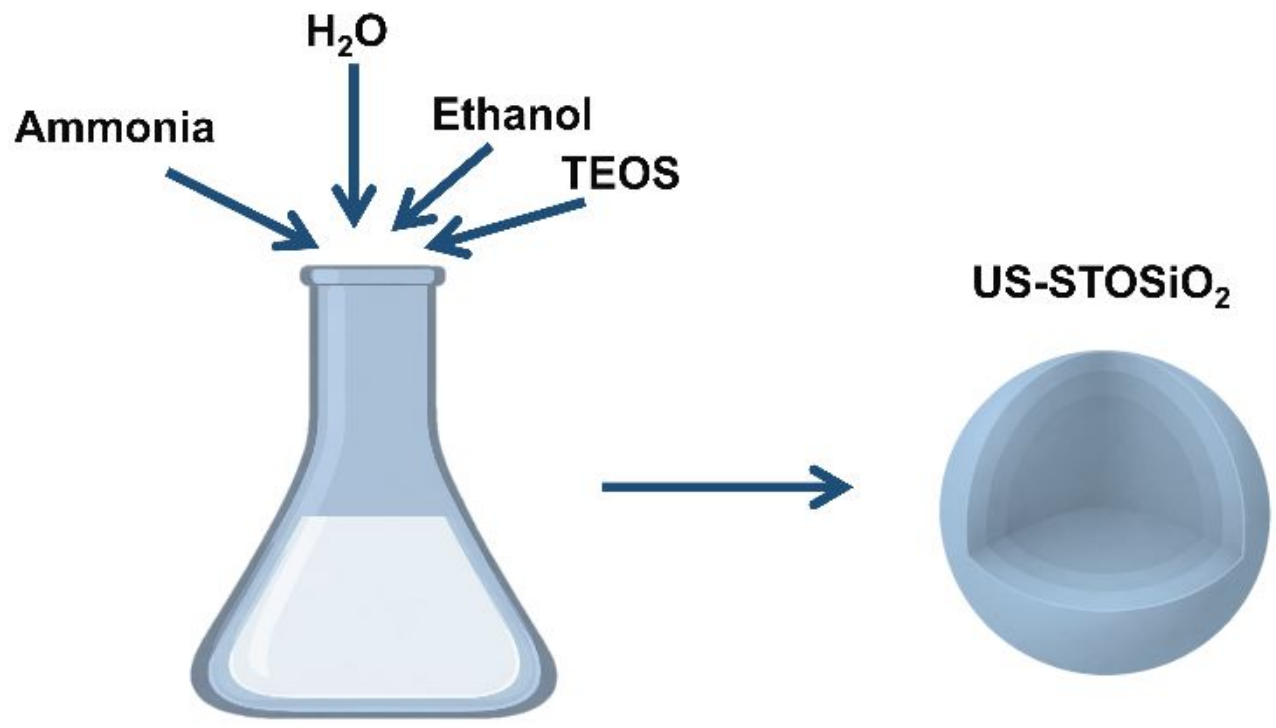

Figure S1. Reaction scheme of the syntheses of unstained SiO_2 NPs using the Stöber approach – **US-STOSiO$_2$**: TEOS (0.55 mL); ammonia (25%, 0.50 mL), EtOH/H_2O (57.5:1), 2 d; r.t.;

Crddpd-stained SiO_2 NPs (Crddpd@STOSiO$_2$) made by the Stöber approach [6]

For the preparation of Cr^{III} complex-loaded SiO_2 NPs, the above-described procedure was slightly modified by adding 100 µL (0.1 µmol) of **Crddpd** in acetonitrile (ACN, 99.9%, Sigma-Aldrich, Germany) directly before the addition of the corresponding amount of TEOS (0.55 mL, 2.5 mmol). A schematic representation of the synthesis procedure is shown in Figure 1 (bottom row, right part).

Ruphenpy-stained SiO_2 NPs (Ruphenpy@STOSiO$_2$) Made by the Stöber Approach [6]

For the preparation of **Ruphenpy**-loaded SiO_2 NPs, the above-described procedure was slightly modified by adding 100 µL (0.1 µmol) of **Ruphenpy** in *N,N*-dimethylformamide (DMF, 99.8%, Sigma-Aldrich, Germany) directly before the addition of the corresponding amount of TEOS (0.55 mL, 2.5 mmol). A schematic representation of the synthesis procedure is shown in Figure 1 (bottom row, right part).

S3.2. Syntheses of unstained (US-ARGSiO$_2$ NPs) and Crddpd-, Ruphen-, and Ruphenpy-stained SiO_2 NPs using the L-arginine approach

Unstained SiO_2 NPs (25 nm US-ARGSiO$_2$ NPs) [7]

To synthesize 25 nm unstained SiO_2 NPs, a previously described protocol was used for the controlled hydrolysis of the silane precursor in a biphasic MilliQ-water/cyclohexane system

catalyzed by L-arginine [6]. Therefore, 91 mg (0.52 mmol) of L-arginine (98.5%, Carl Roth GmbH, Germany) were dissolved in 69 mL of MilliQ-water, before 4.5 mL of cyclohexane (Sigma Aldrich, Germany) was added as a top layer. The reaction mixture was heated to 65 °C under stirring. After addition of 5.5 mL (24.63 mmol) TEOS, the reaction mixture was stirred under these conditions for an additional 20 h. Then, the reaction was stopped, cooled down to r.t., and the two phases were separated. The aqueous phase was subsequently purified by dialysis against water for 24 h (4 L, water exchange after 1 h, 2 h and 4 h, Nadir®, MWCO: 10 kDa).

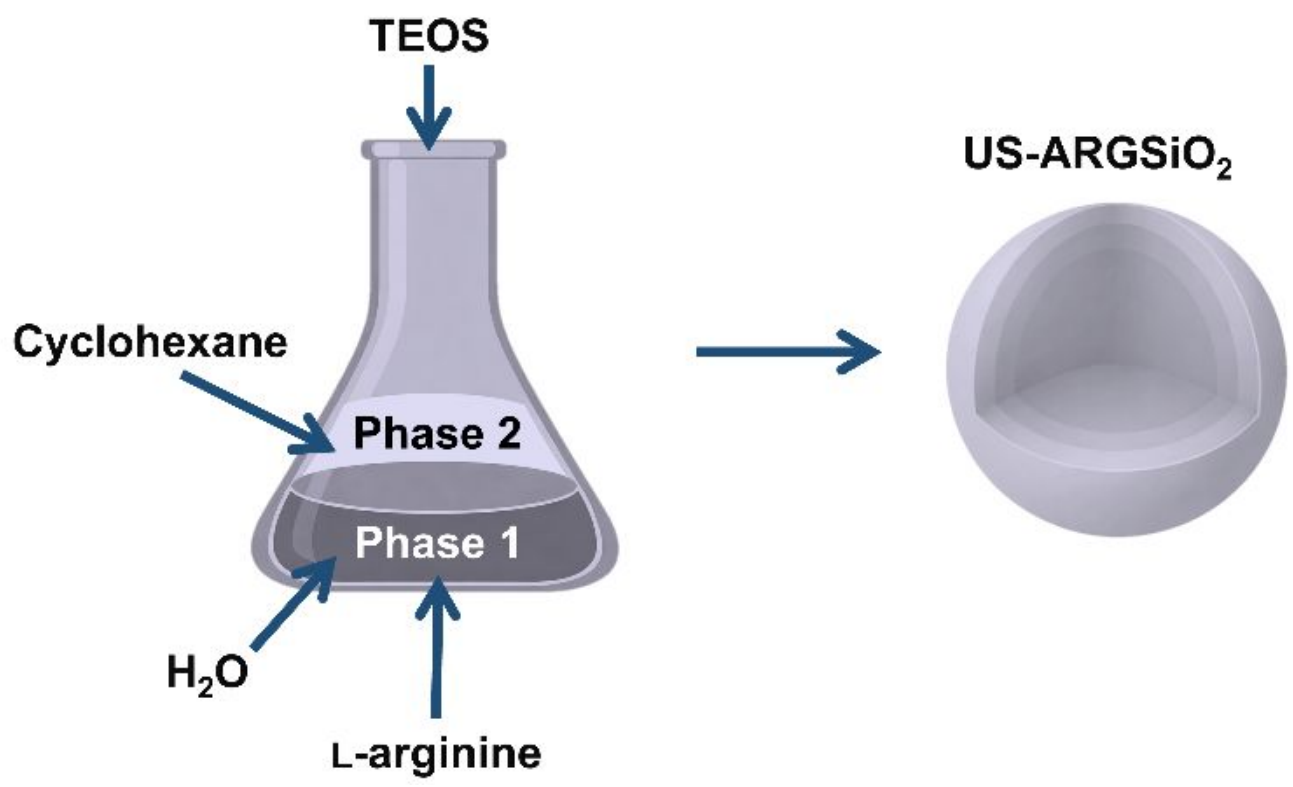

Figure S2. Reaction scheme of the syntheses of the unstained SiO_2 NPs using the L-arginine approach – **US-ARGSiO$_2$**: L-arginine (0.52 mmol); H_2O/cyclohexane (15.3:1); TEOS (24.63 mmol); 65 °C.

Crddpd-stained SiO_2 NPs (Crddpd@ARGSiO$_2$) made by the L-arginine approach [6]

For the preparation of Crddpd-loaded SiO_2 NPs, the above-described procedure was slightly modified by adding 100 µL (0.1 µmol) of **Crddpd** in acetonitrile (ACN, 99.9%, Sigma-Aldrich, Germany) 20 min. after addition of the corresponding amount of TEOS (0.55 mL, 2.5 mmol). A schematic representation of the synthesis procedure is shown in Figure 1 (bottom row, left part).

Ruphen-stained SiO_2 NPs (Ruphen@ARGSiO$_2$) made by the L-arginine approach [6]

For the preparation of **Ruphen**-loaded SiO_2 NPs, the above-described procedure was slightly modified by adding 100 µL (0.1 µmol) of **Ruphen** in *N,N*-dimethylformamide (DMF, 99.8%, Sigma-Aldrich, Germany) 20 min. after the addition of the corresponding amount of TEOS (0.55 mL, 2.5 mmol). A schematic representation of the synthesis procedure is shown in Figure 1 (bottom row, left part).

Ruphenpy-stained SiO_2 NPs (Ruphenpy@ARGSiO$_2$) made by the L-arginine approach [6]

For the preparation of **Ruphenpy**-loaded SiO_2 NPs, the above-described procedure was slightly modified by adding 100 µL (0.1 µmol) of **Ruphenpy** in DMF, 20 min after the addition of the corresponding amount of TEOS (0.55 mL, 2.5 mmol) and stirring of the reaction mixture. A schematic representation of the synthesis procedure is shown in Figure 1 (lower left part).

S3.3. Syntheses of core@shell particles by the L-arginine approach

Unstained core@shell SiO_2 NPs (40 nm US-ARGSiO$_2$) [6]

The growth of a SiO_2 shell around the previously synthesized 25 nm particles **US-ARGSiO$_2$ NPs** was performed by dissolving 14 mg (0.08 mmol) of L-arginine in an aqueous mixture of MilliQ-water (36 mL) and a previously prepared suspension of **US-ARGSiO$_2$ NPs** (10 mL). After addition of 5 mL of cyclohexane, the biphasic mixture was heated to 65 °C under stirring, before 3.52 mL (15.8 mmol) of TEOS was added. The reaction mixture was stirred for further 20 h, followed by purification as described above. A schematic representation of the synthesis procedure is shown in Figure S3.

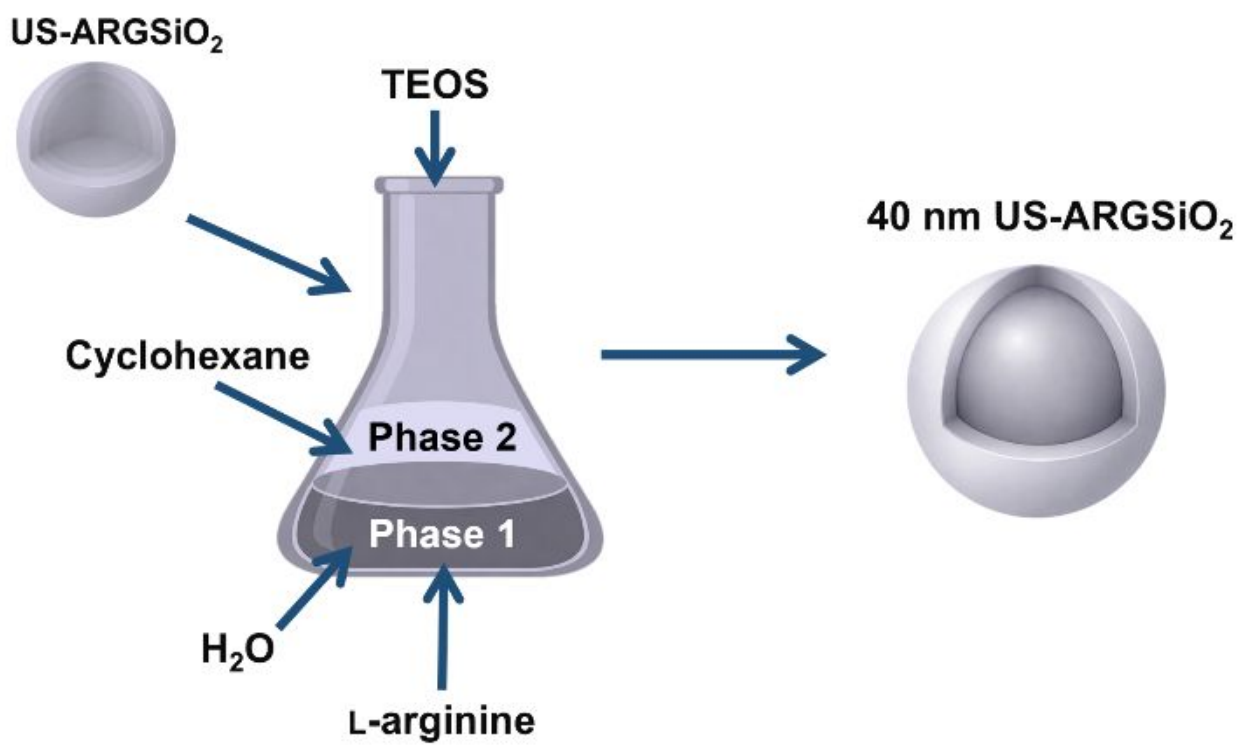

Figure S3. Reaction schemes of the shelling of unstained SiO_2 NPs using the L-arginine approach. The synthesis of core/shell SiO_2 NPs is shown for 40 nm **US-ARGSiO$_2$** NPs constituting of an unstained SiO_2 core (**US-ARGSiO$_2$**, Synthesis is shown in Figure S2) and an unstained shell: TEOS (15.8 mmol), L-arginine (0.08 mmol), H_2O/cyclohexane (9.2:1).

Unstained core SiO_2 NPs shelled with a Ruphenpy-stained SiO_2 Shell (US-ARGSiO$_2$)@Ruphenpy@ARGSiO$_2$)

A similar approach was chosen for all particles synthesized by the L-arginine approach. After all parameters were set and the particle formation was induced as described for the unstained SiO_2 particles (**US-ARGSiO$_2$** NPs), 100 µL (0.1 µmol) of the **Ruphenpy**, dissolved in DMF, was added 20 min after TEOS addition. The reaction mixture was heated for an additional 20 h, before the particle dispersion was purified by dialysis as described before. A schematic presentation of the synthesis procedures is shown in Figure S4.

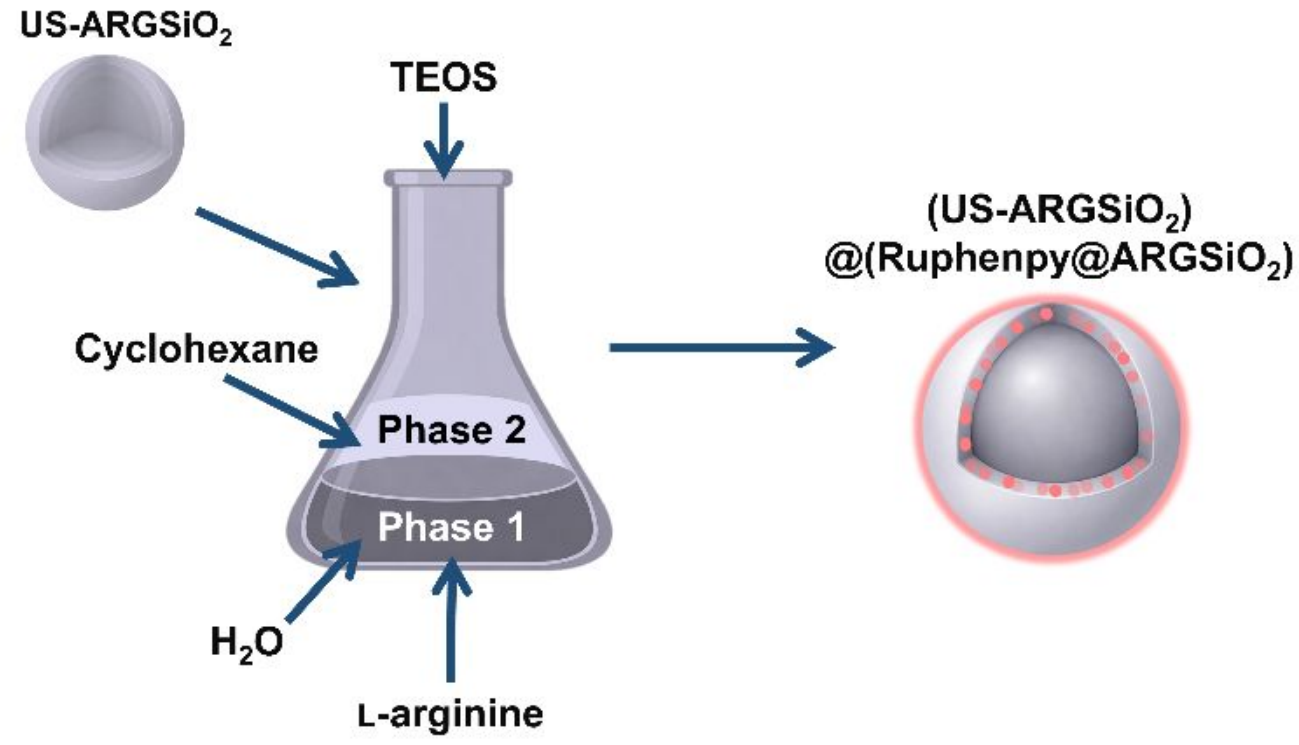

Figure S4. Reaction schemes of the shelling of unstained SiO_2 NPs using the L-arginine approach for the preparation of **Ruphenpy** complex-loaded SiO_2 shells **((US-ARGSiO$_2$)@Ruphenpy@ARGSiO$_2$))**. The synthesis of core/shell SiO_2 NPs comprises an unstained SiO_2 core (**US-ARGSiO$_2$**, the synthesis is shown in Figure S2) and a **Ruphenpy** complex-stained SiO_2 shell: TEOS (15.8 mmol), L-arginine (0.08 mmol), H_2O/cyclohexane (9.2:1).

Ruphenpy-stained SiO_2 core NPs shelled with an unstained SiO_2 shell (Ruphenpy@ARGSiO$_2$)@(US-ARGSiO$_2$)

After all parameters were set and the particle formation was induced as described for the **Ruphenpy**-stained SiO_2 NPs (**Ruphenpy@ARGSiO$_2$**), the growth of an unstained SiO_2 shell around the previously synthesised particles **Ruphenpy@ARGSiO$_2$** was performed following the procedure described above for 40 nm **US-ARGSiO$_2$** NPs. The reaction mixture was heated for an additional 20 h, before the particle dispersion was purified by dialysis as described before. A schematic presentation of the synthesis procedures is shown in Figure S5.

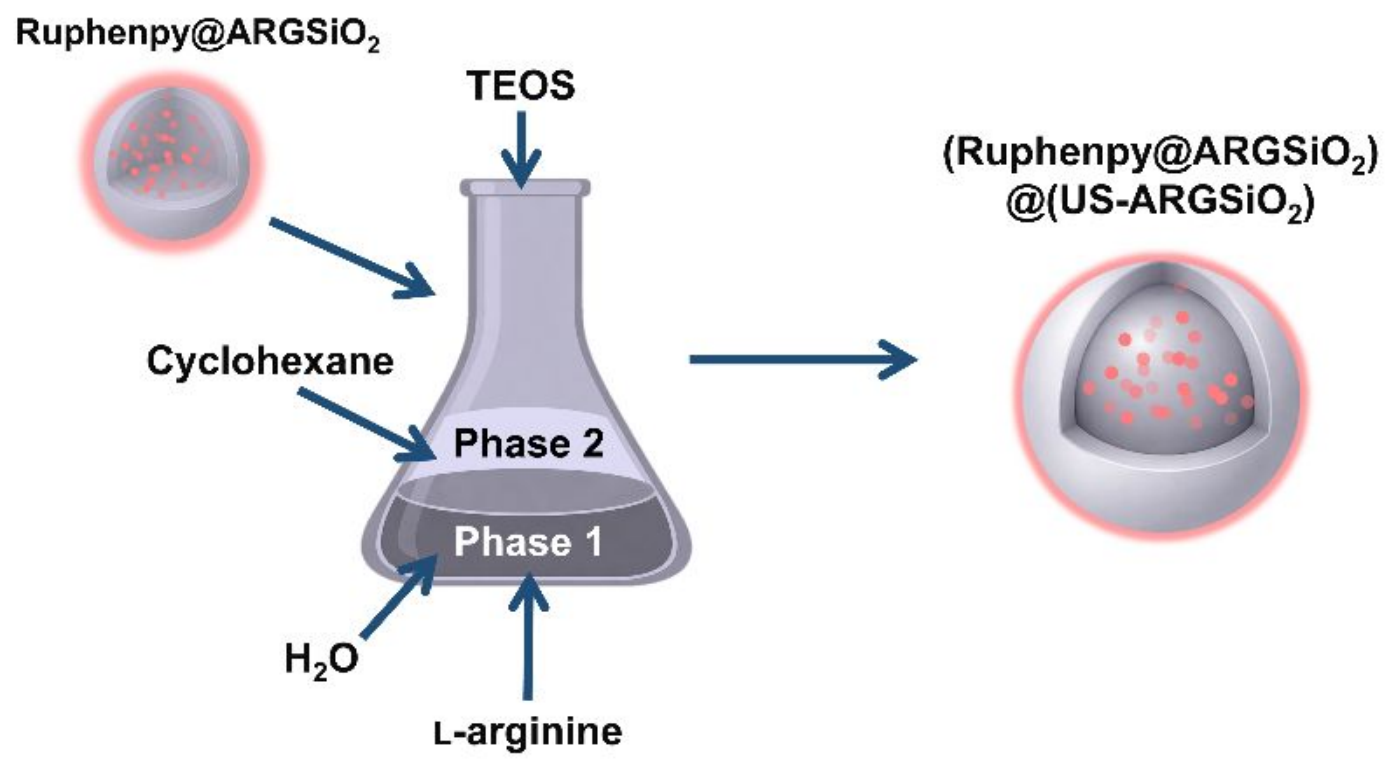

Figure S5. Reaction schemes of the shelling of stained with **Ruphenpy** SiO_2 NPs using the L-arginine approach for the preparation of unstained SiO_2 shells **((Ruphenpy@ARGSiO$_2$)@(US-ARGSiO$_2$))**. The synthesis of core/shell SiO_2 NPs comprises a stained with **Ruphenpy** SiO_2 core (**Ruphenpy@ARGSiO$_2$**, Synthesis is shown in Figure 1 (left bottom panel) and an unstained SiO_2 shell: TEOS (15.8 mmol), L-arginine (0.08 mmol), H_2O/cyclohexane (9.2:1).

S3.4. Increasing Ruphen and Ruphenpy loading concentration in ARGSiO_2 to enable the line-scan detection of Ru^{II}

Ruphen complex stained SiO_2 NPs (Ruphen@ARGSiO_2) made by the L-arginine approach [7]

For the preparation of **Ruphen**-loaded SiO_2 NPs, the above-described procedure was slightly modified by adding 500 µL (0.5 mmol) and 1000 µL (1 mmol) of **Ruphen** in DMF 20 min. after the addition of the corresponding amount of TEOS (0.55 mL, 2.5 mmol). A schematic representation of the synthesis procedure is shown in Figure 1 (bottom row, left part). These samples are described in Sections 3.5 and 3.7.

Ruphenpy-stained SiO_2 NPs (Ruphenpy@ARGSiO_2) made by the L-arginine approach [7]

For the preparation of **Ruphenpy**-loaded SiO_2 NPs, the above-described procedure was slightly modified by adding 200 µL (0.5 mmol), 500 µL (0.5 mmol), and 1000 µL (1 mmol) of **Ruphenpy** in DMF 20 min. after addition of the corresponding amount of TEOS (0.55 mL, 2.5 mmol). A schematic representation of the synthesis procedure is shown in Figure 1 (bottom row, left part). These samples will be characterized in Section 3.5 and Section 3.7.

S3.5. Dynamic light scattering (DLS)

Dynamic light scattering (DLS) was used to determine the number-weighted hydrodynamic diameter ($d_{h,0}$), the z-average (cumulant mean), the size distribution, and the degree of agglomeration of the prepared SiO_2 NPs. Measurements were carried out at 25 °C on a Malvern Panalytical Zetasizer Nano ZS (630 nm laser). For analysis, the particles were dispersed in 1 mL Milli-Q water and transferred to acrylic disposable cuvettes (Sarstedt, Germany). Data were collected in backscatter mode at 173° and evaluated using the cumulant approach. A refractive index of 1.4649 was used for silica and 1.3300 for water. The viscosity of water was set to 0.8872 cP. Each sample was measured three times, with 10 – 15 sub-runs per measurement and a 30-second pause between consecutive measurements. The z-average reported in Table S3 corresponds to the harmonic intensity-weighted mean diameter calculated using the cumulant mean analysis of the correlation function obtained by the measurements[8].

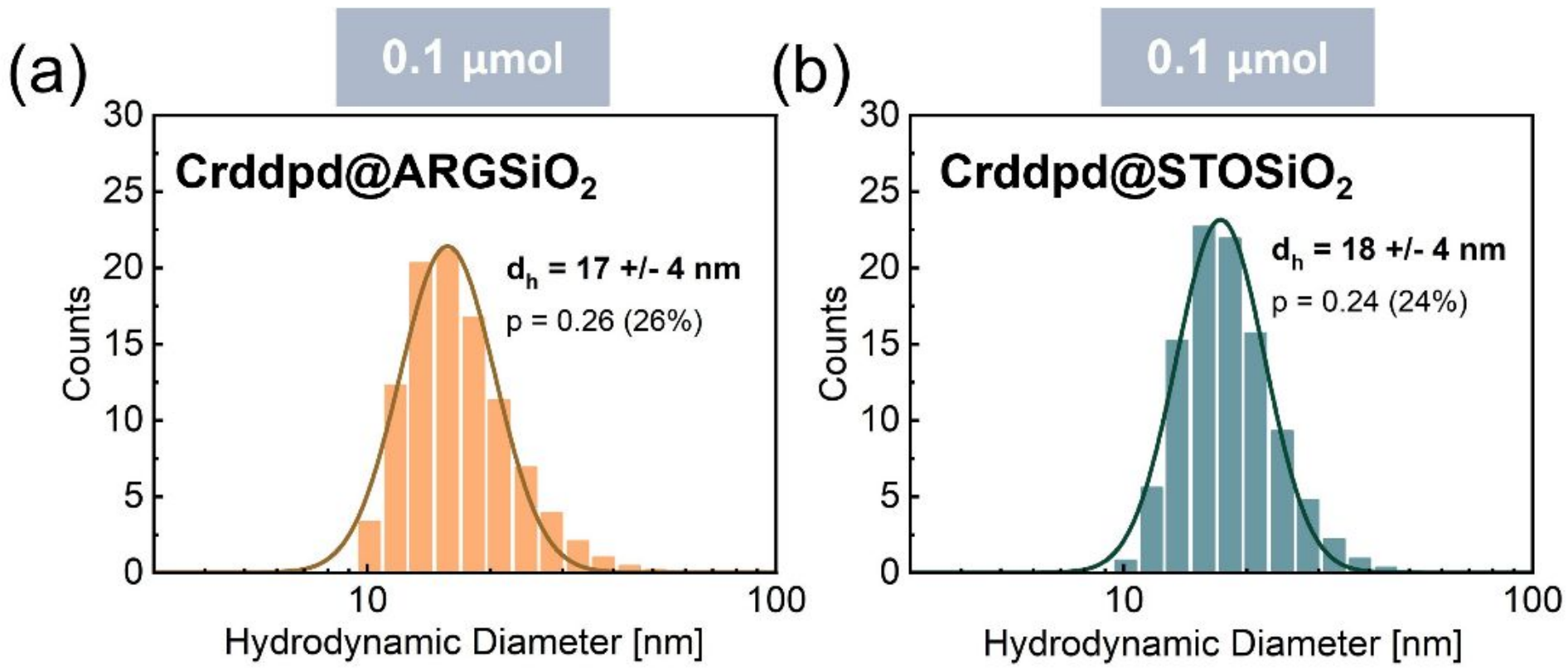

Figure S6. Histograms of the number-based hydrodynamic diameters ($d_{h,0}$) of **Crddpd**-stained SiO_2 NPs obtained by DLS measurements. **Crddpd**-stained SiO_2 NPs were prepared by the L-arginine approach (**Crddpd@ARGSiO₂**, (a)) and by the Stöber approach (**Crddpd@STOSiO₂**, (b)) using a concentration of 0.1 µmol **Crddpd** during synthesis. The particle sizes were obtained by using the lognormal distribution; p indicates the dispersity of the particle size.

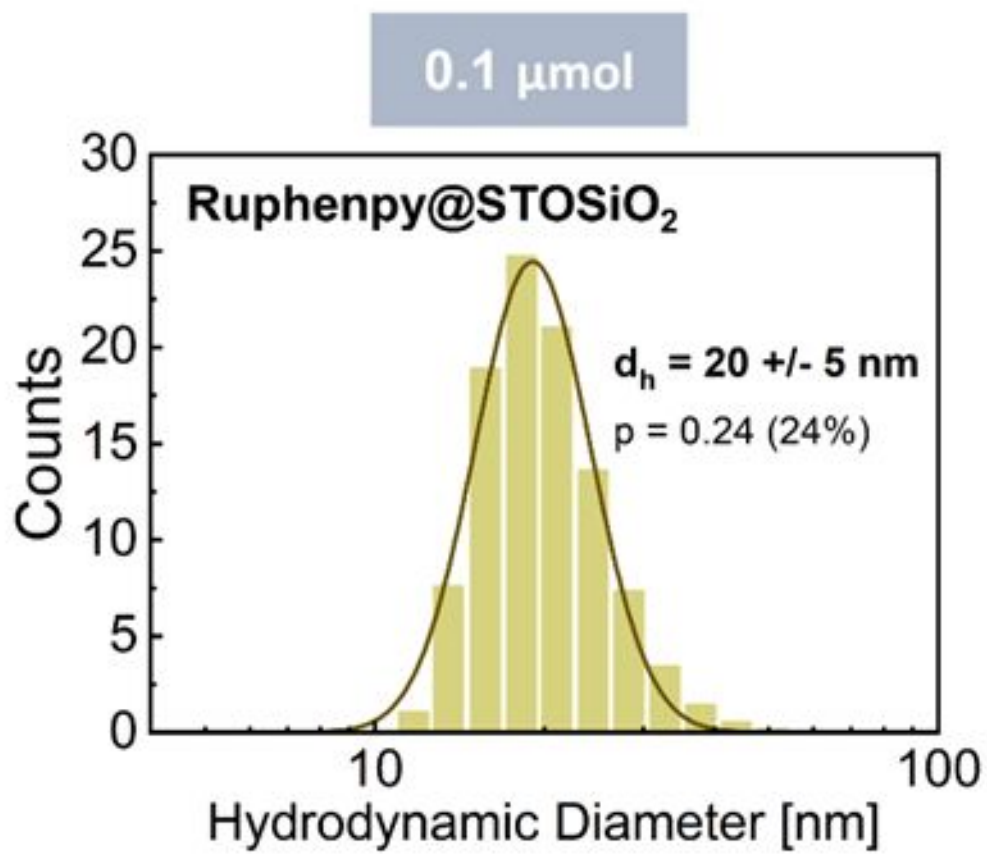

Figure S7. Histogram of $d_{h,0}$ of **Ruphenpy**-stained Stöber SiO_2 NPs (**Ruphenpy@STOSiO$_2$**) obtained by DLS measurements. The particle sizes were obtained by using the lognormal distribution; p indicates the dispersity of the particle size.

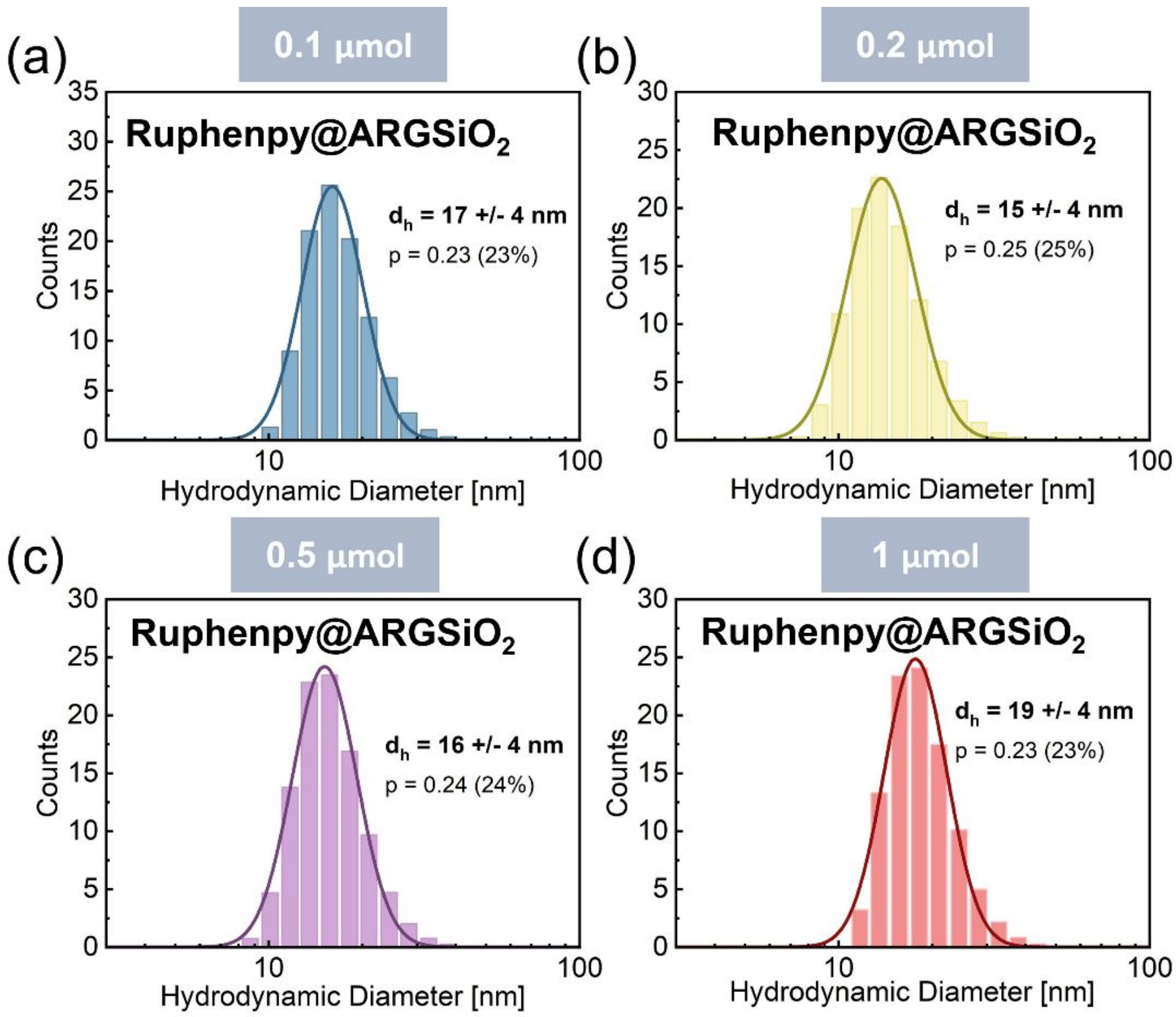

Figure S8. Histograms of $d_{h,0}$ of **Ruphenpy**-stained L-arginine silica nanoparticles (**Ruphenpy@ARGSiO$_2$**) measured by DLS. The particles were synthesised via the L-arginine route using different **Ruphenpy** loading concentrations: (a) 0.1 µmol, (b) 0.2 µmol, (c) 0.5 µmol, (d) 1.0 µmol. The particle sizes were obtained by using the lognormal distribution; p indicates the dispersity of the particle size. The 0.1 µmol loading concentration sample was used for the temperature-dependent and O_2-shielding efficiency studies, whereas the 0.2 µmol, 0.5 µmol, and 1.0 µmol samples were only prepared for the line-scanning experiments.

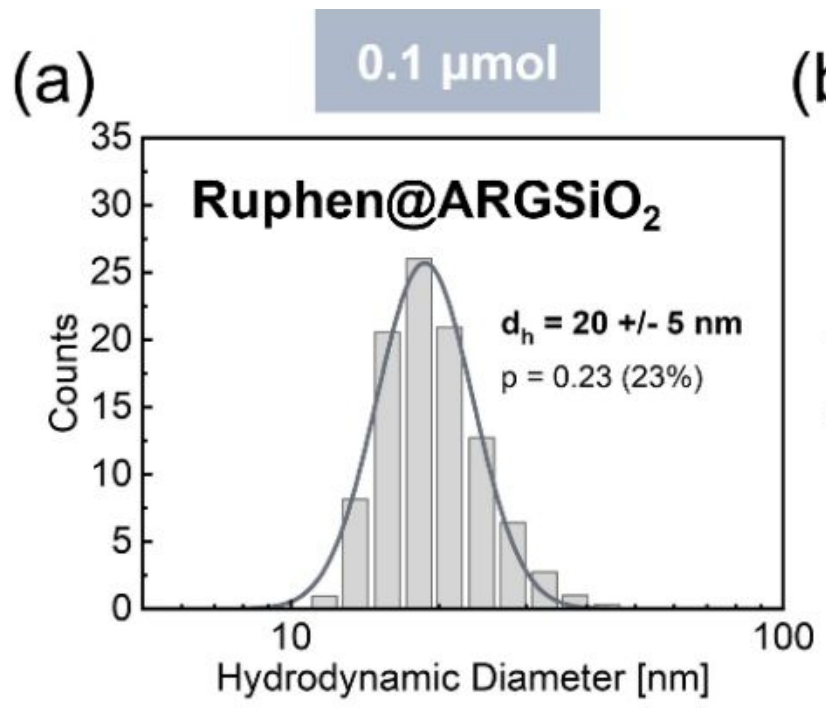

Figure S9. Histograms of $d_{h,0}$ of **Ruphen**-stained L-arginine silica nanoparticles (**Ruphen@ARGSiO$_2$**) measured by DLS. The particles were synthesised via the L-arginine route using different **Ruphen** loading concentrations: (a) 0.1 µmol, (b) 0.5 µmol, (c) 1.0 µmol. The particle sizes were obtained by using the lognormal distribution; p indicates the dispersity of the particle size. The 0.1 µmol loading-concentration sample was used for the temperature-dependent and oxygen-shielding efficiency studies, whereas the 0.5 µmol and 1.0 µmol samples were solely prepared for the line-scanning experiments.

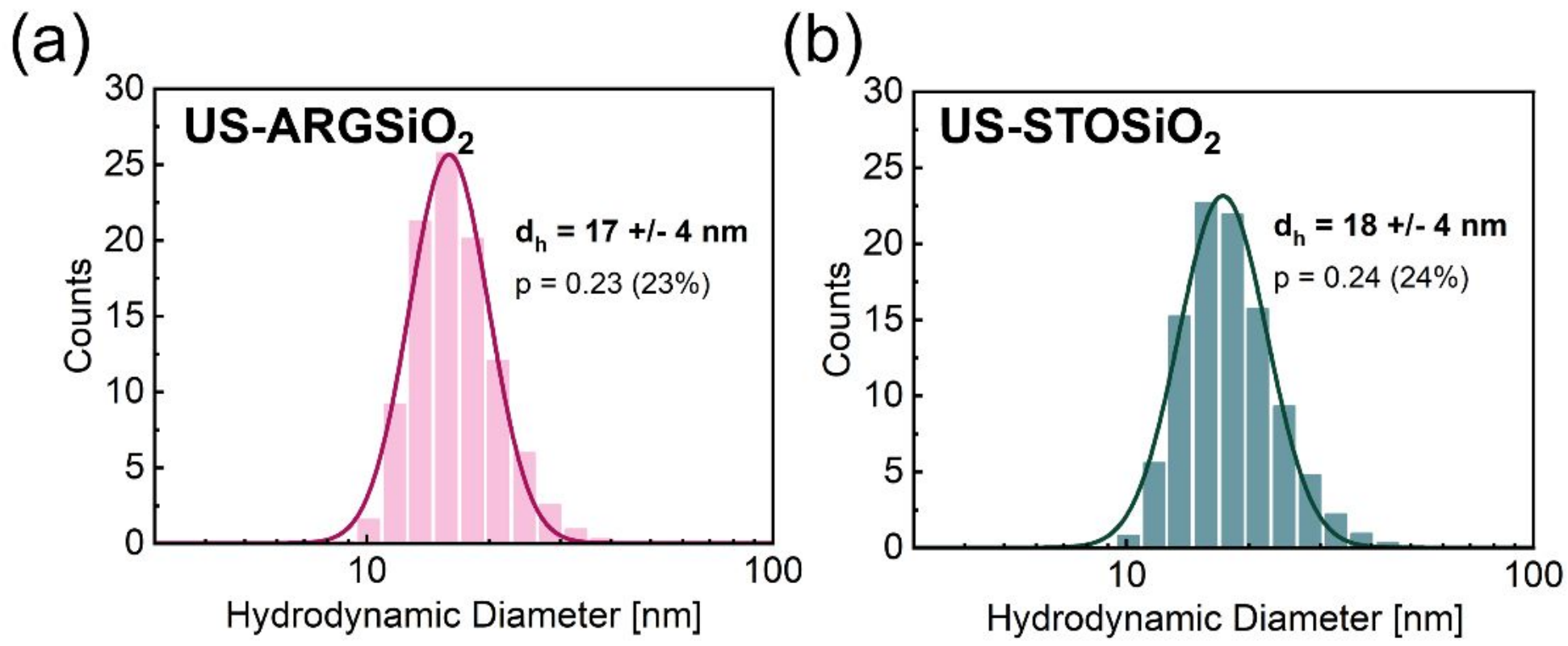

Figure S10. Histograms of $d_{h,0}$ of unstained SiO_2 NPs obtained by DLS measurements. a) Unstained SiO_2 NPs were prepared by the L-arginine approach (**US-ARGSiO$_2$**). b) Unstained SiO_2 NPs were synthesized by the Stöber approach (**US-STOSiO$_2$**). The particle sizes were obtained by using the lognormal distribution; p indicates the dispersity of the particle size.

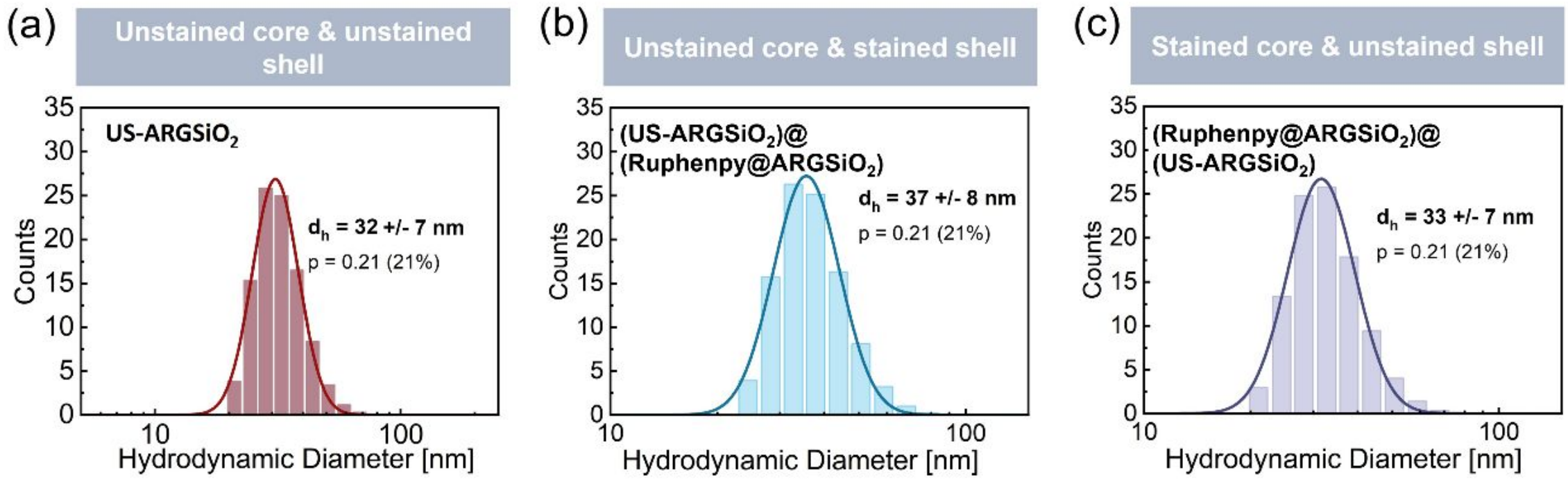

Figure S11. Histograms of $d_{h,0}$ of unstained and **Ruphenpy**-stained SiO_2 NPs obtained by DLS measurements. a) Unstained SiO_2 NPs were prepared by the L-arginine approach with an additional re-growth step (40 nm **US-ARGSiO$_2$**) around previously synthesised particle (25 nm **US-ARGSiO$_2$**). b) **Ruphenpy**-stained SiO_2 NPs shell synthesized on top of an unstained core (25 nm **US-ARGSiO$_2$**) by the L-arginine approach **((US-ARGSiO$_2$)@(Ruphen@ARGSiO$_2$))**. (c) **Ruphen**-stained SiO_2 NPs core **((Ruphenpy@ARGSIO$_2$))** with an unstained shell by the L-arginine approach **((Ruphen@ARGSiO$_2$)@(US-ARGSiO$_2$))**. The particle sizes were obtained by using the lognormal distribution; p indicates the dispersity of the particle size.

As evidenced by the number-weighted DLS size distributions in Figures S6-S9, S11, the presence of the Cr^{III} or Ru^{II} complexes in the reaction mixture during particle formation has almost no effect on particle size with the resulting changes being in the same or as the standard deviations of these measurements. Please note that$d_{h,0}$ is a calculated value with the assumption of perfectly spherical particles, leading to smaller sizes than the diameters derived from TEM. Overall, incorporation of the Cr^{III} or Ru^{II} complexes did not significantly affect the particle size relative to the corresponding unstained model SiO_2 nanoparticles prepared using either the Stöber process or the L-arginine approach.

S3.6. Zeta potential (ζ-potential) measurements

ζ-potential measurements of the SiO_2 NPs were carried out in Milli-Q water using a Zetasizer Nano ZS (Malvern Panalytical, Germany) at 25 °C.. Each sample was measured three times (10-100 sub-runs per measurement) with 30 s breaks in between. Measurements were conducted in disposable folded capillary cells (DTS 1070, Malvern Panalytical, UK) and values were calculated from the particle electrophoretic mobility using the Einstein-Smoluchowski theory.

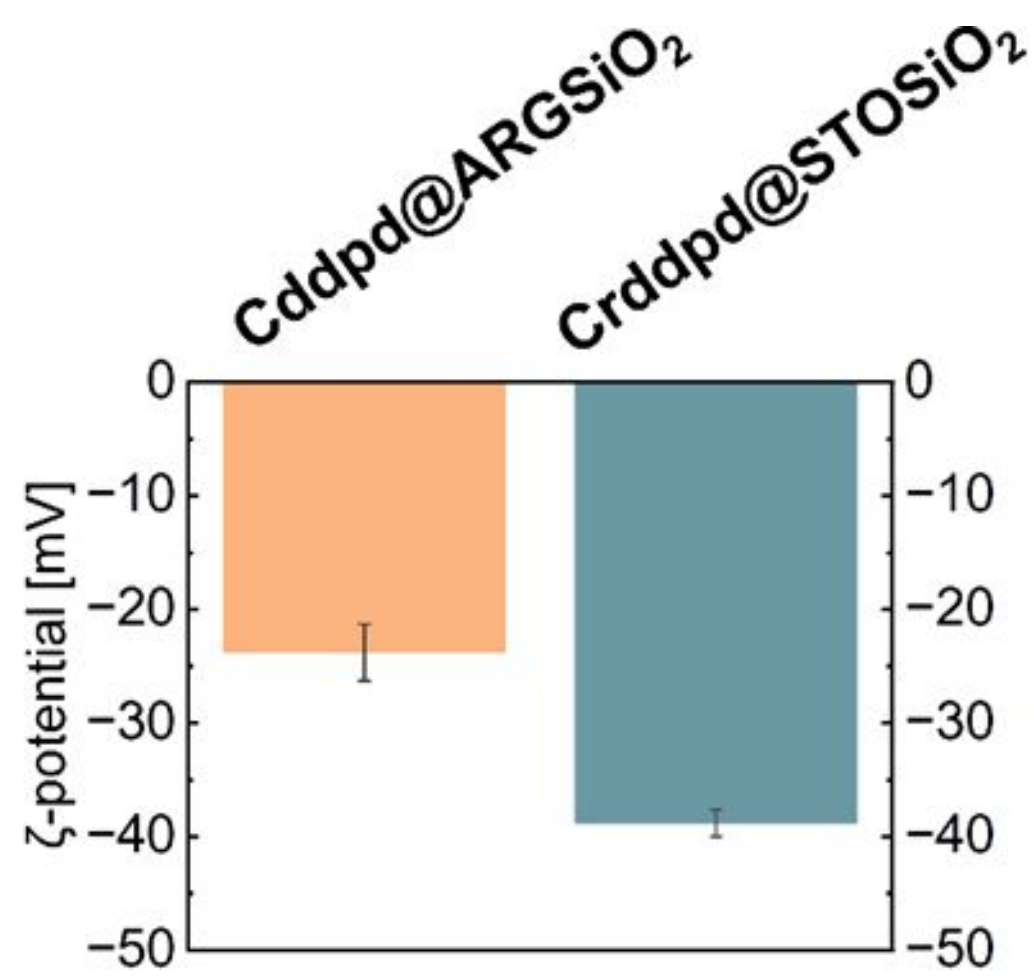

Figure S12. ζ-potential measurements of **Crddpd**-stained SiO_2 NPs prepared by the L-arginine approach (**Crddpd@ARGSiO₂**) and by the Stöber approach (**Crddpd@STOSiO₂**).

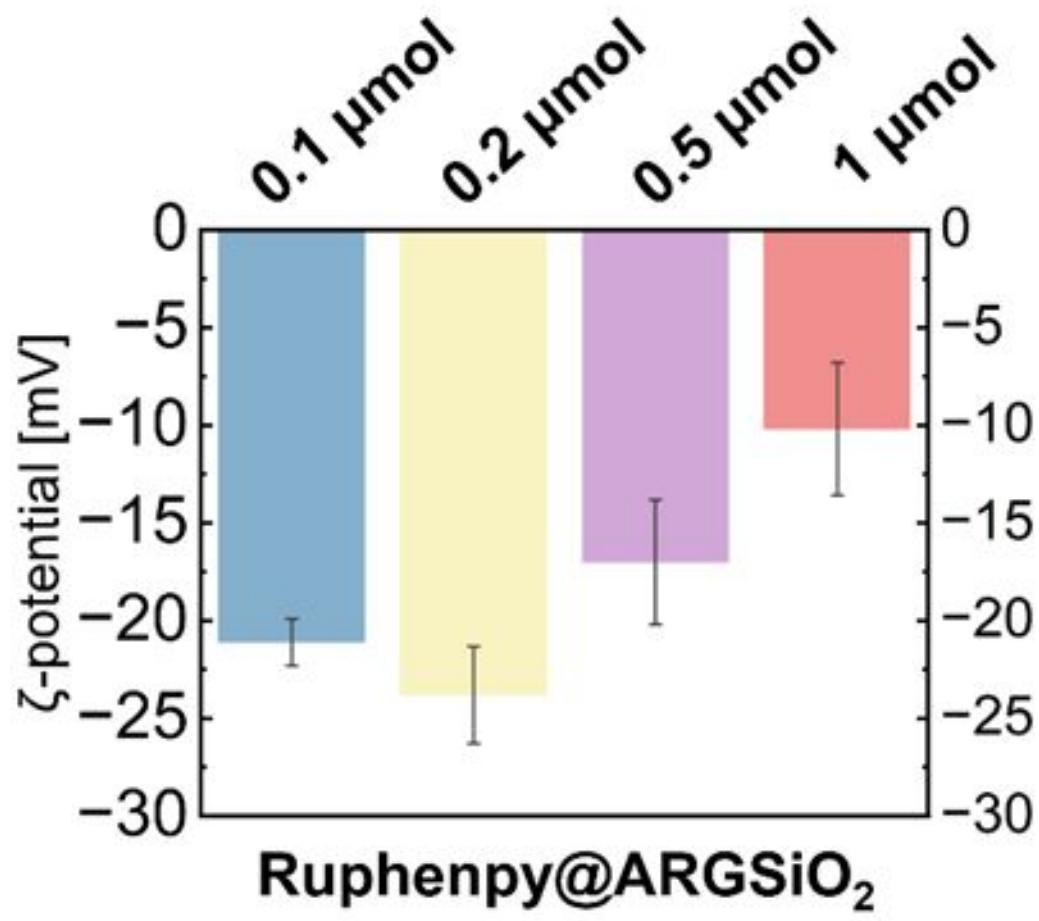

Figure S13. ζ-potential measurements of **Ruphenpy**-stained SiO_2 NPs prepared by the L-arginine approach (**Ruphenpy@ARGSiO₂**). The particles were synthesized via the L-arginine route using different **Ruphenpy** loading concentrations: 0.1 µmol, 0.2 µmol, 0.5 µmol and 1.0 µmol.

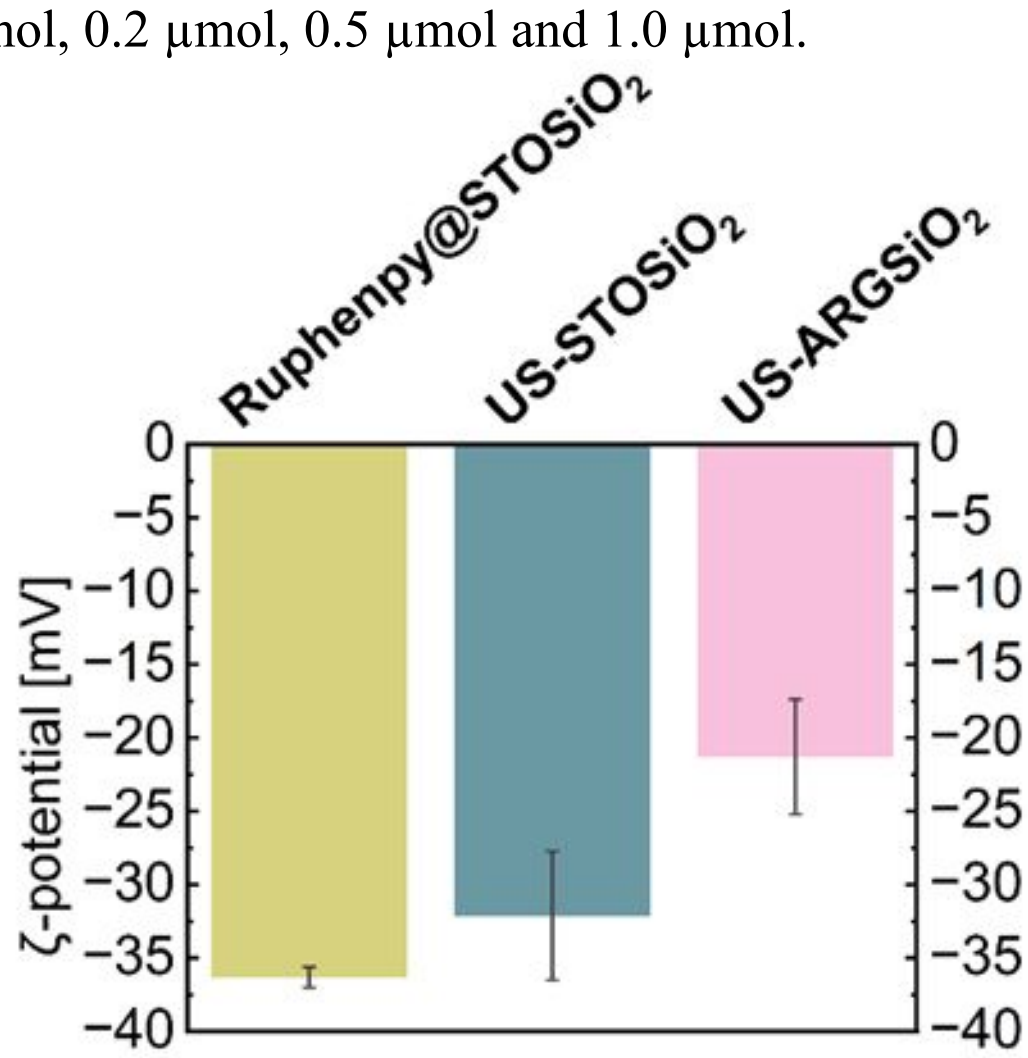

Figure S14. ζ-potential measurements of **Ruphenpy**-stained Stöber SiO_2 NPs (**Ruphenpy@STOSiO₂**), unstained SiO_2 NPs prepared via Stöber approach (**US-STOSiO₂**) and unstained SiO_2 NPs prepared via L-arginine approach (**US-ARGSiO₂).**

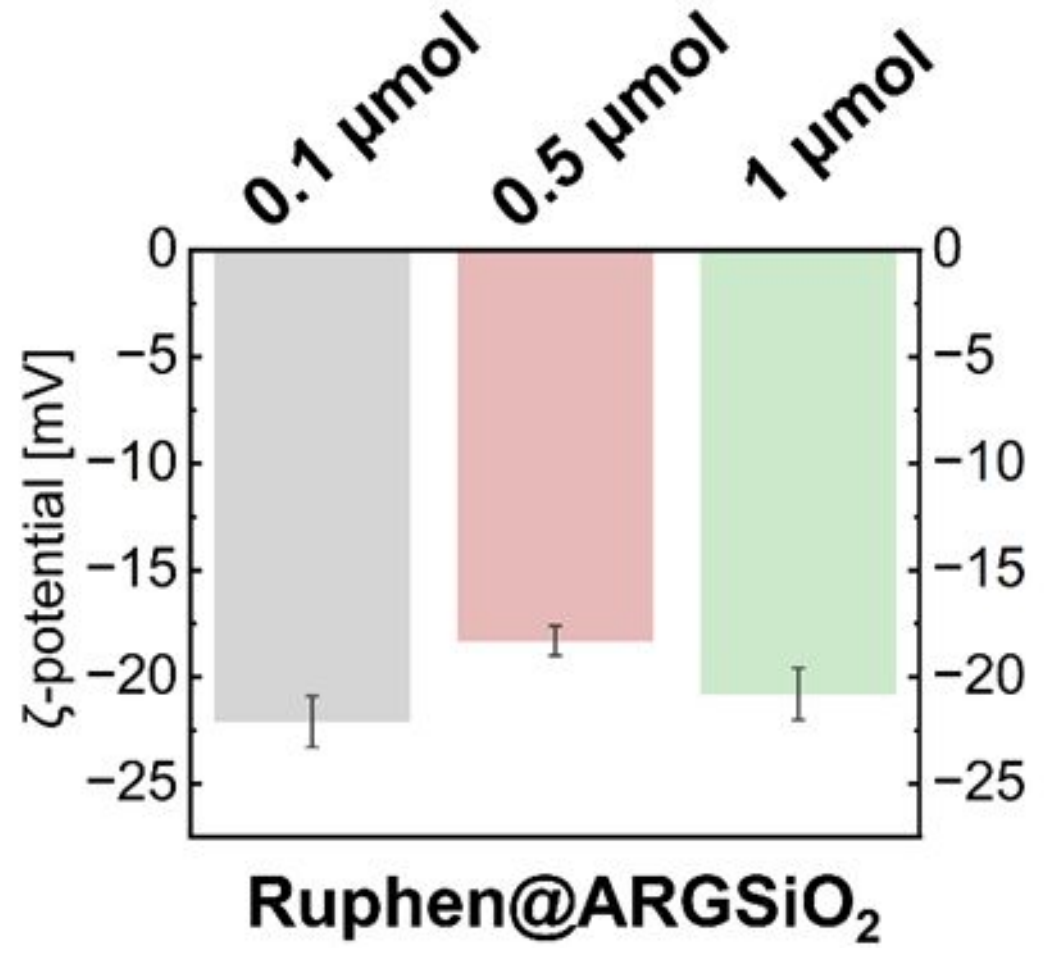

Figure S15. ζ-potential measurements of **Ruphen**-stained SiO_2 NPs prepared by the L-arginine approach (**Ruphen@ARGSiO$_2$**). The particles were synthesized via the L-arginine route using different **Ruphen** loading concentrations: 0.1 µmol, 0.5 µmol and 1.0 µmol

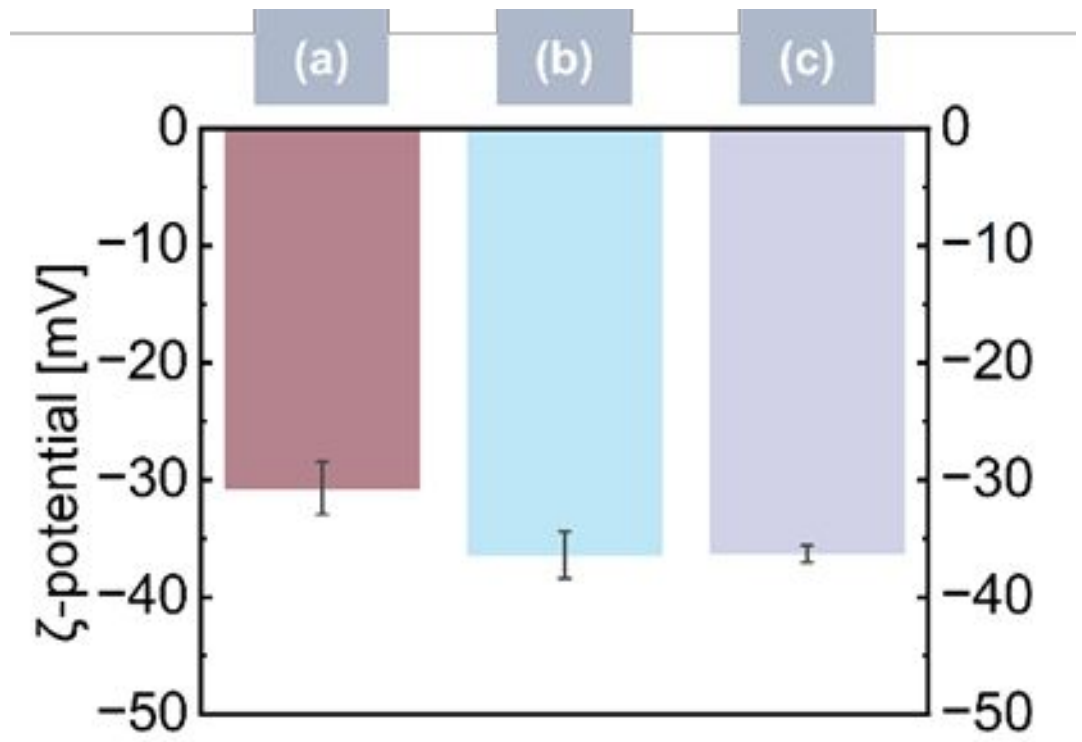

Figure S16. ζ-potential measurements of (a) Unstained SiO_2 NPs were prepared by the L-arginine approach with an additional re-growth step (40 nm **US-ARGSiO$_2$**) around previously synthesized particle (25 nm **US-ARGSiO$_2$**). (b) **Ruphenpy**-stained SiO_2 shell synthesized on top of an unstained SiO_2 core (25 nm **US-ARGSiO$_2$**) by the L-arginine approach (**(US-ARGSiO$_2$@Ruphen-ARGSiO$_2$)**). (c) **Ruphen**-stained SiO_2 NPs core **(Ruphenpy@ARGSIO$_2$)** with an unstained shell made by the L-arginine approach **(Ruphen@ARGSiO$_2$@US-ARGSiO$_2$)**.

The ζ-potential measurements provide additional information on particle surface charge, colloidal stability, and purification efficiency. The comparable ζ-potential values obtained for dialyzed **Crddpd**-, **Ruphen**-, and **Ruphenpy**-loaded SiO_2 NPs relative to the corresponding unstained reference particles indicate that the overall surface charge is largely preserved after luminophore incorporation and purification. This suggests that the complexes are not immobilized on the external particle surface to a significant extent, but are predominantly incorporated within the silica matrix. In contrast, for the series synthesized with increasing **Ruphenpy** concentration, a progressive decrease in the magnitude of the negative ζ-potential was observed, suggesting that at higher **Ruphenpy** concentrations the Ru^{II} dyad may partly associate with the particle surface and/or form Ru^{II}-enriched domains, such as aggregate- or

crystallite-like species. This behavior was not observed for **Ruphen**, that also show a positive charge, at higher loading concentrations.

Overall, Stöber-type SiO_2 particles exhibit more negative ζ-potentials than the corresponding L-arginine-derived SiO_2 particles. This difference is attributed to the different surface chemistry produced by the two sol–gel synthesis routes. Stöber SiO_2 particles are formed by ammonia-catalyzed hydrolysis and condensation of TEOS in EtOH/H_2O, whereas L-arginine-derived particles are formed in a biphasic water/cyclohexane system using L-arginine as catalyst. These different reaction conditions can affect the number and typeof negatively charged silanol groups at the particle surface. In addition, residual catalyst or surface-associated species, , and the incorporated luminophore can influence the measured ζ-potential, as previously reported for L-arginine-derived dye-doped SiO_2 nanoparticles [6]**.** This interpretation is also consistent with our previous study on Cr^{III}-complex-stained SiO_2 nanoparticles, where differences between Stöber- and L-arginine-derived silica networks were attributed to differences in silica-network and surface chemistry [9].

Importantly, both $ARGSiO_2$ and $STOSiO_2$ particles are sufficiently negatively charged and colloidally stable under the applied conditions, as supported by DLS and TEM. Therefore, ζ-potential is used here mainly as an indicator of colloidal stability and successful purification, not as a direct descriptor of O_2 permeability.

S3.7. Electron microscopy (EM) measurements

Electron microscopy (TEM) measurements were performed at the Electron Microscopy Facility at IST Austria. High-resolution Transmission Electron Microscopy (HR-TEM), High-Angle Annular Dark-Field Scanning Transmission Electron Microscopy (HAADF-STEM), and energy-dispersive X-ray (EDX) spectroscopy measurements were carried out using a S/TEM Jeol JEM2800 with an accelerating voltage of 200 kV to characterize the size, shape, and particle architecture of the SiO_2 NPs as well as the distribution of the Cr^{III} complex, Ru^{II} complex and Ru^{II}-dyad molecule within the SiO_2 NPs. The EM micrographs were collected with a CMOS TEM camera TemCam-XF416, and the EDX data by an EDX detector Jeol Centurio. This detector is a large solid angle silicon drift detector with 100 mm^2 active area for ultrafast elemental mapping of S/TEM samples. All samples were prepared by drop-casting onto 200-mesh copper grids coated with a carbon film. Subsequently, the samples were dried in an oven at 70 °C before usage. The particle size distribution was representatively determined for a randomly chosen sample of over 100 particles. All images were adjusted for brightness, contrast, and picture size using ImageJ (Version 1.52e). The reported size distribution was

measured using the respective EM software package. Parts of the transmission electron microscopy (TEM) measurements were performed at the electron microscopy center at BAM (Figures 2(g) and Figure S18) on a F200S Microscope (ThermoFisher Scientific, USA) by adding 10 µL of diluted particle suspensions onto a TEM grid (Formvar/Carbon coated, 400 Mesh, Plano GmbH, Germany) and dried overnight.

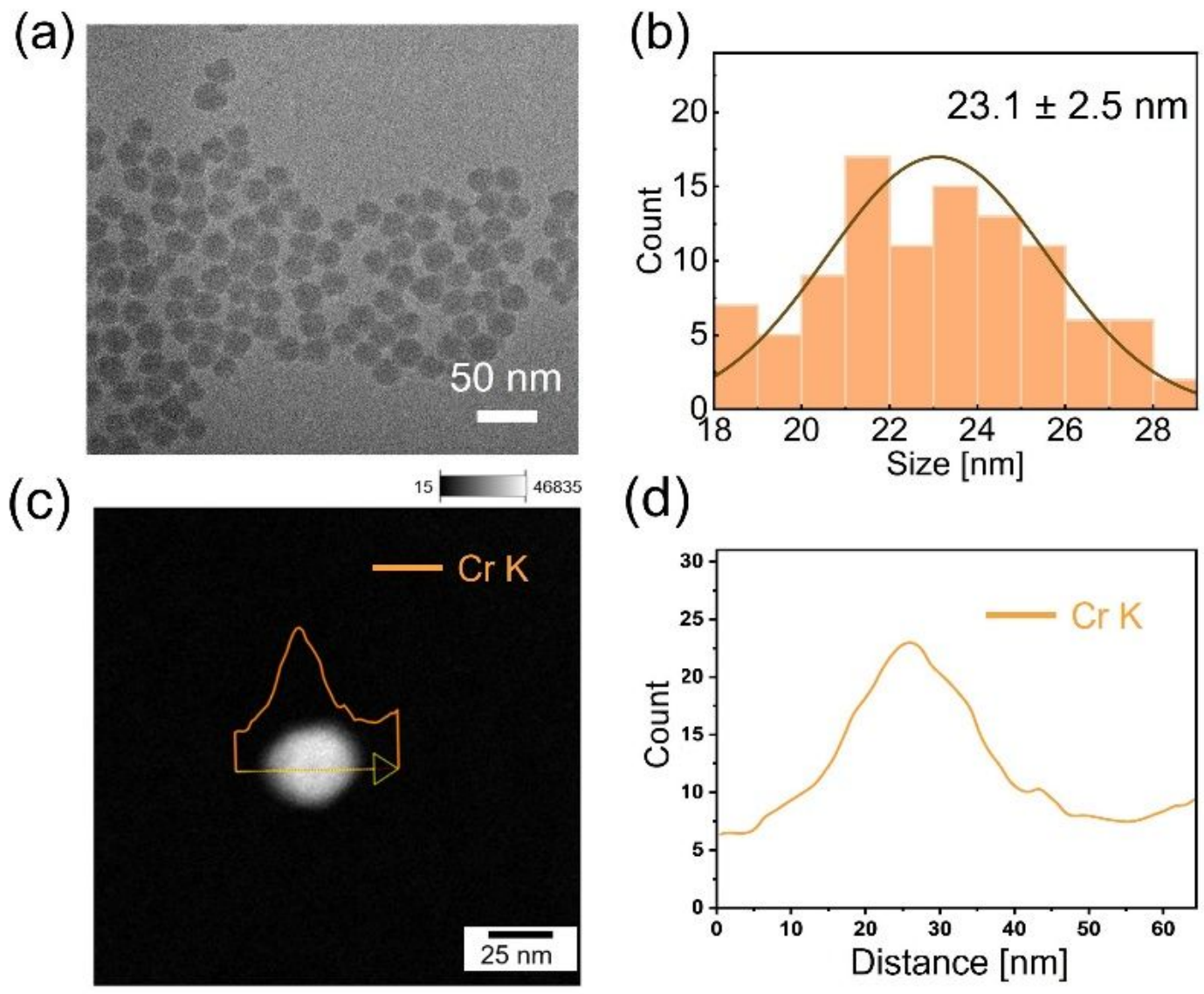

Figure S17. Morphology and elemental distribution of **Crddpd@ARGSiO$_2$** nanoparticles. (a) Representative TEM micrograph of **Crddpd@ARGSiO$_2$**. (b) Particle size distribution histogram obtained from analysis of TEM images. (c) Image of a randomly selected **Crddpd@ARGSiO$_2$** particle with the EDX line-scan path indicated. (d) Corresponding Cr–EDX line-scan profile, confirming the presence of Cr across the particle.

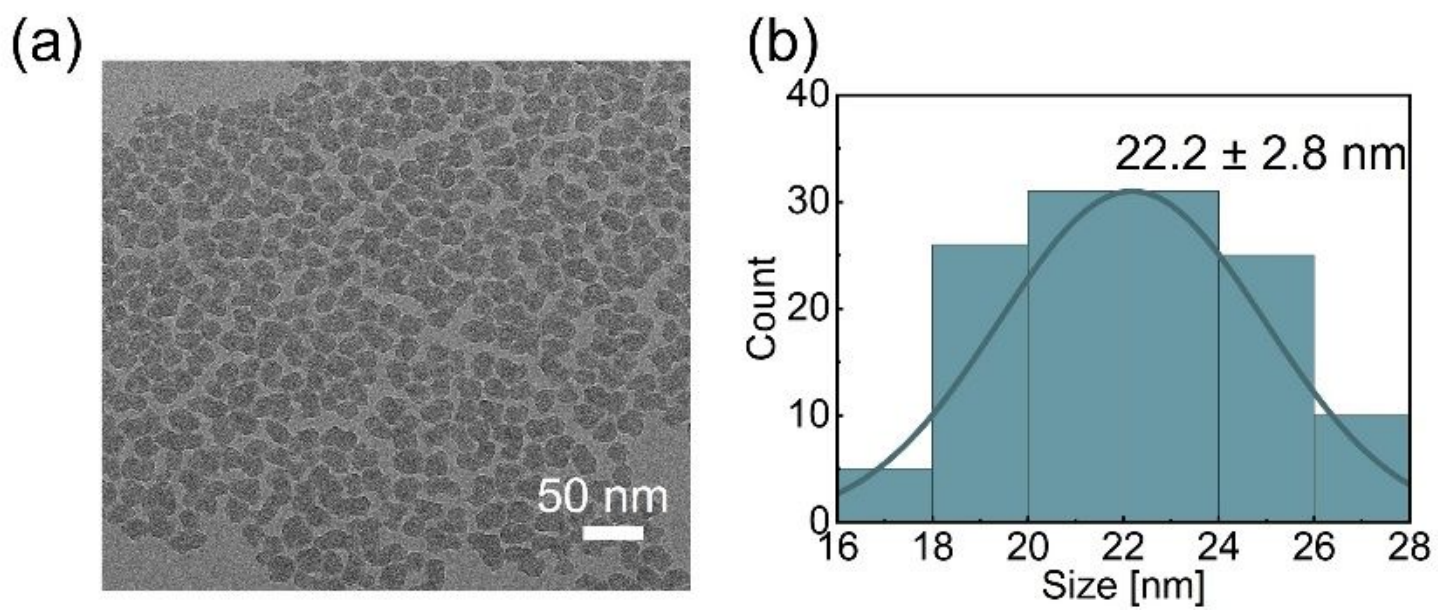

Figure S18. Morphology of **Crddpd@STOSiO$_2$** nanoparticles. (a) Representative TEM micrograph of **Crddpd@STOSiO$_2$**. (b) Particle size distribution histogram obtained from analysis of TEM images.

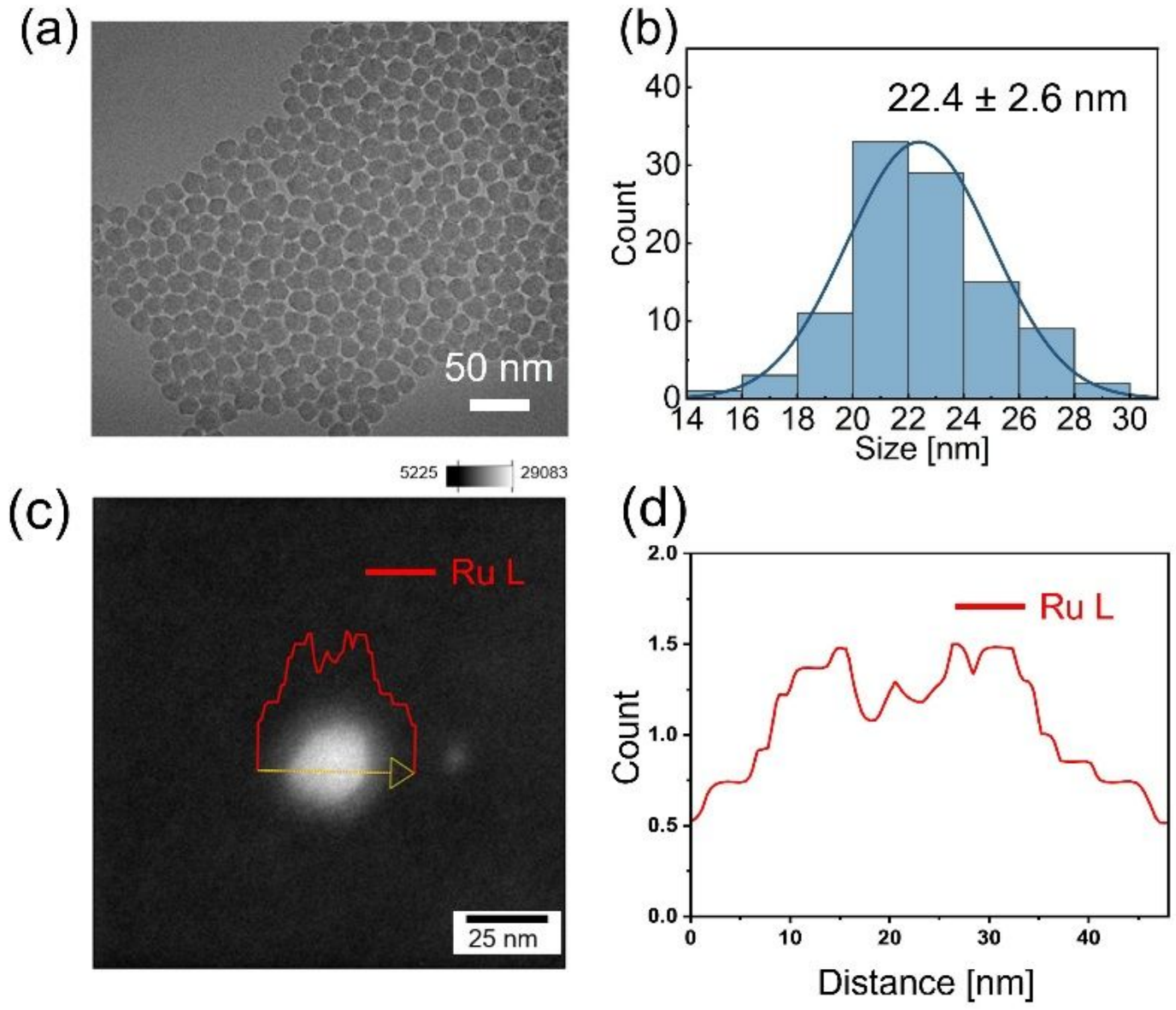

Figure S19. Morphology and elemental distribution of **Ruphenpy@ARGSiO$_2$** nanoparticles (**Ruphenpy** loading concentration: 0.1 µmol). (a) Representative TEM micrograph of **Ruphenpy@ARGSiO$_2$**. (b) Particle size distribution histogram obtained from TEM image analysis. (c) Image of a randomly selected **Ruphenpy@ARGSiO$_2$** particle with the EDX line-scan path indicated. (d) Corresponding Ru–EDX line-scan profile, made to confirm the presence of Ru across the particle.

Please note that for the measurements shown in Figures S19 and S20, EDX is on the limit of significance as the Ru Lα line is challenging to detect due to its low energy and its close neighborhood to the Cl Kα line at ~2.62 keV. Hence, as a reliable proof for the presence of ruthenium in the silica particles, we utilized ICP-MS measurements of dissolved luminophore-stained SiO_2 NPs which provide an improved sensitivity for ruthenium.

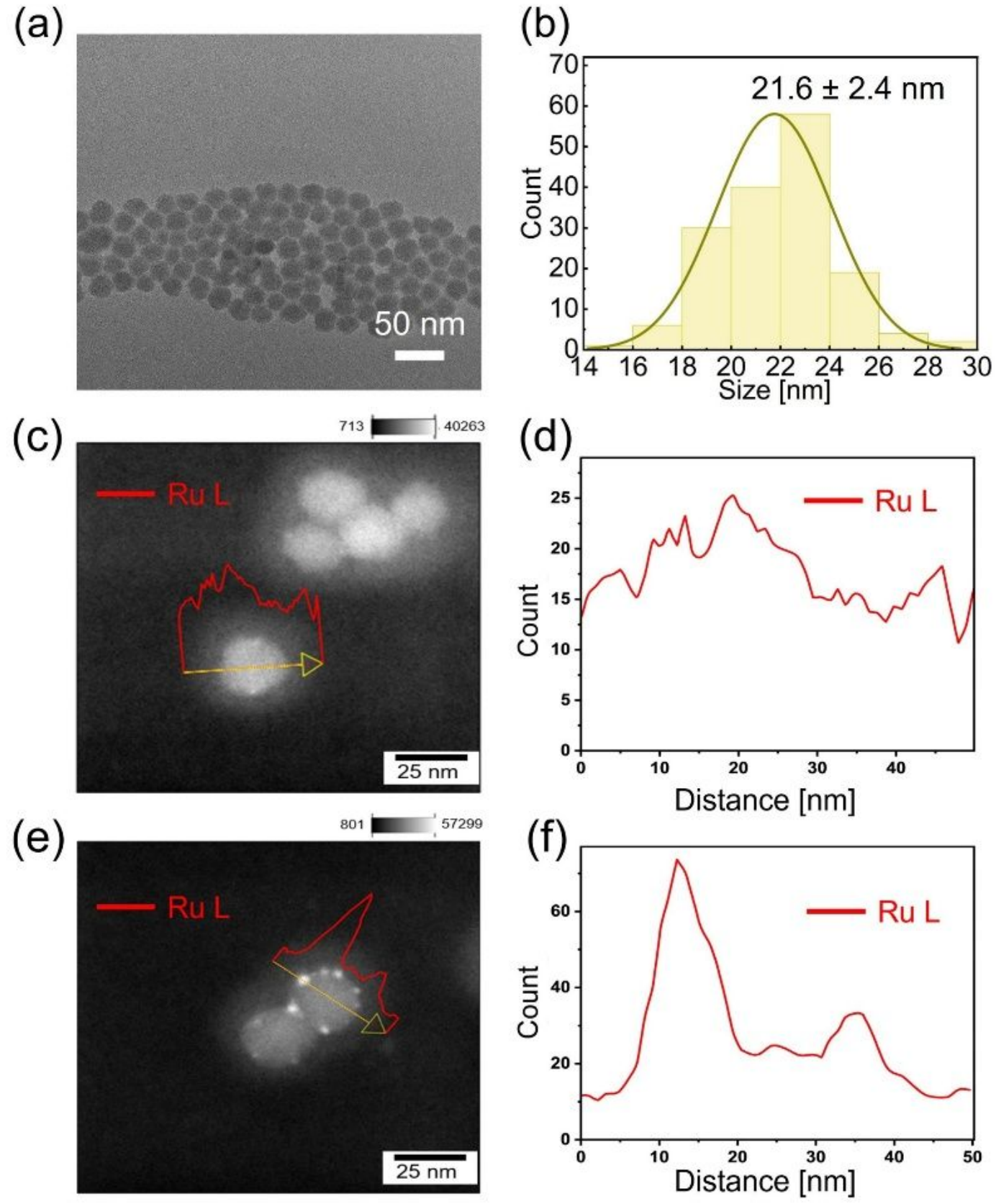

Figure S20. Morphology and elemental distribution of **Ruphenpy@ARGSiO$_2$** nanoparticles (**Ruphenpy** loading concentration: 0.2 µmol). (a) Representative TEM micrograph of **Ruphenpy@ARGSiO$_2$**. (b) Particle size distribution histogram obtained from TEM image analysis. (c) Image of a randomly selected **Ruphenpy@ARGSiO$_2$** particle with the EDX line-scan path indicated. (d) Corresponding Ru–EDX line-scan profile, confirming the presence of Ru across the particle. (e) Image of a second randomly selected **Ruphenpy@ARGSiO$_2$** particle with the EDX line-scan path indicated. (f) Corresponding Ru–EDX line-scan profile, confirming the presence of Ru across the particle.

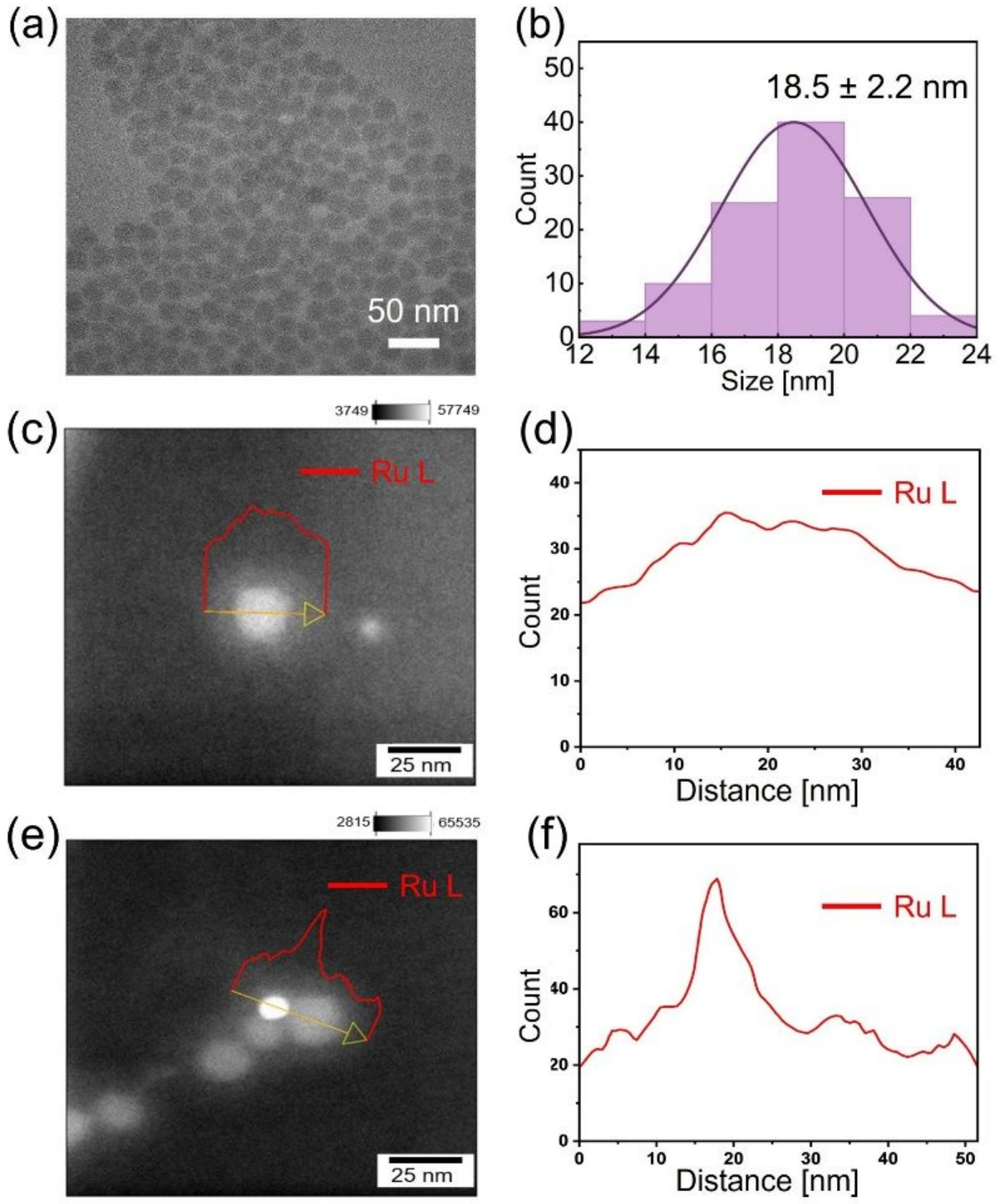

Figure S21. Morphology and elemental distribution of **Ruphenpy@ARGSiO$_2$** nanoparticles (**Ruphenpy** loading concentration: 0.5 μmol). (a) Representative TEM micrograph of **Ruphenpy@ARGSiO$_2$**. (b) Particle size distribution histogram obtained from TEM image analysis. (c) Image of a randomly selected **Ruphenpy@ARGSiO$_2$** particle with the EDX line-scan path indicated. (d) Corresponding Ru–EDX line-scan profile, confirming the presence of Ru across the particle. (e) Image of a second randomly selected **Ruphenpy@ARGSiO$_2$** particle with the EDX line-scan path indicated. (f) Corresponding Ru–EDX line-scan profile, confirming the presence of Ru across the particle.

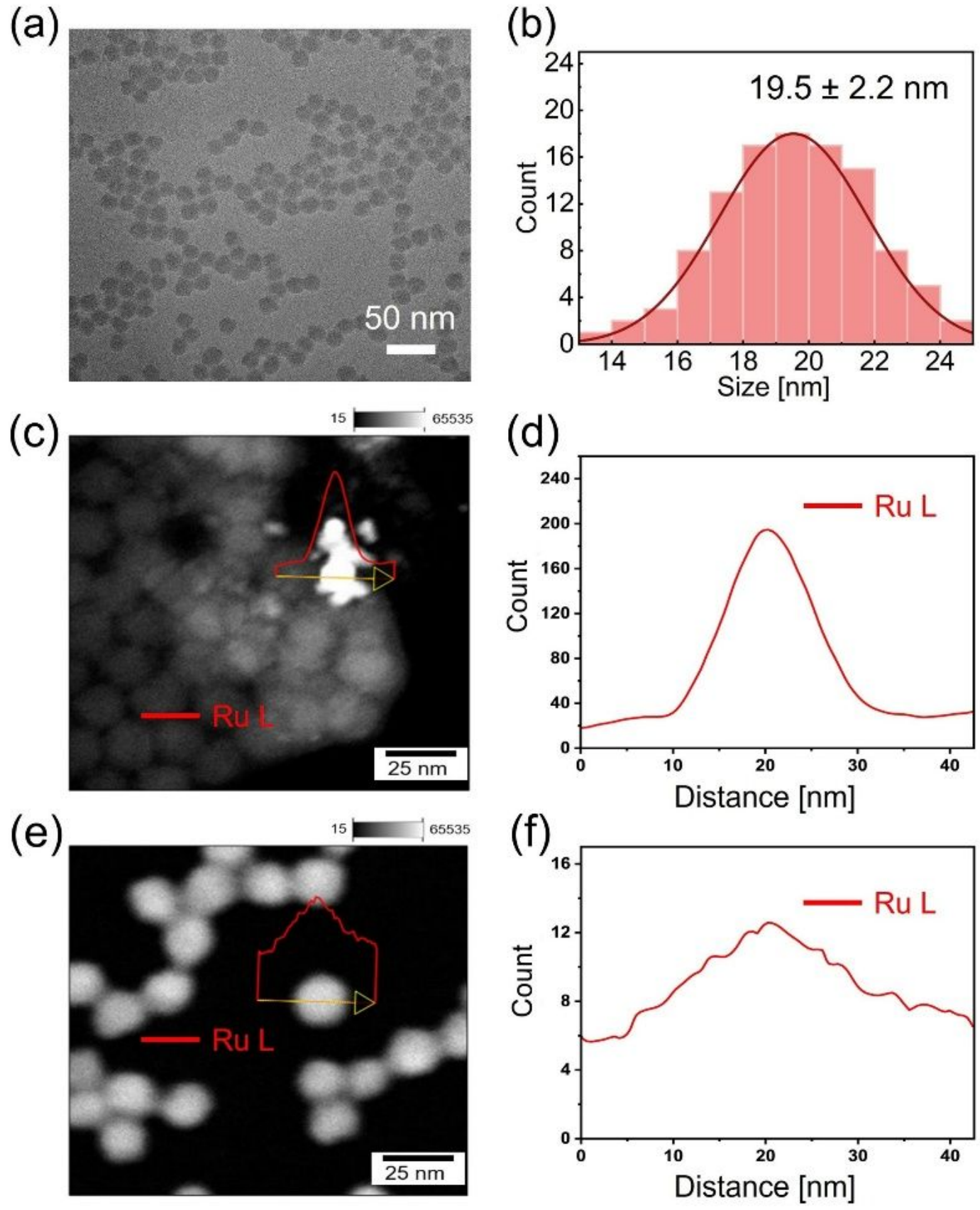

Figure S22. Morphology and elemental distribution of **Ruphenpy@ARGSiO$_2$** nanoparticles (**Ruphenpy** loading concentration: 1 µmol). (a) Representative TEM micrograph of **Ruphenpy@ARGSiO$_2$**. (b) Particle size distribution histogram obtained from TEM image analysis. (c) Image of a randomly selected **Ruphenpy@ARGSiO$_2$** particle with the EDX line-scan path indicated. (d) Corresponding Ru–EDX line-scan profile, confirming the presence of Ru across the particle. (e) Image of a second randomly selected **Ruphenpy@ARGSiO$_2$** particle with the EDX line-scan path indicated. (f) Corresponding Ru–EDX line-scan profile, confirming the presence of Ru across the particle.

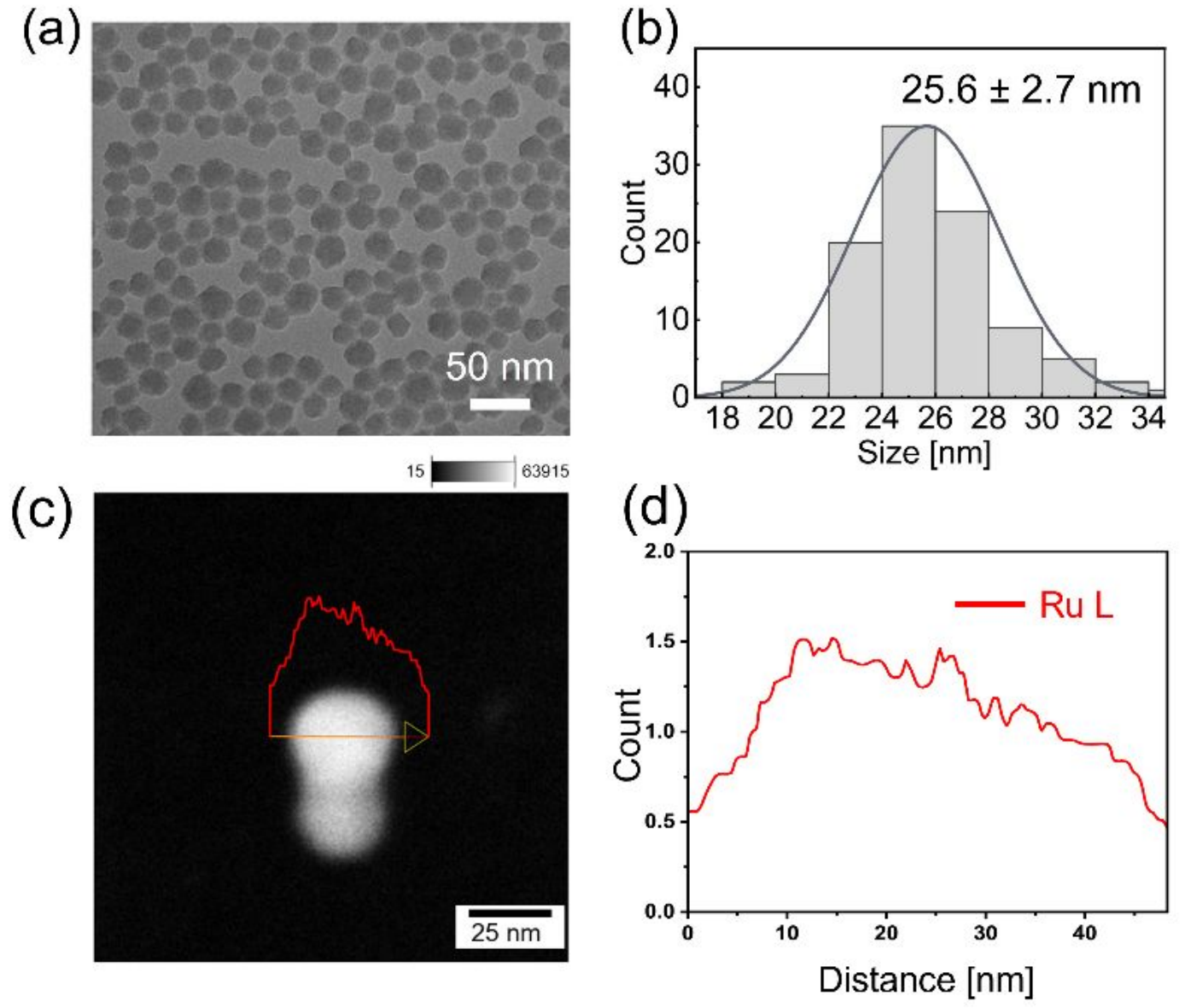

Figure S23. Morphology and elemental distribution of **Ruphen@ARGSiO$_2$** nanoparticles (**Ruphen** loading concentration: 0.1 µmol). (a) Representative TEM micrograph of **Ruphen@ARGSiO$_2$**. (b) Particle size distribution histogram obtained from TEM image analysis. (c) Image of a randomly selected **Ruphen@ARGSiO$_2$** particle with the EDX line-scan path indicated. (d) Corresponding Ru–EDX line-scan profile, made to confirm the presence of Ru across the particle.

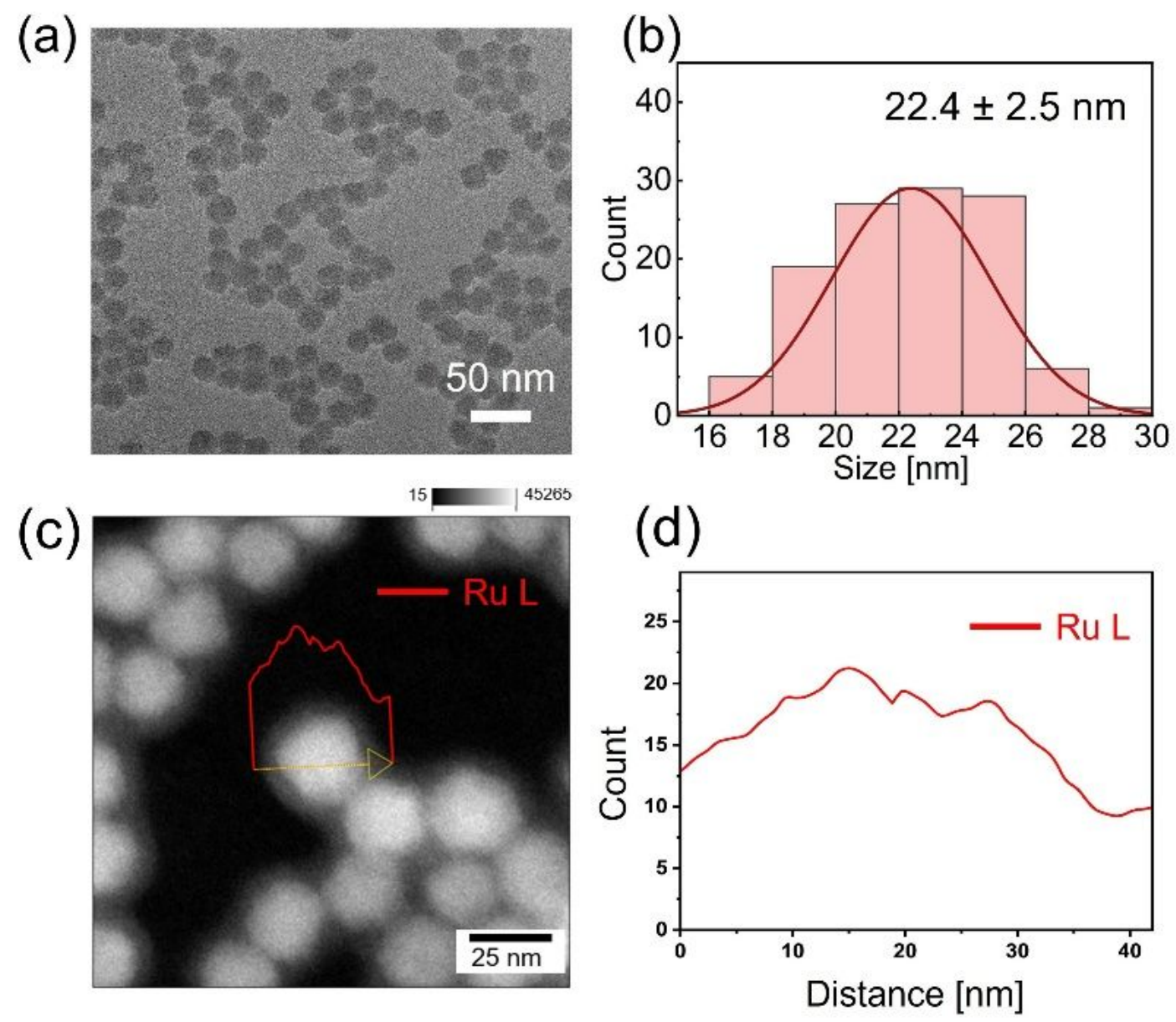

Figure S24. Morphology and elemental distribution of **Ruphen@ARGSiO$_2$** nanoparticles (**Ruphen** loading concentration: 0.5 µmol). (a) Representative TEM micrograph of **Ruphen@ARGSiO$_2$**. (b) Particle size distribution histogram obtained from TEM image analysis. (c) Image of a randomly selected **Ruphen@ARGSiO$_2$** particle with the EDX line-scan path indicated. (d) Corresponding Ru–EDX line-scan profile, made to confirm the presence of Ru across the particle.

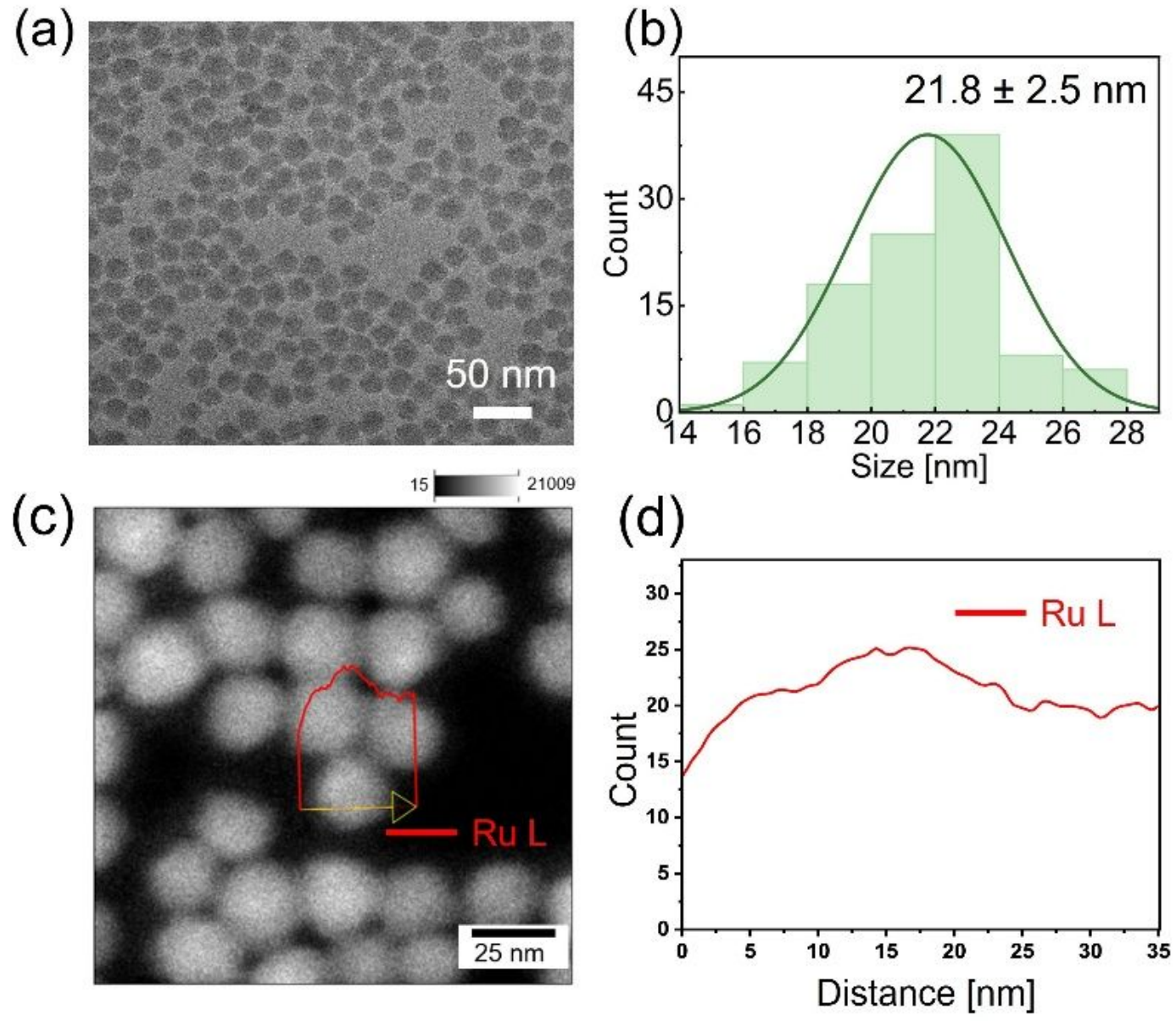

Figure S25. Morphology and elemental distribution of **Ruphen@ARGSiO$_2$** nanoparticles (**Ruphen** loading concentration: 1 μmol). (a) Representative TEM micrograph of **Ruphen@ARGSiO$_2$**. (b) Particle size distribution histogram obtained from TEM image analysis. (c) Image of a randomly selected **Ruphen@ARGSiO$_2$** particle with the EDX line-scan path indicated. (d) Corresponding Ru–EDX line-scan profile, made to confirm the presence of Ru across the particle.

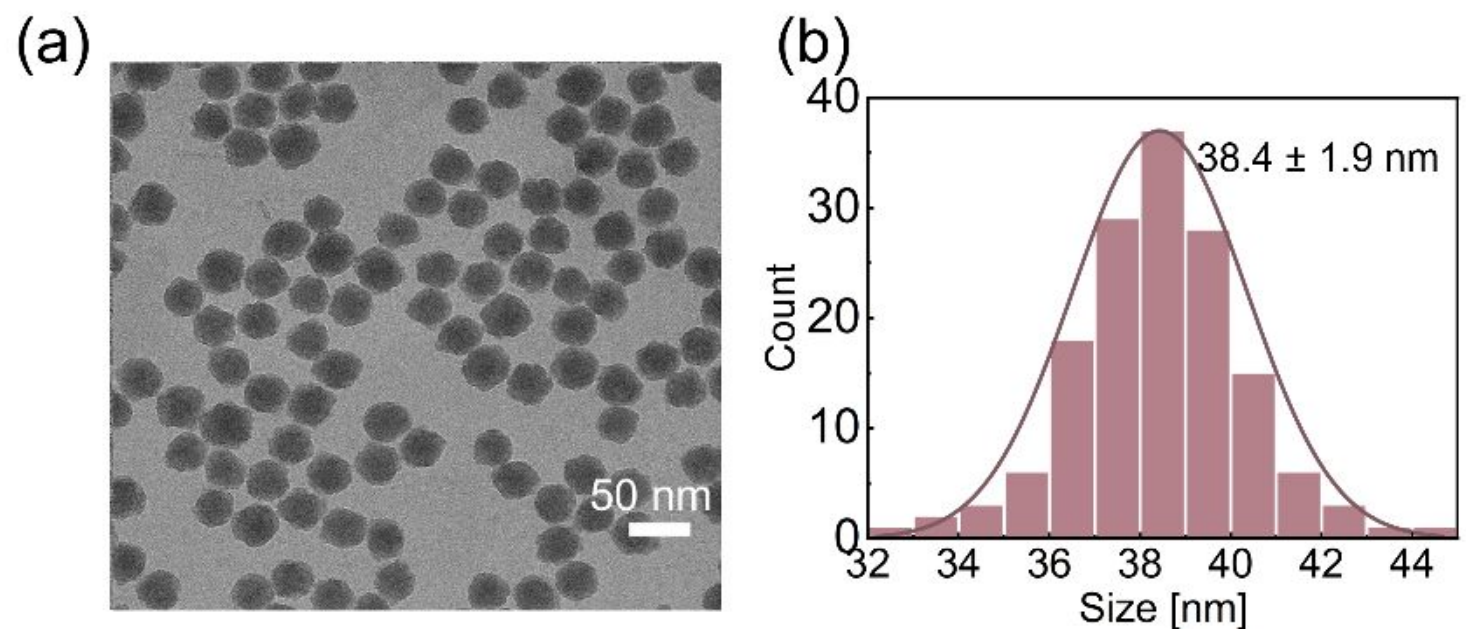

Figure S26. Morphology of unstained core–shell nanoparticles prepared by the L-arginine method (40 nm **US-ARGSiO$_2$**). (a) Representative TEM micrograph of the unstained core/unstained shell sample. (b) Particle size distribution histogram obtained from TEM image analysis.

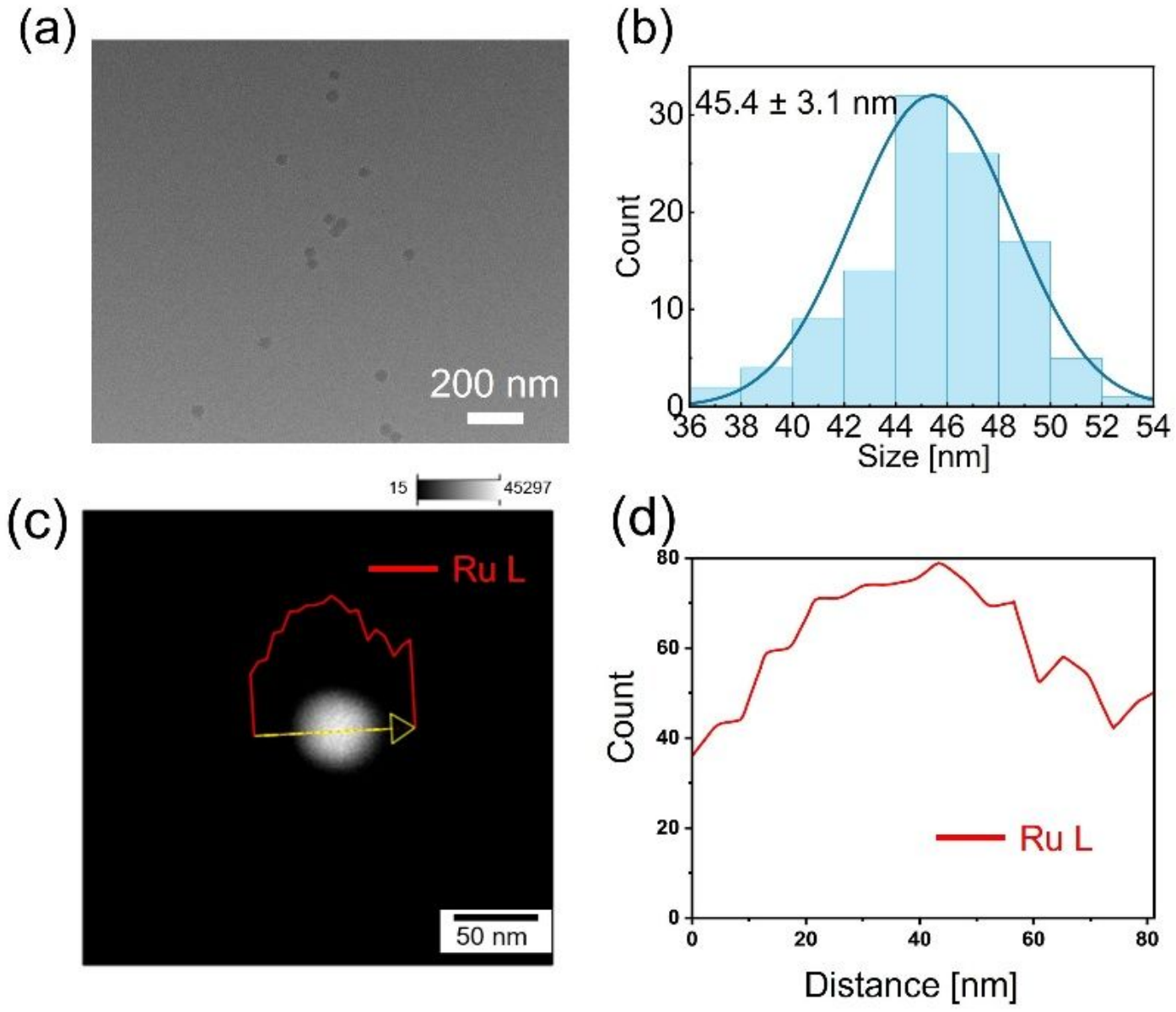

Figure S27. Morphology and elemental distribution of core–shell nanoparticles with an unstained core and stained with **Ruphenpy** shell (**(US-ARGSiO$_2$)@(Ruphen@ARGSiO$_2$)**, **Ruphenpy** loading concentration: 0.1 µmol) made by the L-arginine approach. (a) Representative TEM micrograph of the unstained-core/stained-shell sample. (b) Particle size distribution histogram obtained from TEM image analysis. (c) TEM image of a randomly selected particle with the EDX line-scan path indicated. (d) Corresponding EDX line-scan profile, confirming the localization of the stain element predominantly in the shell region.

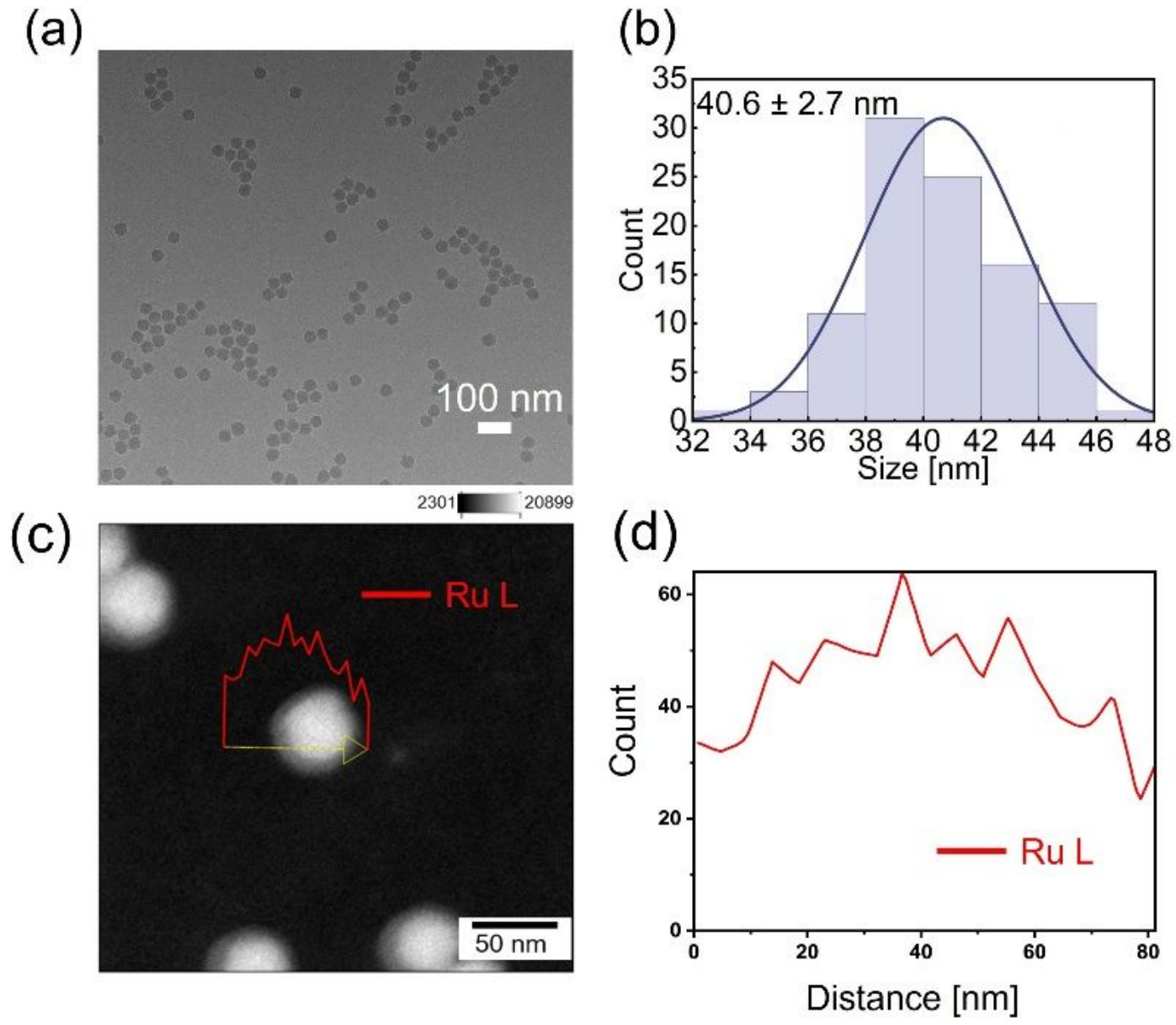

Figure S28. Morphology and elemental distribution of core–shell nanoparticles with a stained with **Ruphenpy** core and an unstained shell (**(Ruphen@ARGSiO$_2$)@(US-ARGSiO$_2$), Ruphenpy** loading concentration: 0.1 µmol) made by the L-arginine approach. (a) Representative TEM micrograph of the unstained-core/stained-shell sample. (b) Particle size distribution histogram obtained from TEM image analysis. (c) TEM image of a randomly selected particle with the EDX line-scan path indicated. (d) Corresponding EDX line-scan profile, confirming localization of the stain element predominantly in the shell region.

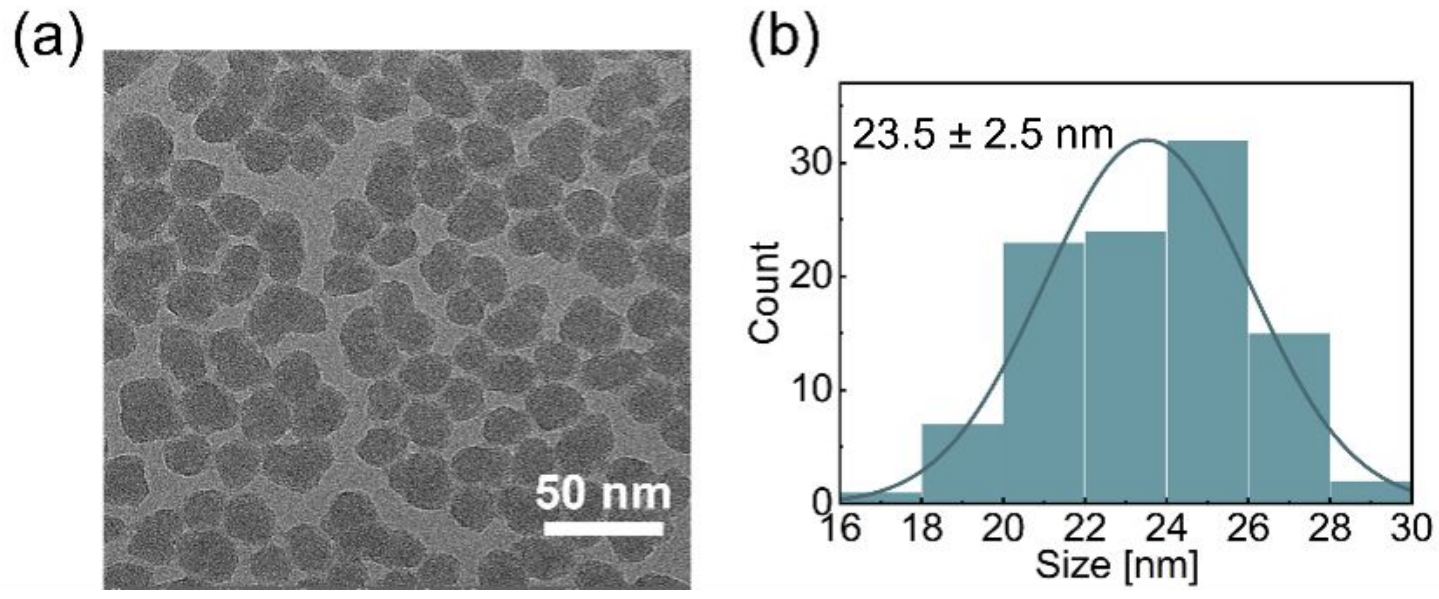

Figure S29. Morphology of **US-STOSiO$_2$** nanoparticles. (a) Representative TEM micrograph of **US-STOSiO$_2$**. (b) Particle size distribution histogram obtained from analysis of TEM images.

EDX measurements were conducted with a JEOL Centurio detector, which has excellent throughput and a high solid angle. For Cr detection, the strong Kα line at ~5.41 keV lies in the optimal energy range of the detector, providing high signal intensity, low background, and good peak separation from other elements. However, for Ru, the detection relies on the Lα line at ~2.56 keV, which is in a lower-energy region where the detection efficiency is reduced due to the higher absorption by the sample and potential spectral overlap with nearby elements, making an accurate quantification more challenging[10]

S3.8. Inductively coupled plasma mass spectrometry (ICP-MS)

ICP-MS determinations of Cr and Ru silica sample solutions were carried out using an ICP-QQQ-MS (Agilent 8900). Before ICP-MS measurements, excess complexes were removed by centrifugal filtration using Amicon Ultra centrifugal filters (include the company and the MWCO), and the corresponding unstained SiO_2 NPs were used as the reference/control for all comparisons. Sample solutions were prepared by adding approximately 40 mL of Milli-Q water to a 50 mL Falcon tube, followed by addition of 1.1 mL sub-boiled HNO_3 and 0.5 mL sub-boiled HCl. Then, 0.5 mL of the sample solution were pipetted into the Falcon tube, and the mixture was filled to 50 mL with Milli-Q water. Quantification was performed using external calibration; calibration solutions were prepared in the same acid matrix by adding the appropriate standard solution before filling to 50 mL. The isotopes ^{52}Cr and ^{102}Ru were measured using ^{89}Y (for Cr) and ^{103}Rh (for Ru) as internal standards, which were continuously added during measurement (2 ng/mL). The measurement uncertainty was estimated to 5 %.

Table S1. Parameters used for the ICP-MS measurements.

Measurement-Parameter	Ar-Cool flow : 15.0 L/min<br>Auxillary flow : 0.90 L/min<br>Nebulizer flow : 1.05 L/min<br>Plasma power : 1550 W<br>Nebulizer : Micromist S594688<br>Spray chamber : Scott
Analytes/ Isotopes	^{52}Cr, ^{103}Rh (internal Standard)<br>^{102}Ru, ^{103} Rh (internal Standard)

Table S2. Results of the ICP-MS measurements with selected Cr^{III}-stained and Ru^{II}-stained SiO_2 NPs.

	Sample	Cr or Ru amount from ICP-MS [ng/mL]	Particle mass in 1 mL after drying, [mg]	Efficiency of Cr or Ru complex incorporation [%]	Cr/SiO_2 or Ru/SiO_2 ratio [%]	Complexes per particle
Crddpd	**Crddpd@ARGSiO$_2$**	47 ± 10	5.80 ± 0.02	64 ± 14	0.0008 ± 0.0002	1.2 ± 0.4
	**Crddpd@STOSiO$_2$**	127 ± 26	2.40 ± 0.02	55 ± 11	0.0053 ± 0.0011	7.3 ± 3.2
Ruphenpy	**Ruphenpy@ARGSiO$_2$**	40 ± 8	6.60 ± 0.02	29 ± 6	0.0006 ± 0.0001	0.46 ± 0.19
	**Ruphenpy@STOSiO$_2$**	146 ± 30	2.60 ± 0.03	33 ± 7	0.0056 ± 0.0012	4.9 ± 2.0
	**(US-ARGSiO$_2$)@(Ruphenpy@ARGSiO$_2$**	154 ± 31	3.50 ± 0.02	76 ± 15	0.0044 ± 0.0009	26.6 ± 7.6
Ruphen	**Ruphen@ARGSiO$_2$**	34 ± 7	2.20 ± 0.02	19 ± 4	0.0015 ± 0.0003	2.3 ± 0.9

The efficiency of Cr^{III} and Ru^{II} SiO_2 NPs made by both sol-gel synthesis routes was calculated using the following equations, adapting the procedure reported in [9], exemplarily shown for **Crddpd@ARGSiO$_2$:**

MW(Crddpd) = 895.12 g/mol; A_r(Cr) = 51.996 g/mol; mCr found/mL = 47 ng/mL; $c_{particle}$ = 5.80 mg/mL; d_{TEM} = 23.1 nm; r = 11.55 nm; δ($ARGSiO_2$) = 2.08 g/cm³; $V_{Synthesis}$ = 79.1 mL; Added concentration of Crddpd = 0.1 .µmol.

$V_{sphere} = 4/3 \cdot \pi \cdot r^3 = 4/3 \cdot \pi \cdot (11.55E\text{-}9)^3 = 6.46E\text{-}24\ m^3 = 6.46E\text{-}18\ cm^3$

$m_{sphere} = \delta(SiO_2) \cdot V_{sphere} = 2.08\ g/cm^3 \cdot 6.46E\text{-}18\ cm^3 = 1.34E{-}17\ g$

$number_{particles/mL} = c_{particle} / m_{sphere}$ = 5.80E-3 g/mL / 1.34E−17 g = 4.32E14

$number_{particles/synthesis} = V_{Synthesis} \cdot c_{particle} / m_{sphere}$ = 79.1 mL·5.80E-3 g/mL / 1.34E-17 g = 3.41E16

$number_{complexes\_initial} = n_{initial}$(Crddpd)·6.022E23/mol = 1.12E-7 mol·6.022E23/mol = 6.73E16

$number_{max\ complexes/particle}$ = 6.73E16 / 3.41E16 = 1.70

$m_{complex\ found/mL}$ = mCr found/mL·MW(Crddpd) / M(Cr) = 47E-9 g·895.12 / 51.996 = 8.09E-7 g

$n_{complex\ found/mL}$ = 8.09E-7 g / 895.12 g/mol = 9.04E-10 mol

$n_{complex\ found/synthesis}$ = 9.04E-10 mol·79.1 = 7.15E-8 mol

$number_{complexes\_found/synthesis} = n_{complex\_found/synthesis}$·6.022E23/mol = 7.15E-8 mol·6.022E23/mol = 4.31E16

$number_{complexes\ found/particle}$ = 4.31E16 / 3.41E16 = 1.26 ≈ 1 complex per particle

Cr/SiO_2 ratio = 47E-6 mg/mL / 5.80 mg/mL·100% = 0.00081%

Incorporation efficiency = $n_{complex\_found/synthesis}$ / $n_{initial(Crddpd)}$·100% = 7.15E-8 mol / 1.12E-7 mol·100% = 64%

The uncertainties of the incorporation efficiencies and metal/SiO_2 ratios were estimated from the ICP-MS uncertainty and, where applicable, the particle mass concentration uncertainty. The uncertainties of the average number of complexes per particle were estimated by error propagation from the ICP-MS uncertainty, particle mass concentration uncertainty, and TEM-derived particle diameter uncertainty. The silica densities were treated as fixed values: 2.08 g/cm^{-3} for ARGSiO_2 and 2.20 g/cm^{-3} for STOSiO_2.

Table S3. Overview of the physicochemical particle properties.; n.d. = not determined; $d_{h,0}$ = number-based hydrodynamic diameter, d_{TEM} = particle diameter obtained from TEM measurements; PDI = polydispersity index.

Physicochemical particle properties					
Sample	d_{TEM} [nm]	$d_{h,0}$ [nm]	z-average [nm]	PDI [%]	ζ potential [mV]
**Ruphenpy-stained SiO2 NPs**					
**Ruphenpy@ARGSiO$_2$** (0.1 µmol)	22.4 ± 2.6	17 ± 4	25	9	-22.1 ± 1.2
**Ruphenpy@ARGSiO$_2$** (0.2 µmol)	21.6 ± 2.4	15 ± 4	26	14	-23.8 ± 2.5
**Ruphenpy@ARGSiO$_2$** (0.5 µmol)	18.5 ± 2.2	16 ± 4	26	15	-17 ± 3.2
**Ruphenpy@ARGSiO$_2$** (1 µmol)	19.5 ± 2.2	19 ± 4	29	11	-10.2 ± 3.4
**(Ruphen@ARGSiO$_2$)@(US-ARGSiO$_2$)** **(**0.1 µmol, stained core/unstained shell)	40.6 ± 2.7	33 ± 7	44	5	-27.5 ± 5.8
**(US-ARGSiO$_2$)@(Ruphen@ARGSiO$_2$)** **(**0.1 µmol, unstained core/stained shell)	45.4 ± 3.1	37 ± 8	48	4	-36.4 ± 2.0
**Ruphenpy@STOSiO$_2$** (0.1 µmol)	24.4 ± 2.9	20 ± 5	32	12	-36.3 ± 0.7
**Ruphen**-stained SiO$_2$ NPs					
**Ruphen@ARGSiO$_2$** (0.1 µmol)	25.7 ± 2.7	20 ± 5	29	9	-22.1 ± 1.2
**Ruphen@ARGSiO$_2$** (0.5 µmol)	22.4 ± 2.5	16 ± 4	30	19	-18.3 ± 0.7
**Ruphen@ARGSiO$_2$** (1 µmol)	21.8 ± 2.5	19 ± 5	27	11	-20.8 ± 1.2
**Crddpd**-stained SiO$_2$ NPs					
**Crddpd@ARGSiO$_2$** (0.1 µmol)	23.1 ± 2.5	17 ± 4	25.4	12	-23.8 ± 2.5
**Crddpd@STOSiO$_2$** (0.1 µmol)	22.2 ± 2.8	18 ± 4	33	16	-38.8 ± 1.2
Unstained SiO$_2$ NPs					
**US-ARGSiO$_2$** (25 nm)	23.6 ± 1.3	17 ± 4	25	9	-21.3 ± 3.9
**US-STOSiO$_2$**	23.5 ± 2.5	20 ± 5	30	9	-32.1 ± 4.4
**US-ARGSiO$_2$** (40 nm, core/shell)	38.4 ± 1.9	32 ± 7	42.7	4	-30.7 ± 2.3

S4. Optical properties
S4.1. Methods

Steady-state and time-resolved PL measurements

Photoluminescence (PL) spectra and PL decay kinetics were measured on calibrated spectrofluorometers (FSP920 and FLS980, Edinburgh Instruments). Steady-state emission spectra were recorded using a continuous xenon lamp as the excitation source. Time-resolved luminescence measurements on the FSP920 were performed with a microsecond xenon flashlamp and detected in multichannel scaling (MCS) mode. All measurements were carried out using aqueous dispersions in long neck quartz cuvette (10 mm x 10 mm, initially the neck of 10 cm was cut in order for the needle to reach the cuvette bottom; Hamamatsu) at room temperature unless stated otherwise. Excitation wavelengths of 435 nm and 450 nm were used for the Cr^{III}- and Ru^{II}-containing samples, respectively.

Two spectrofluorometers were employed for the time-resolved PL measurements to cover the different time ranges of the PLK decay kinetics of the quenched and O_2-shielded luminophores. Spectrofluorometer FLS980 was used for shorter decay times in the microsecond range, whereas spectrofluorometer FSP920 was used for longer decay times in the millisecond range. The latter instrument was, e.g., used for **Ruphenpy** embedded in silica nanoparticles resulting in a considerable increase in its PL lifetime/ PL decay kinetics, requiring the longer acquisition window provided by FSP920 to fully record the PL decay curves. Differences in excitation wavelength between setups are not expected to affect the decay behavior because excitation addresses the same electronic transition/manifold.

Sample preparation and O_2 removal

Air-saturated samples were measured under ambient air equilibrium (no bubbling). Argon-saturated samples were prepared by purging the sample solutions with Ar for 60 min at a constant flow rate through a rubber septum using an inlet needle and a vent needle to avoid pressure buildup. Samples were measured immediately after purging. Effective oxygen removal was verified by agreement of the post-purge decay kinetics with published lifetime values for the corresponding deoxygenated material. A purging time of 60 min was selected as sufficient for complete degassing while minimizing solvent evaporation.

Acquisition and correction of the PL spectra and data treatment

Slit widths (excitation/emission) were 6/4 nm for steady-state PL measurements and 8/4 nm for time-resolved measurements on FSP 920. The dwell time for steady-state spectra was 0.2 s per point, and the wavelength-dependent spectral responsivity of the spectrofluorometer was considered. The absorbance at the excitation wavelength was controlled to ensure comparable excitation conditions and to minimize inner-filter effects.

The spectrally corrected emission spectra were not corrected for scattering, as scattering contributions were negligible under the applied luminophore loading and measurement conditions. For visualization only, spectra were smoothed; all calculations/estimations were performed using the original (unsmoothed) spectra. **Crddpd**-containing samples' spectra were smoothed using an FFT-based (Fourier) filter, while **Ruphenpy** and **Ruphen** spectra were smoothed using LOESS (locally weighted regression) to preserve the original spectral shape.

For comparison of the spectral shapes, emission spectra were normalized by dividing all spectra by the global maximum intensity among the spectra being compared. For temperature-dependent experiments, measured relative (non-normalized) emission intensities were used to quantify intensity changes between cycles for identical instrument settings.

Time-resolved PL measurements

PL decay kinetics were collected at 775 nm for **Crddpd**-containing samples and 600 nm for **Ruphenpy** samples. The repetition rate was 100 Hz for a 2–10 ms time window and 50 Hz for a 20 ms time window. Measurements were stopped after collecting 10,000 photons for **Crddpd**-containing samples. For Ruphenpy samples, stopping conditions were adjusted depending on signal quality (typically 20,000 photons; up to 300,000 photons when required to improve the decay quality). For fitting/visualization, decay traces were normalized by dividing by the maximum count value.

For non-encapsulated **Ruphen** in water (air- and Ar-saturated atmospheres) and **Ruphenpy** in water under ambient air, time-resolved measurements were additionally performed on the spectrofluorometer FLS980 using an EPLD pulsed diode laser (λ_{ex} = 405 nm) as excitation light source.

For **Ruphen@ARGSiO$_2$** TCSPS (time-correlated single photon counting) measurements were carried out at 20 °C using a mini-τ setup from Edinburgh Instruments, equipped with an

Edinburgh Instruments TCC2 electronics and Edinburgh Instruments EPL 450 picosecond pulsed diode laser.

Temperature-dependent PL measurements

Temperature-dependent PL measurements were performed in a thermostated sample holder. The temperature was monitored using the sensor inside the sample holder and an external temperature controller; the temperature stability was ±0.5 °C. All samples were equilibrated for 12 min at each temperature prior to data acquisition.

The emission spectra were recorded from 5 to 85 °C in 5 °C steps during the heating cycles unless stated otherwise. For **Crddpd@ARGSiO$_2$**, the time-resolved PL first temperature series (5–85 °C in 5 °C steps) required nearly 4 d of measurement time; therefore, subsequent measurements used 5 °C steps at the beginning and end (5–10 °C and 85 °C) and 10 °C steps in the intermediate range (20–80 °C) to reduce the total acquisition time while maintaining a good data comparability at the endpoints.

Temperature-cycling experiments were performed in triplicate using two approaches: (i) three consecutive cycles measured with the same cuvette sample, with a maximum of 20 min between cycles after returning to 5 °C, followed by re-equilibration at 5 °C before starting the next cycle; and (ii) three independent measurements of the first cycle using three separately prepared aliquots from the same sample batch.

All O_2-shielding experiments were performed in triplicate.

PL decay analysis and determination of PL lifetimes

The instrument response functions (IRF) were recorded using a scattering Ludox solution, and reconvolution was applied during fitting. The fit quality was evaluated using χ^2 and inspection of residuals. The fitting parameters/models were selected to improve residuals while avoiding unnecessary over-parameterization.

For the **Crddpd**-containing samples in water (air/Ar), the decay kinetics were fitted using a mono-exponential reconvolution model (IRF included, FAST v341 software). For **Crddpd** embedded in silica nanoparticles, bi-exponential or tri-exponential reconvolution fits were used. The models were selected to achieve a good quality of the fits (χ^2<1.2).

For **Ruphenpy** in the non-encapsulated state in water, the decays were fitted using a mono-exponential model (FAST v341 software). For **Ruphenpy** incorporated in silica nanoparticles, the decays were complex and multi-exponential; therefore, the PL lifetimes were evaluated using two approaches: (i) an integral (area-under-the-decay curve) lifetime and (ii) a four-exponential reconvolution fit (IRF included). The four-exponential components were used as a phenomenological description to calculate an average lifetime with no physical meaning assigned to the respective components.

For multi-exponential fits, the intensity-weighted average lifetime was calculated as:

$$\langle \tau \rangle_{int} = \frac{\sum_{i=1}^{n} B_i \tau_i^2}{\sum_{i=1}^{n} B_i \tau_i} \tag{1}$$

Quantification of the O_2 shielding efficiency

The oxygen shielding efficiency of the different silica nanoparticles was quantified by comparing luminescence signals measured for the same samples under ambient air and after purging with argon (Ar-saturated dispersions), with the latter serving as benchmark values for the respective non-quenched luminophore in a deoxygenated environment. Based on steady-state PL intensity measurements, the shielding efficiency was calculated as:

$$Steady-state\ O_2\text{-}shielding\ efficiency\ [\%] = \frac{I_{air}}{I_{Ar}} \times 100 \tag{2}$$

In addition, we used time-resolved PL decay measurements to quantify the O_2 shielding efficiency because luminescence lifetimes are highly sensitive to environmental changes including collisional quenching by O_2 while being independent of excitation power density fluctuations and luminophore concentration. Accordingly, the O_2 shielding efficiency derived from the intensity-weighted average lifetime was calculated as

$$Decay-time-based\ O_2-shielding\ efficiency\ [\%] = \frac{\tau_{avg\_air}}{\tau_{avg\_Ar}} \times 100 \tag{3}$$

However, for multi-exponential **Ruphenpy@ARGSiO$_2$** and **Ruphenpy@STOSiO$_2$** decay, an additional lifetime-based metric was introduced that does not require consideration of absorption factors at the excitation wavelength: for selected samples, the PL decay curves were recorded under air- and argon-saturated conditions using identical photon-counting acquisition

times, the integrated areas under the PL decay curves were determined, and the O_2 shielding efficiency was calculated as

$$Decay{-}area{-}based\ O_2{-}shielding\ efficiency\,[\%] = \frac{A_{air}}{A_{Ar}} \times 100 \tag{4}$$

where A denotes the integrated decay area.

We now explicitly state that these values (steady-state O_2-shielding efficiency, decay-time-based O_2-shielding efficiency, decay-area-based O_2-shielding efficiency) are not expected to be numerically identical, because they are derived from different experimental observables and each method has its own experimental uncertainty. Steady-state spectra represent spectrally integrated emission intensities and can be affected by baseline correction, spectral integration limits, and intensity fluctuations, whereas decay curves are obtained using pulsed excitation and time-resolved detection at selected emission wavelengths/detection windows and depend on photon statistics as well as fitting or integration procedures. In addition, Ruphenpy exhibits multiexponential decay kinetics in the silica matrix. These factors can explain why the steady-state intensity-based value and the decay-area-based value are not identical.

For the comparison of free and nanoparticle-encapsulated luminophores, we considered whether absolute steady-state PL intensities could be compared at identical complex concentrations. However, such a comparison is experimentally not straightforward for the present nanoparticle dispersions. In particular, for the silica-encapsulated **Ruphen** and **Ruphenpy**, the absorption bands of the embedded complex are strongly obscured by light scattering from the SiO_2 nanoparticles. Therefore, reliable absorbance matching, or correction for the actually absorbed excitation light, is not possible. Although the average complex concentration in the nanoparticle dispersion can be estimated from ICP-MS data, equal nominal complex concentrations would not ensure equal excitation conditions because scattering, excitation attenuation, and the different local environment of the encapsulated complexes affect the measured PL intensity.

For this reason, we did not use absolute intensity comparisons between free and encapsulated complexes as quantitative evidence. Instead, we performed time-resolved PL decay measurements, because luminescence decay times are largely independent of luminophore concentration, excitation intensity, and scattering-related intensity losses. This allowed us to compare the photophysical behavior of free and encapsulated complexes more reliably than by absolute steady-state intensities.

Calculation of the energy gap ΔE

The energy gap between two thermally coupled emitting states was obtained from a Boltzmann analysis of the integrated emission intensity ratio.

$$\ln\left(\frac{I_2}{I_1}\right) = \ln C - \frac{\Delta E}{k_B} \cdot \frac{1}{T} \quad (5)$$

$$\Delta E = k_B T \left[\ln C - \ln\left(\frac{I_2}{I_1}\right)\right] \quad (6)$$

Plotting $\ln(I_2/I_1)$ versus 1/T gives a straight line:

$$Slope\ m = -\frac{\Delta E}{k_B} \quad (7)$$

$$\boldsymbol{\Delta E = -m k_B} \quad (8)$$

where k_B is the Boltzmann constant.

S4.2 Oxygen sensitivity of Ruphenpy-loaded L-arginine SiO_2 core@shell nanoparticles

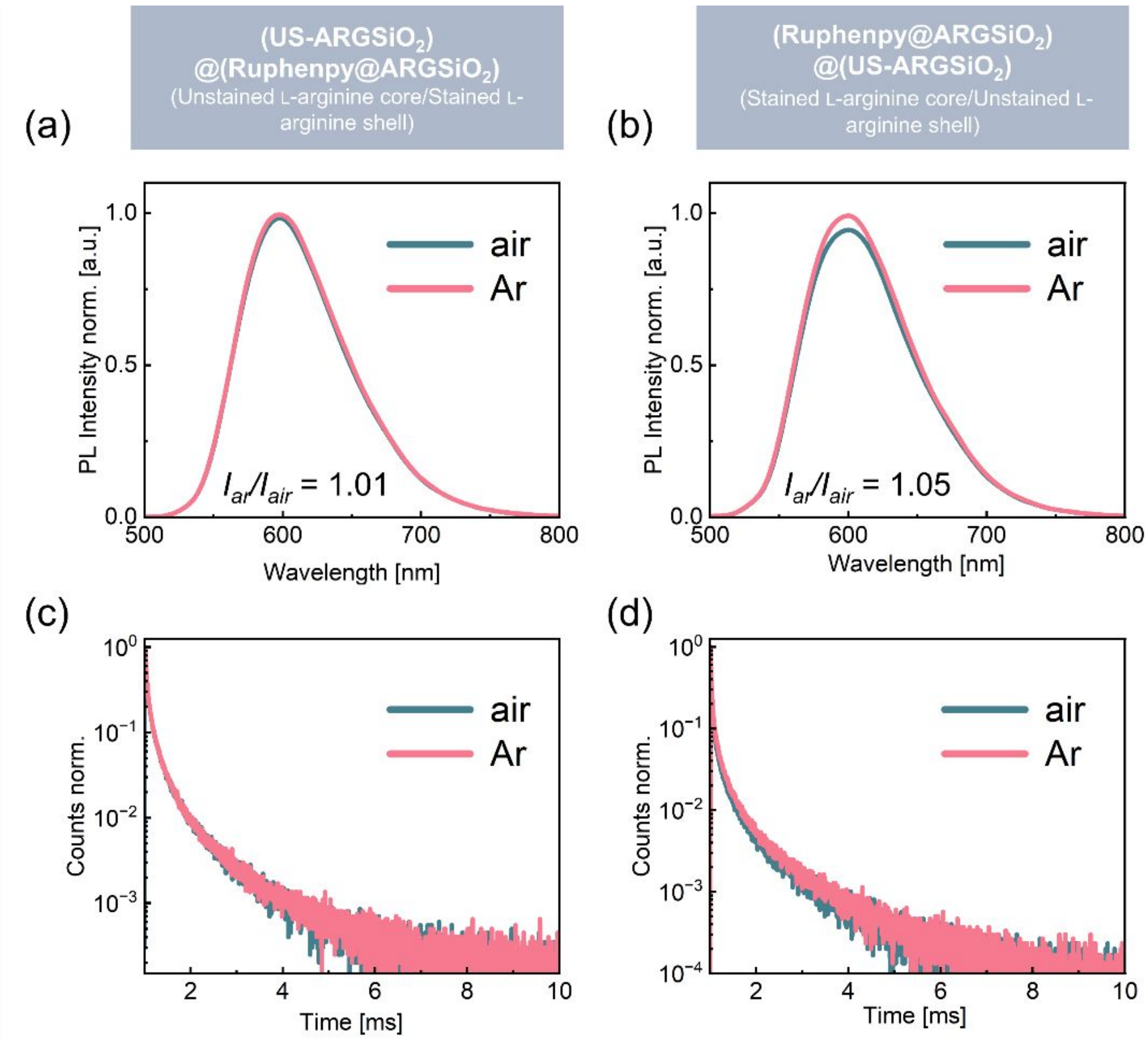

Figure S30. (a) Normalized emission spectra of unstained core SiO_2 NPs and SiO_2 NPs covered with a **Ruphenpy** shell (**(US-ARGSiO$_2$)@(Ruphenpy@ARGSiO$_2$)**) made by the L-arginine approach using a **Ruphenpy** loading concentration of 0.1 µmol under atmospheric (air, dark green) and anaerobic conditions (Ar, salmon pink); (b) Normalized emission spectra of core@shell nanoparticles with a **Ruphenpy-**stained core and an unstained shell (**(Ruphenpy@ARGSiO$_2$)@(US-ARGSiO$_2$)**) made by the L-arginine approach employing a **Ruphenpy** loading concentration of 0.1 µmol under atmospheric (air, dark green) and anaerobic conditions (Ar, salmon pink). (c) Corresponding normalized PL decay kinetics of (**US-ARGSiO$_2$)@(Ruphenpy@ARGSiO$_2$)** in air- and argon-saturated dispersion; (d) Corresponding normalized PL decay kinetics of (**Ruphenpy@ARGSiO$_2$)@(US-ARGSiO$_2$).**

S4.3 Oxygen sensitivity of Ruphen-loaded L-arginine SiO_2 nanoparticles

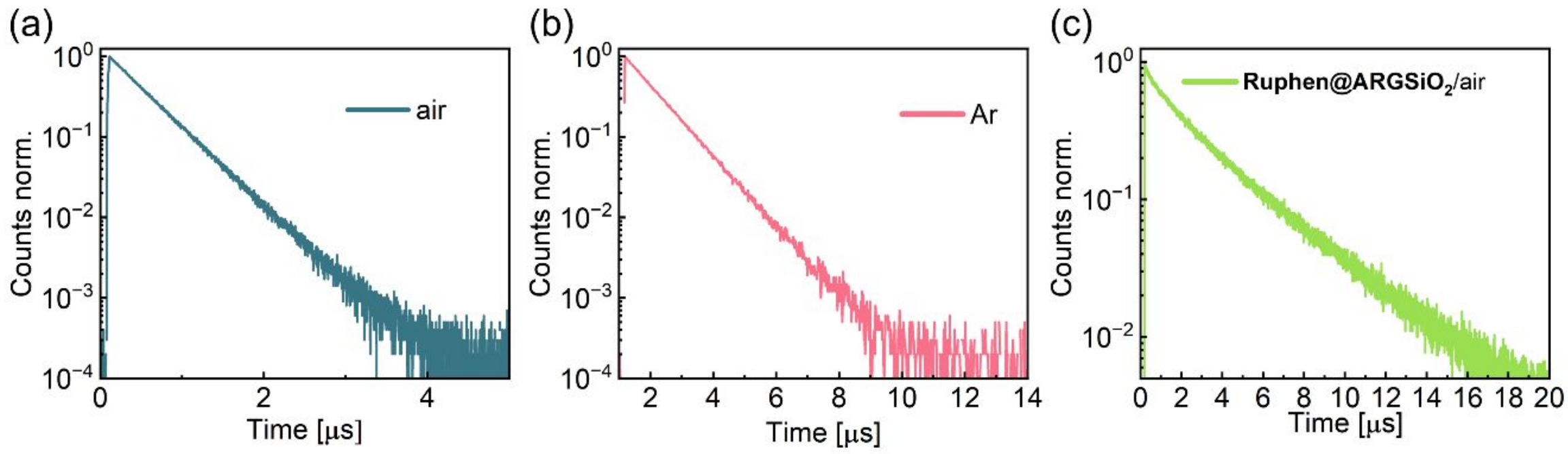

Figure S31. Luminescence decay kinetics of **Ruphen** (a) in water under atmospheric and (b) anaerobic conditions and encapsulated in (c) L-arginine-type SiO_2 NPs in air-saturated solution.

S4.4 Photostability studies

S.4.41 Experimental setup and sample preparation

Solutions of the target complexes were protected from light during synthesis and purification, and the isolated products were stored in the dark. For preparation of the homogeneously dissolved sensitizer solutions, 4 mL of water was used, yielding 3 μM solutions of **Ruphen** and **Ruphenpy**, respectively, which were subsequently transferred into cuvettes.

Stability measurements were performed using a continuous-wave (cw) external laser operating at 447 nm (Roithner Lasertechnik, RLTMDL-447-1W-3) with an output power adjusted to 0.5 W. The excitation beam was expanded using a 5x beam expander (Thorlabs, GBE05-A) to ensure homogeneous sample illumination with an excitation power density of ~0.5 W cm^{-2}. The illumination setup is shown in Figure S32. Samples were continuously irradiated for 110 min, during which the emission intensity at the spectral maximum was recorded using a PerkinElmer FL-6500 fluorescence spectrometer. To suppress scattered excitation light and enable accurate emission detection, a 495 nm long-pass filter (Newport, 20CGA-495) was placed in front of the detector.

Before and after illumination, the UV/vis absorption spectra were recorded using a JASCO V-670 spectrophotometer, and emission spectra were measured via cw laser excitation using the same setup with the PerkinElmer FL-6500 fluorescence spectrometer.

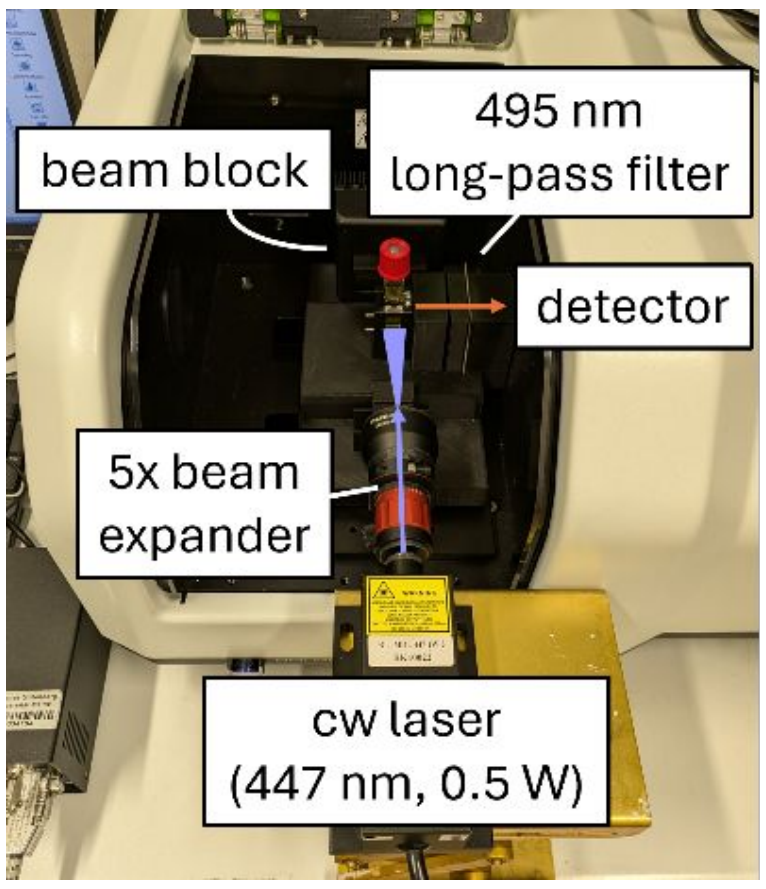

Figure S32. Experimental setup used for the photostability measurements of the Ru^{II} compounds utilizing the emission of the complexes, featuring an external 447 nm cw laser (0.5 W) for excitation, a beam expander for homogeneous sample illumination, and a 495 nm long-pass filter to suppress scattered excitation light.

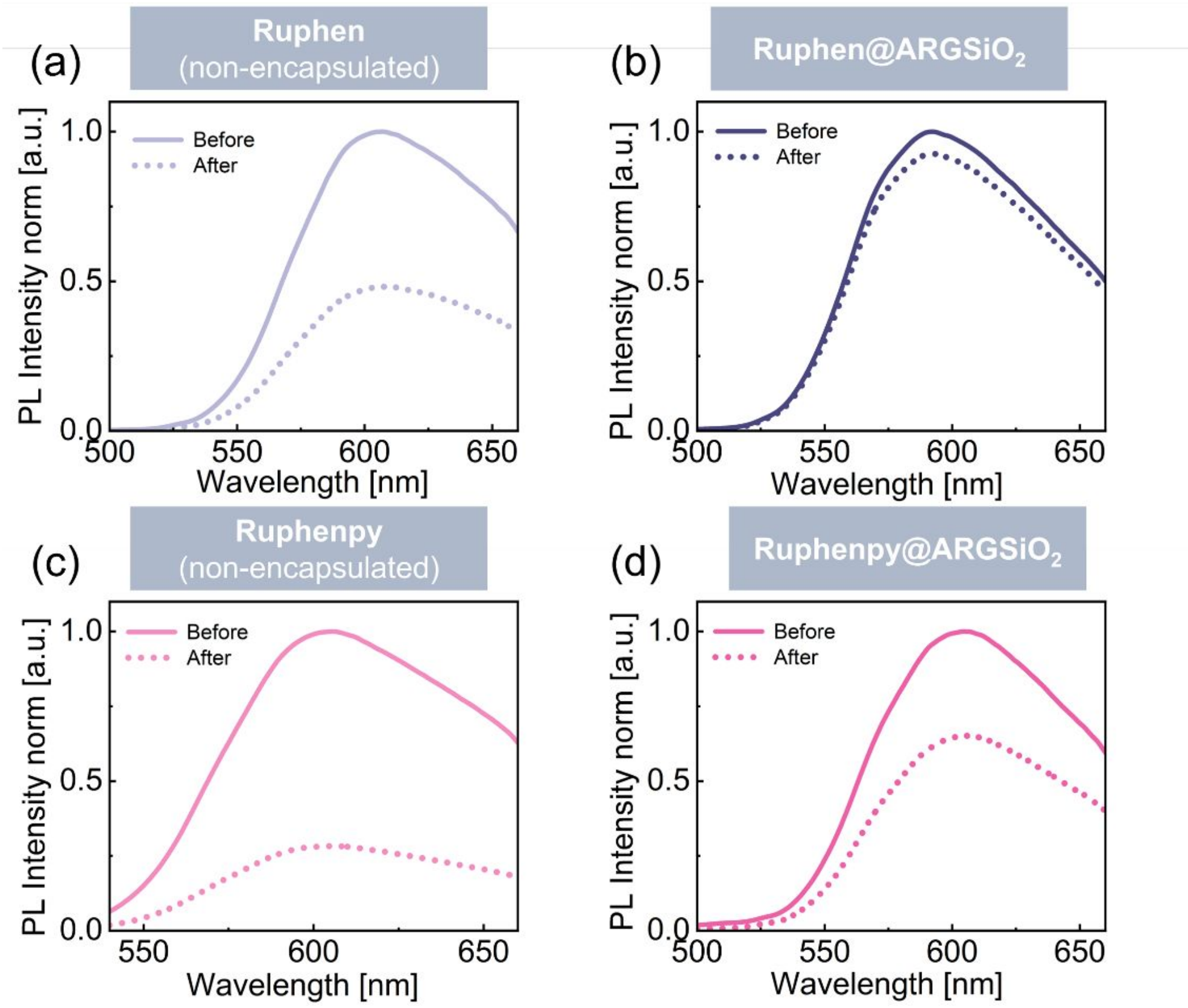

Figure S33. Photoluminescence stability measurements of non-encapsulated (3 µM) and L-arginine SiO_2-encapsulated **Ruphen** and **Ruphenpy** complexes (both as chloride salts) in **Ar-saturated** aqueous solutions under cw laser excitation at 447 nm (0.5 W). Shown are the emission spectra recorded before (straight line) and after (dotted line) illumination of (a) non-encapsulated **Ruphen**, (b) **Ruphen@ARGSiO$_2$**, (c) non-encapsulated **Ruphenpy** and (d) **Ruphenpy@ARGSiO$_2$**.

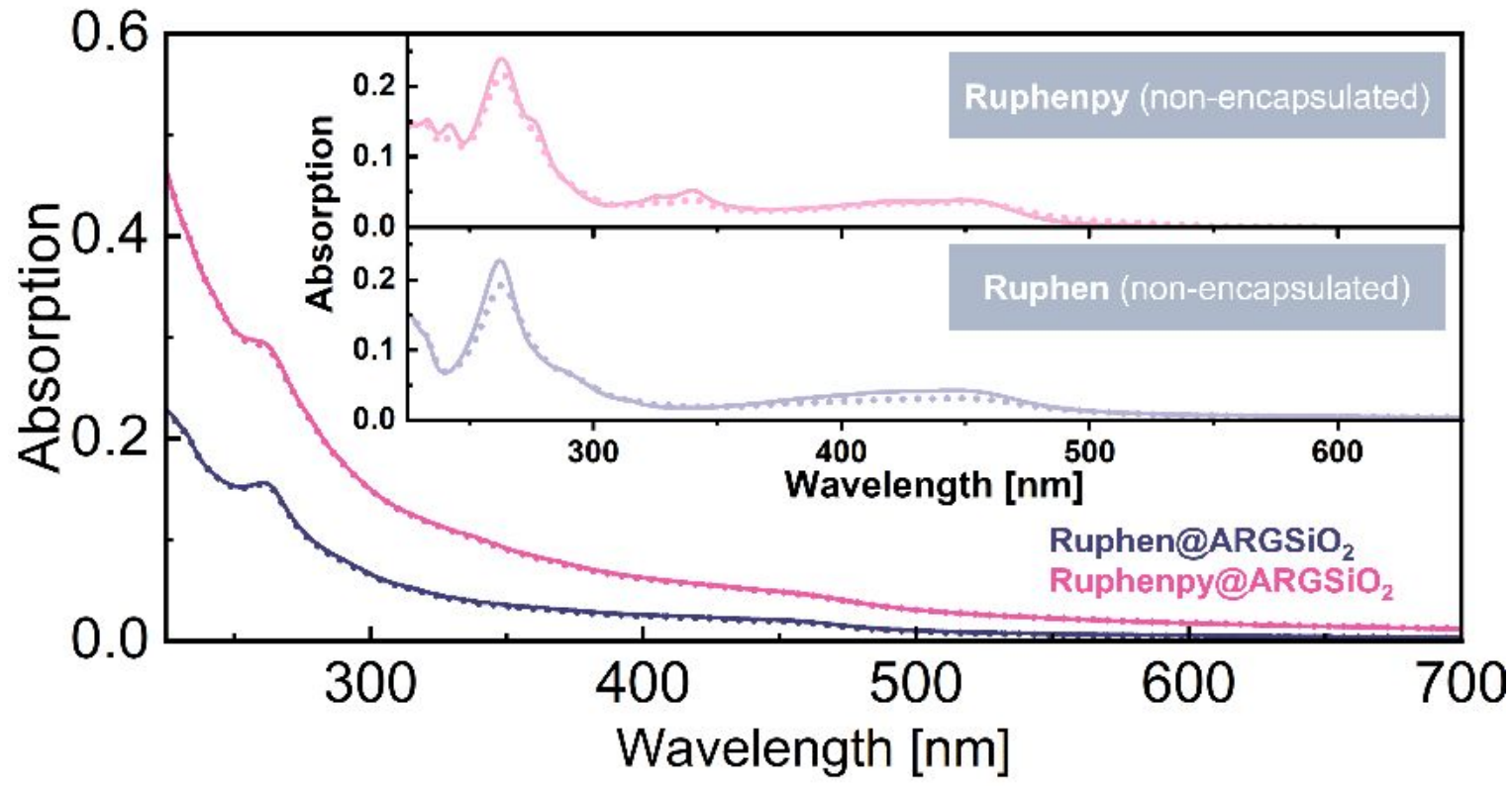

Figure S34. UV/vis spectra of **Ruphen** and **Ruphenpy** in **Ar-saturated** water, shown for both non-encapsulated solutions (muted purple and muted pink, inset) and L-arginine SiO_2-encapsulated samples - **Ruphen@ARGSiO$_2$** and **Ruphenpy@ARGSiO$_2$** (bright purple and bright pink, main part of the figure), recorded before (straight lines) and after 110 min of irradiation (dotted lines). Corresponding emission data sets are shown in Figure S33. The non-zero baseline in the red wavelength range arises from scattering of the silica nanoparticles.

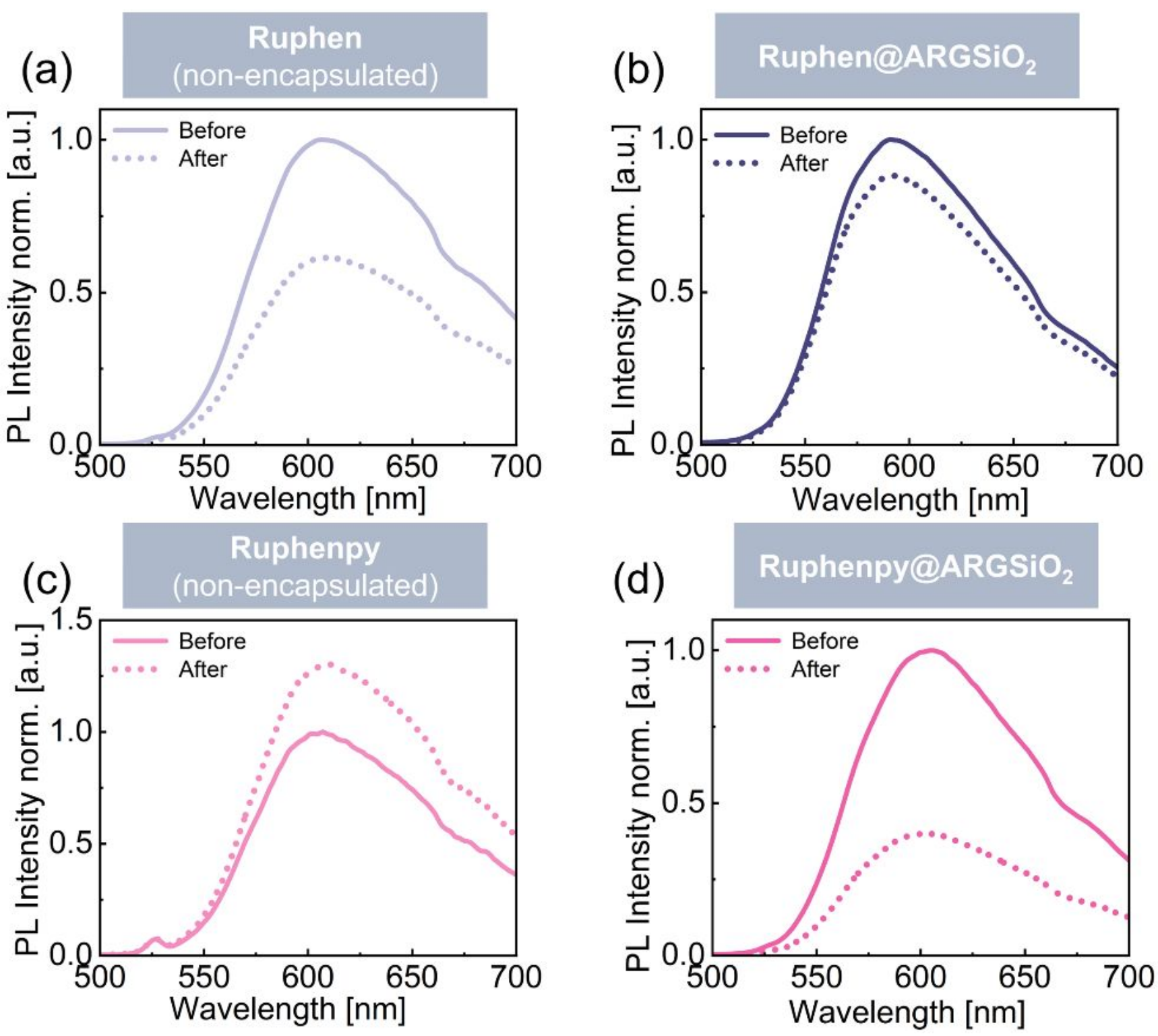

Figure S35. Photoluminescence stability measurements of non-encapsulated (3 µM) and L-arginine SiO_2-encapsulated **Ruphen** and **Ruphenpy** complexes (as chloride salts) in **air-saturated** aqueous dispersion under cw laser excitation at 447 nm (0.5 W). Shown are the emission spectra recorded before (straight line) and after (dotted line) illumination of (a) non-encapsulated **Ruphen**, (b) **Ruphen@ARGSiO$_2$**, (c) non-encapsulated **Ruphenpy** and (d) **Ruphenpy@ARGSiO$_2$**.

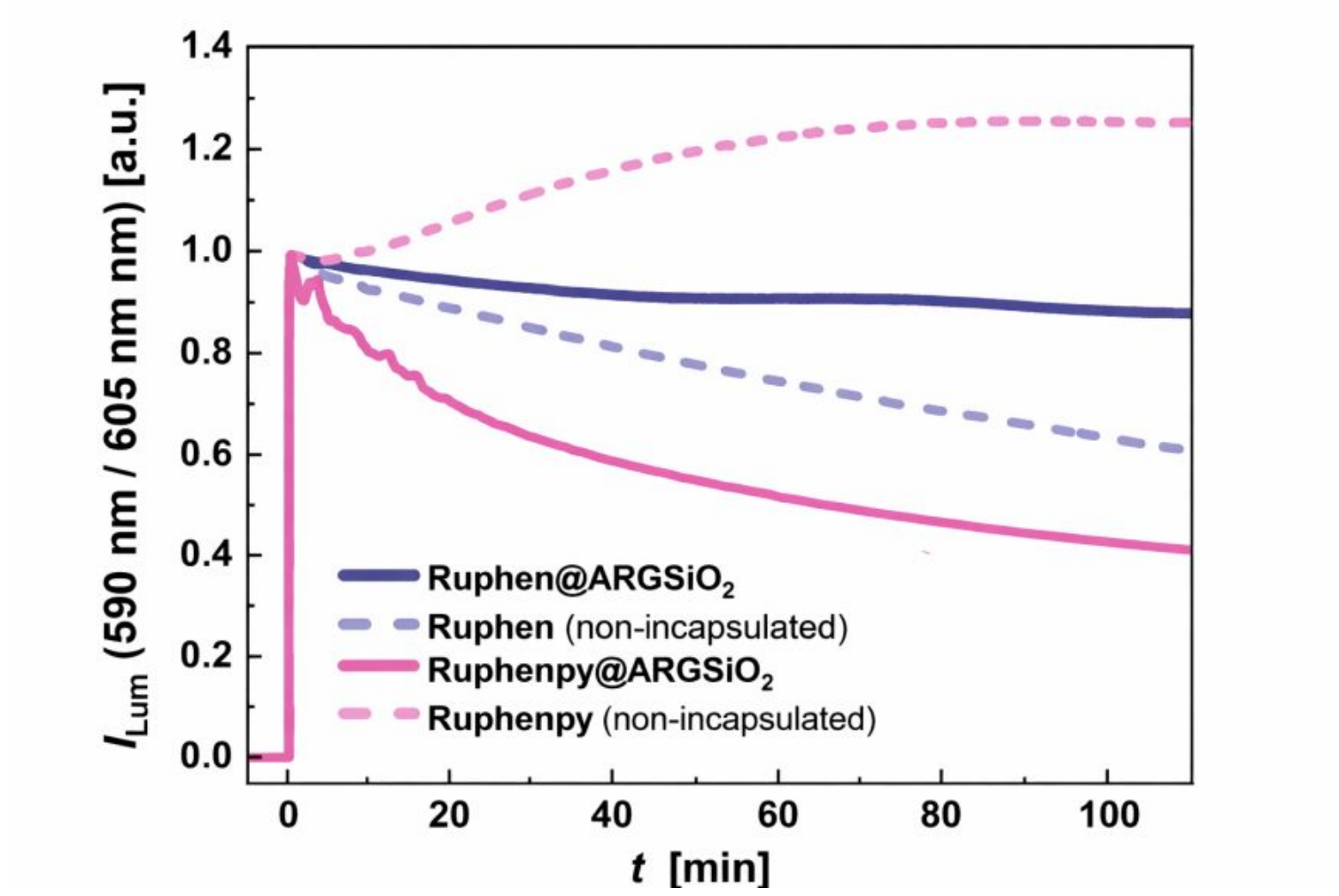

Figure S36. Photoluminescence stability measurements of non-encapsulated (3 µM) and L-arginine SiO_2-encapsulated **Ruphen** and **Ruphenpy** complexes in **air-saturated** aqueous solutions under cw laser excitation at 447 nm (0.5 W) using the setup shown in Figure S32. The temporal evolution of the normalised emission intensity at 590 nm for **Ruphen@ARGSiO₂** and at 605 nm for non-encapsulated **Ruphen**, **Ruphenpy**, and silica encapsulated **Ruphenpy@ARGSiO₂** dispersions during illumination.

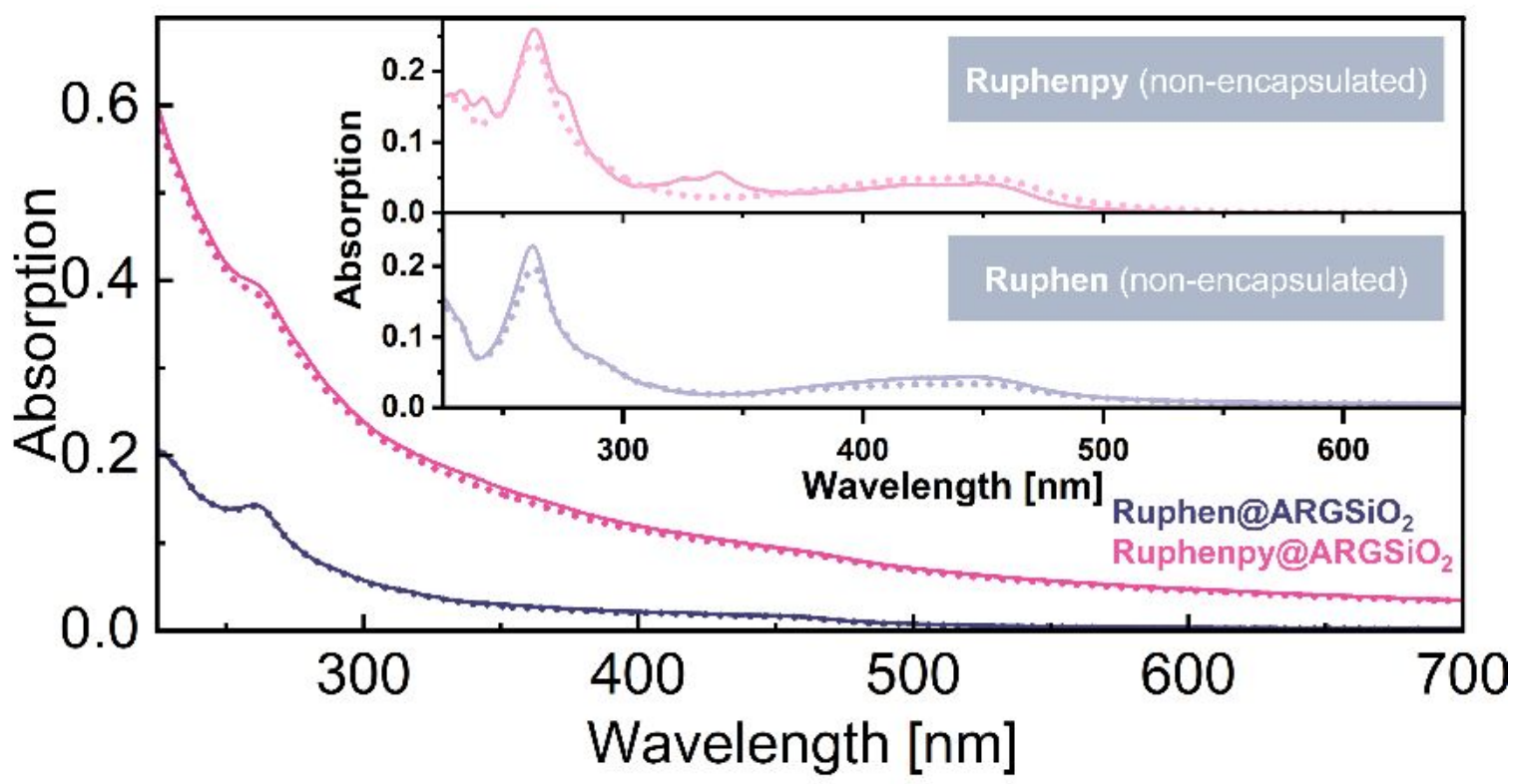

Figure S37. UV/vis spectra of **Ruphen** and **Ruphenpy** in **air-saturated** water, shown for both non-encapsulated solutions (muted purple and muted pink, inset) and L-arginine SiO_2 NP-encapsulated samples **Ruphen@ARGSiO₂** and **Ruphenpy@ARGSiO₂** (bright purple and bright pink, main part of figure), recorded before (straight lines) and after 110 min of illumination (dotted lines). Corresponding emission data sets are shown in Figure S35. The non-zero baseline in the red wavelength range arises from scattering of the silica nanoparticles.

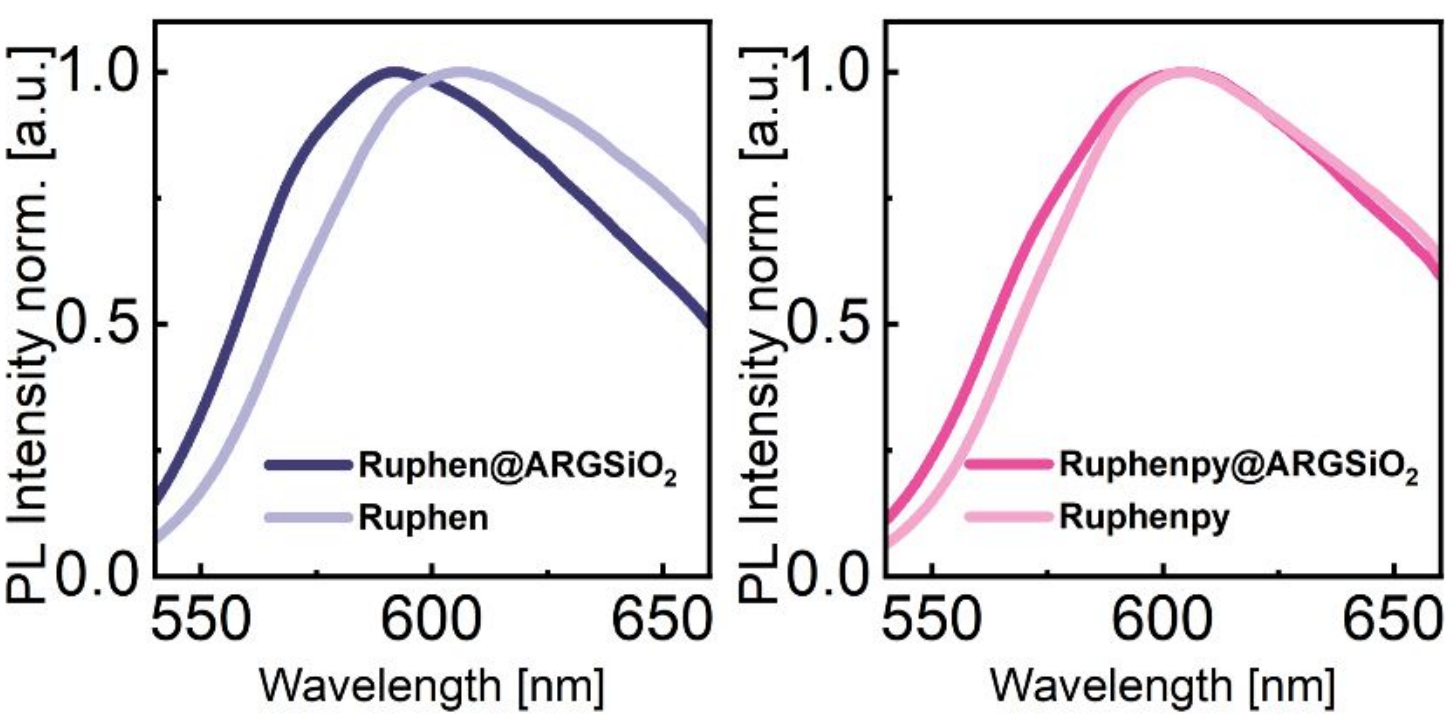

Figure S38. Emission spectra of non-encapsulated **Ruphen** (muted purple) and **Ruphenpy** (muted pink) in **Ar-saturated** water and the L-arginine SiO_2-encapsulated samples **Ruphen@ARGSiO₂** (dark purple) and **Ruphenpy@ARGSiO₂** (dark pink); excitation with a 447 nm cw laser (experimental setup, see Figure S32).

S5. Temperature sensing studies

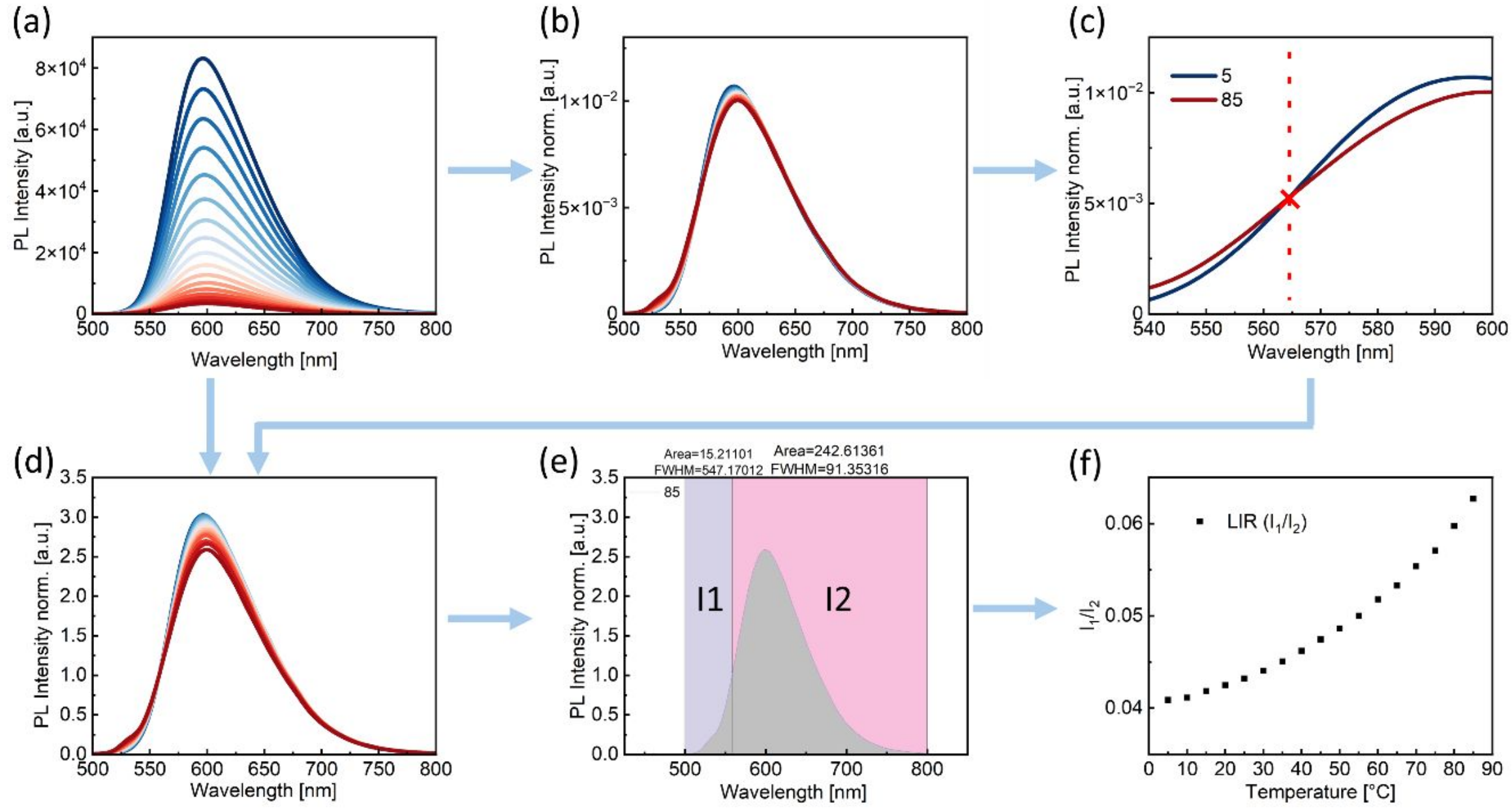

Figure S39. Scheme of the pre-processing of temperature-dependent luminescence data for **Ruphen** and **Ruphenpy** samples (example: non-encapsulated **Ruphen** in Ar-saturated water).

For Ruphenpy, a pseudo-ratiometric spectral-window analysis was applied. It should be noted that Ruphenpy is a broad single-band emitter; therefore, this analysis does not have the same physical basis as a true ratiometric thermometer based on two independent emission bands or two thermally coupled emissive states. Instead, the approach is used as an empirical self-referenced spectral-shape analysis. It was inspired by recently reported single-band ratiometric nanothermometry concepts, where one broad emission band is divided into two spectral regions around an isothermal or near-isothermal crossing region.

A heating cycle from 5 to 85 °C was recorded as a series of emission spectra (Figure S39a). For each spectrum, the integrated emission area was calculated over 500–800 nm, and the spectra were normalized to their respective integrated areas to visualize temperature-induced changes in spectral shape (Figure S39b). Next, the apparent crossing wavelengths of spectra recorded at different temperatures were determined. For this purpose, intersections between spectra from the series were considered, for example, intersections of the 5 °C spectrum with spectra recorded at 10, 15, 20 °C and so on up to 85 °C. An example intersection is highlighted in Figure S39c.

The apparent crossing wavelength was not strictly fixed, but remained confined to a relatively narrow spectral region during repeated heating cycles. For Ruphenpy in Ar-saturated aqueous solution, for example, the crossing wavelength varied from approximately 555–558 nm at low temperature to approximately 564–566 nm at high temperature, corresponding to a total shift of about 10 nm over the investigated temperature range. Therefore, this feature is referred to as a near-isothermal crossing region rather than a fixed isothermal point.

After collecting all crossing wavelengths, their mean value was calculated and used as the boundary between the two spectral regions. The original raw spectra were then normalized to a common reference intensity at this mean crossing wavelength (Figure S39d). Two integration windows were defined from 500 nm to the mean crossing wavelength, I_1, and from the mean crossing wavelength to 800 nm, I_2 (Figure S39e). For each temperature, I_1 and I_2 were calculated, and the pseudo-ratiometric spectral-window ratio was obtained as: $\mathrm{LIR} = \mathrm{I_1}/\mathrm{I_2}$.

The resulting LIR values are plotted (scatter plot, Figure S39(f)) as a function of temperature and fitted with a Boltzmann-type function. The relative sensitivity S_r is then calculated from the fitted LIR–T dependence using the formula:

$$S_r = \frac{1}{LIR}\frac{\delta LIR}{\delta T} \tag{9}$$

The thermal resolution or temperature uncertainty δT is calculated using this formula:

$$\frac{\delta LIR}{LIR} = \sqrt{\left(\frac{\delta I_1}{I_1}\right)^2 + \left(\frac{\delta I_2}{I_2}\right)^2} \tag{10}$$

Please note, that Ruphenpy is a broad single-band emitter and that the applied spectral-window ratio does not have the same physical basis as a true ratiometric thermometer based on two independent emission bands or two thermally coupled emissive states. From now refer to this analysis as a pseudo-ratiometric spectral-window analysis rather than a true LIR-based ratiometric thermometry approach.

This approach was inspired by recently reported single-band ratiometric nanothermometry concepts, where one broad emission band is divided into two spectral regions around an isothermal or near-isothermal crossing region [11]. In our case, the ratio reflects temperature-induced spectral redistribution within the broad Ruphenpy emission band rather than the population ratio of two distinct emissive states.

The apparent crossing wavelength is not strictly fixed but remains confined to a relatively narrow spectral range during repeated heating cycles. For **Ruphenpy** in Ar-saturated aqueous solution, for example, the crossing wavelength varies from approximately 555–558 nm at low temperature to approximately 564–566 nm at high temperature, corresponding to a total shift of about 10 nm over the investigated temperature range. Therefore, we now describe this feature as a near-isothermal crossing region rather than a fixed isothermal point.

The physical origin of the spectral redistribution is attributed to temperature-induced changes in the shape and position of the broad **Ruphenpy** emission band, most likely related to changes in local environment, solvent/matrix interactions, and the relative contribution of emissive configurations of the Ru^{II}–pyrene dyad. We do not assign this behavior to two thermally coupled emissive levels. Accordingly, the pseudo-ratiometric analysis is used as an empirical self-referenced spectral-shape parameter to follow temperature-dependent changes, and its limitations compared with true ratiometric thermometry are now explicitly stated.

For the **Crddpd**-containing samples, all emission spectra were normalized to the intensity at the maximum of the first emission band (738 nm). The luminescence intensity ratio (LIR) was then calculated from two integrated regions: I_1, obtained by integrating the emission from 700 to 745 nm, and I_2, obtained by integrating from 745 to 830 nm. After LIR determination, all subsequent processing steps (LIR–temperature plotting, fitting with the Boltzmann-type function, and calculation of the relative sensitivity S_r and temperature uncertainty δT) were performed analogously to the **Ruphen**/**Ruphenpy** temperature-dependent data.

S5.1 Crddpd: luminescence intensity-based temperature sensing

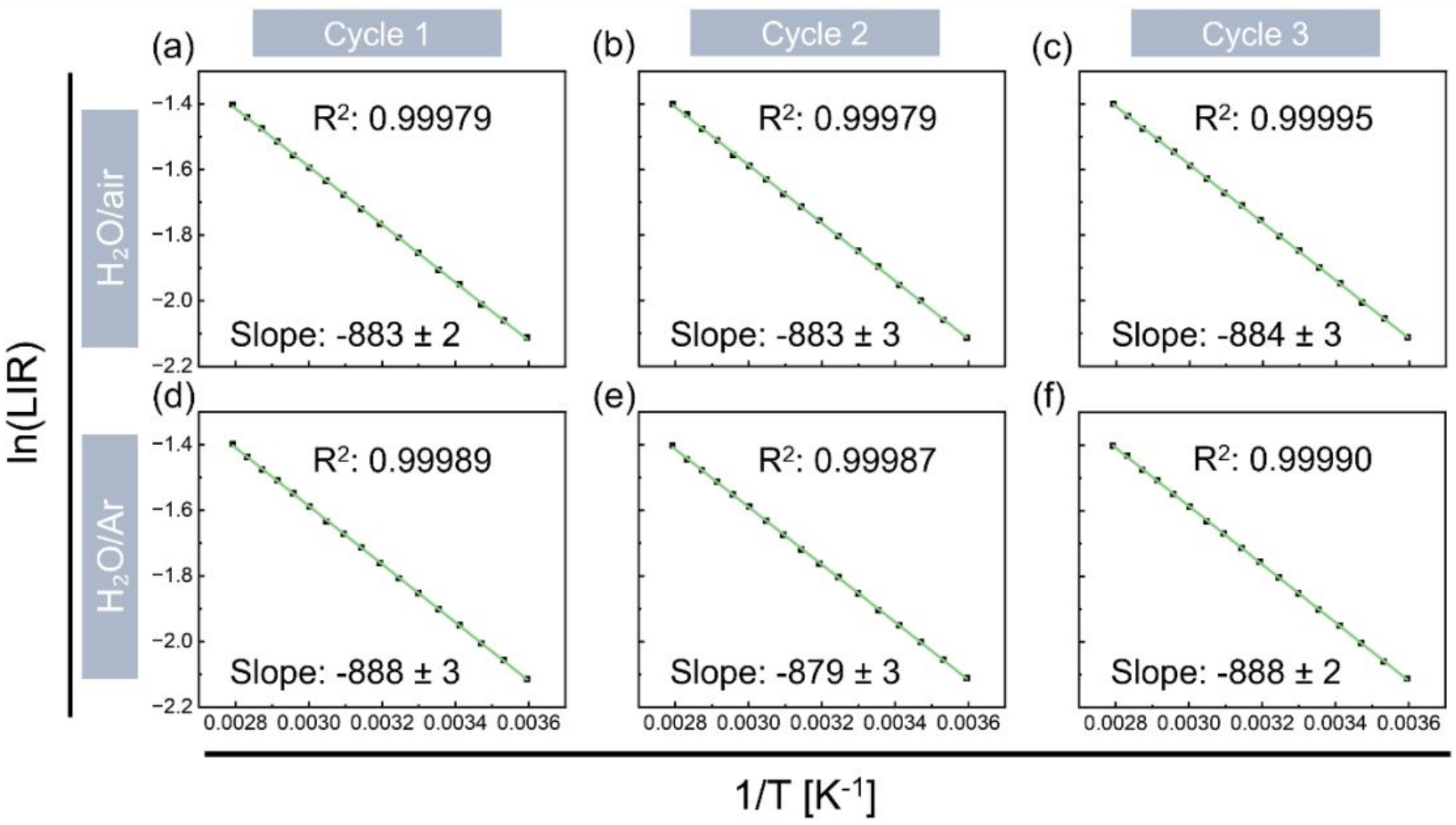

Figure S40. Boltzmann-type calibration of the temperature response of the **Crddpd**-containing samples in water under air (H_2O/air, (a)-(c)) and Ar (H_2O/Ar, (d)-(f)). Linearised Boltzmann analysis of the ratiometric signal, shown as ln(LIR) plotted versus $1/T(K^{-1})$, for three consecutive heating cycles. Solid lines represent linear fits; the coefficient of determination (R^2) and fitted slope (± fit error) are indicated in each panel, demonstrating the high reproducibility of the temperature dependence of the luminescence across cycles and atmospheres.

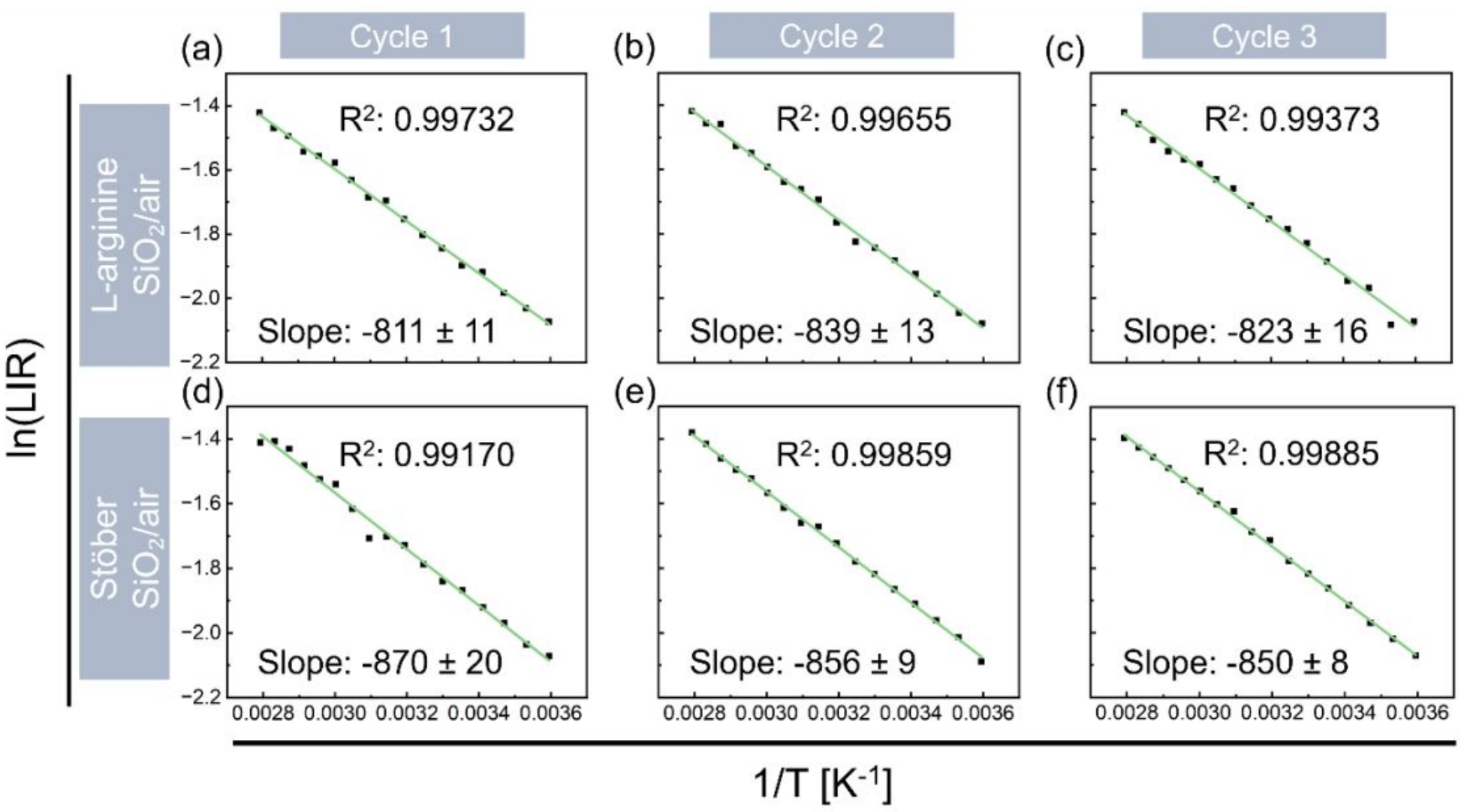

Figure S41. Boltzmann-type calibration of the temperature response of **Crddpd**-incorporated in SiO_2 NPs: **Crddpd@ARGSiO$_2$** ((a)-(c)) and **Crddpd@STOSiO$_2$** ((d)-(f)) samples dispersed in water under air. Linearised Boltzmann analysis of the ratiometric signal, shown as ln(LIR) plotted versus $1/T(K^{-1})$, for three consecutive heating cycles. Solid lines represent linear fits; the coefficient of determination (R^2) and fitted slope (± fit error) are indicated in each panel, demonstrating the high reproducibility of the temperature dependence of the luminescence across cycles and atmospheres.

Table S4. Summary of the Boltzmann slopes and the derived energy gaps for the four measurement environments for **Crddpd**-containing samples measured in three consecutive heating cycles. Boltzmann-type linearization slopes obtained from fits of ln(LIR) versus 1/T (K^{-1}) for all datasets measured in the four environments (e.g., H_2O/air, H_2O/Ar, L-arginine SiO_2/air (**Crddpd@ARGSiO$_2$**), and Stöber SiO_2/air (**Crddpd@STOSiO$_2$**). For each environment, individual slope values (from repeated cycles/replicates) are listed together with the mean slope (± SD). The corresponding energy gap ΔE was calculated from the average slope according to Eq. 8.

**Boltzmann slope**					
	Cycle 1 [K]	Cycle 2 [K]	Cycle 3 [K]	Average [K]	ΔE [cm^{-1}]
H_2O/air	-883 ± 2	-883 ± 3	-884 ± 3	-883 ± 2	614 ± 1
H_2O/Ar	-888 ± 3	-879 ± 3	-888 ± 2	-885 ± 2	615 ± 1
L-arginine SiO_2/air **(Crddpd@ARGSiO$_2$)**	-811 ± 11	-839 ± 13	-823 ± 16	824 ± 8	573 ± 6
Stöber SiO_2/air **(Crddpd@STOSiO$_2$)**	-870 ± 20	-856 ± 9	-850 ± 8	-859 ± 8	597 ± 6

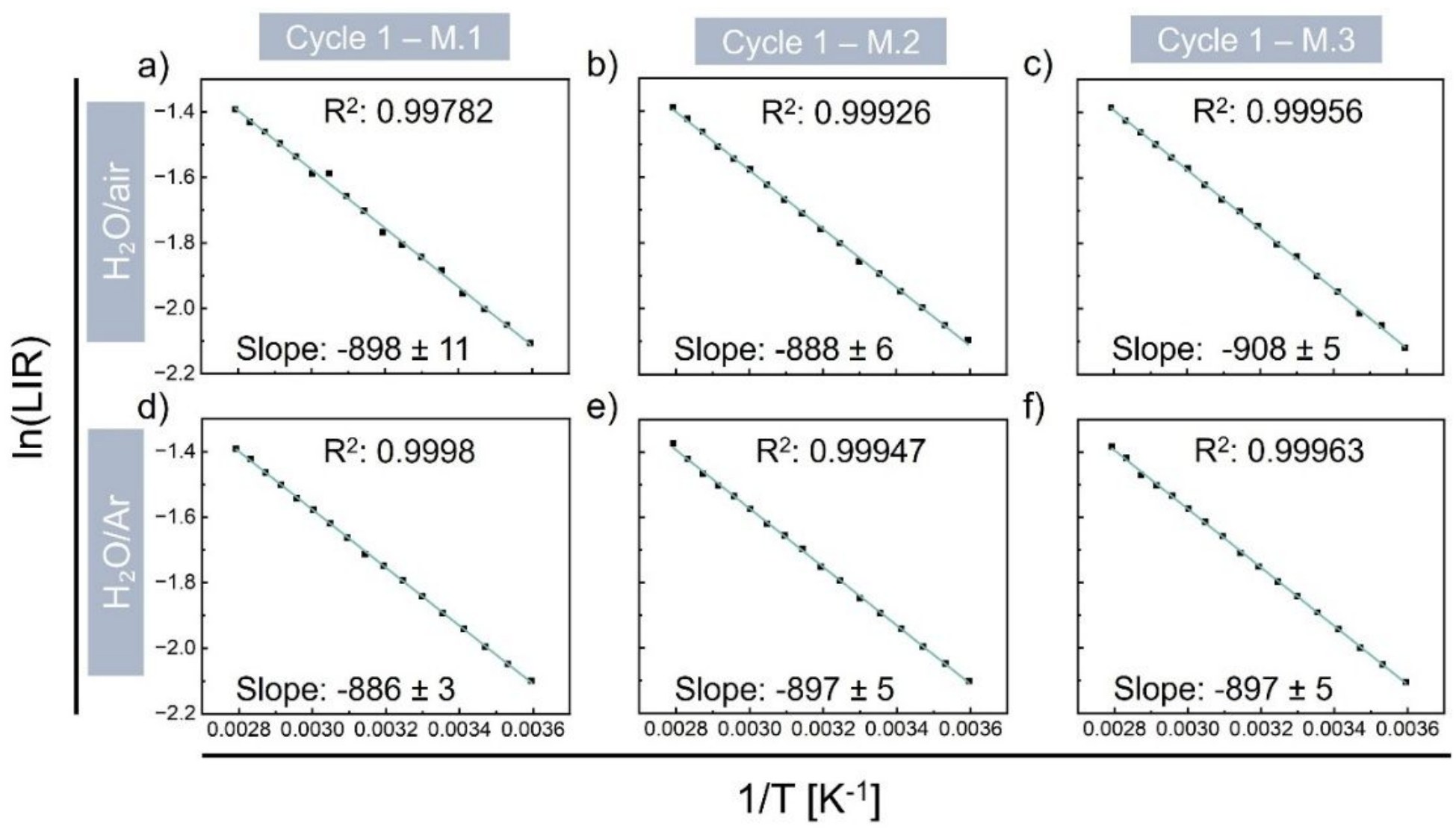

Figure S42. Boltzmann-type calibration of the temperature response of the **Crddpd**-containing samples in water under air (H_2O/air, (a)–(c)) and Ar (H_2O/Ar, (d)–(f)). Linearized Boltzmann analysis of the ratiometric signal, shown as ln(LIR) plotted versus 1/T(K^{-1}), for three independent replicate measurements of the first heating cycle. Solid lines represent linear fits; the coefficient of determination (R^2) and fitted slope (± fit error) are indicated in each panel, demonstrating highly reproducible temperature dependence across replicates and atmospheres.

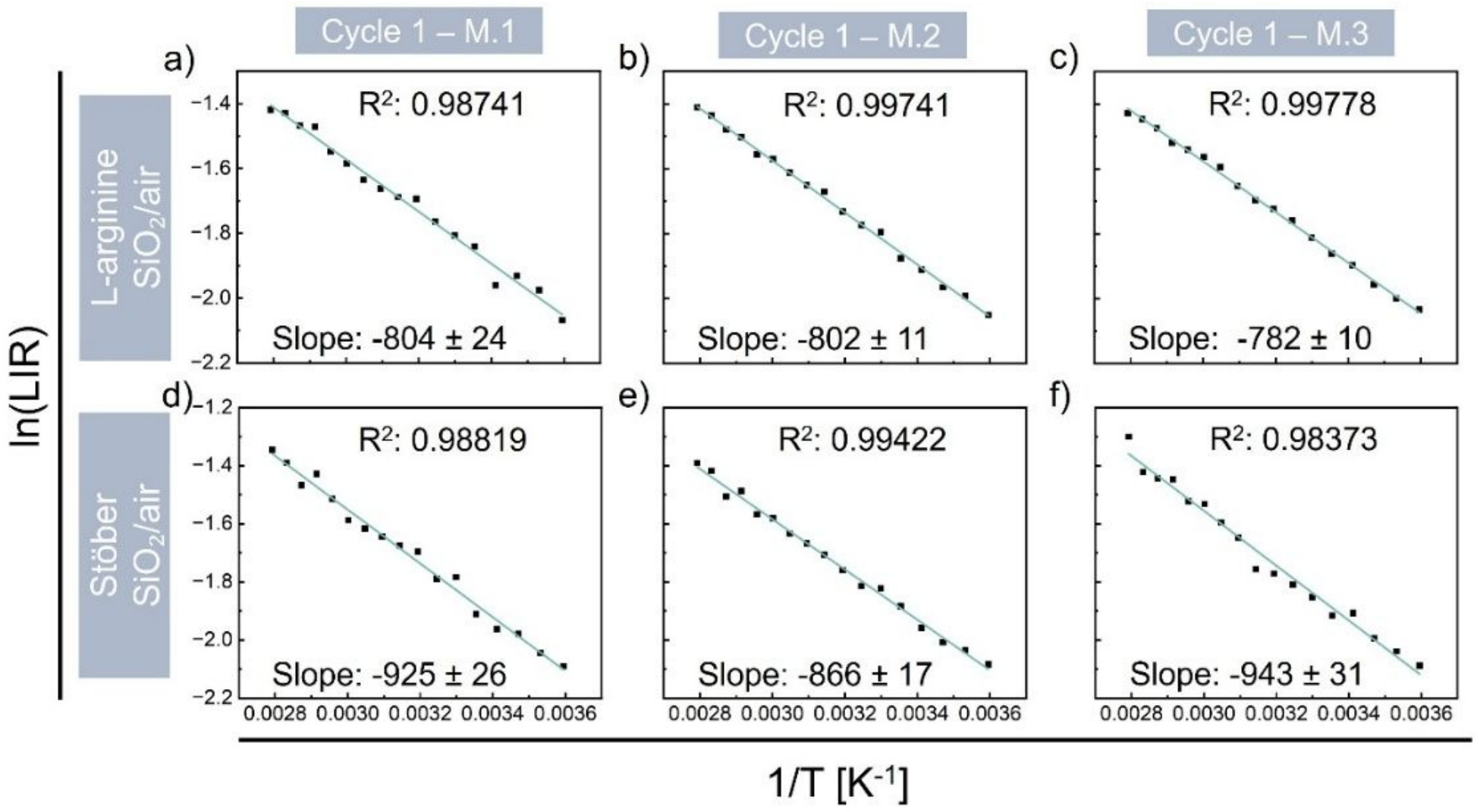

Figure S43. Boltzmann-type calibration of the temperature response of **Crddpd**-incorporated in SiO_2 NPs: **Crddpd@ARGSiO$_2$** ((a)-(c)) and **Crddpd@STOSiO$_2$**. Linearized Boltzmann analysis of the ratiometric signal, shown as ln(LIR) plotted versus 1/T(K^{-1}), for three independent replicate measurements of the first heating cycle. Solid lines represent linear fits; the coefficient of determination (R^2) and fitted slope (± fit error) are indicated in each panel, demonstrating highly reproducible temperature dependence across replicates and atmospheres.

Table S5. Summary of Boltzmann slopes and derived energy gaps for the four measurement environments for **Crddpd**-containing samples measured in three independent replicate measurements of the first heating cycle. Boltzmann-type linearization slopes obtained from fits of ln(LIR) versus 1/T1 for all datasets measured in the four environments (e.g., H_2O/air, H_2O/Ar, L-arginine SiO_2/air (**Crddpd@ARGSiO$_2$)**, and Stöber SiO_2/air (**Crddpd@STOSiO$_2$**). For each environment, individual slope values (from repeated cycles/replicates) are listed together with the mean slope (± SD). The corresponding energy gap ΔE was calculated from the average slope according to Eq. 8.

**Boltzmann slope**					
	Cycle 1– M1 [K]	Cycle 1 – M2 [K]	Cycle 1 – M3 [K]	Average [K]	ΔE [cm^{-1}]
H_2O/air	-898 ± 11	-888 ± 6	-908 ± 5	-898 ± 5	624 ± 4
H_2O/Ar	-886 ± 3	-897 ± 5	-897 ± 5	-893 ± 3	621 ± 2
L-arginine SiO_2/air **(Crddpd@ARGSiO$_2$)**	-804 ± 24	-802 ± 11	-782 ± 10	796 ± 10	553 ± 7
Stöber SiO_2/air **(Crddpd@STOSiO$_2$)**	-925 ± 26	-866 ± 17	-943 ± 31	-911 ± 15	633 ± 10

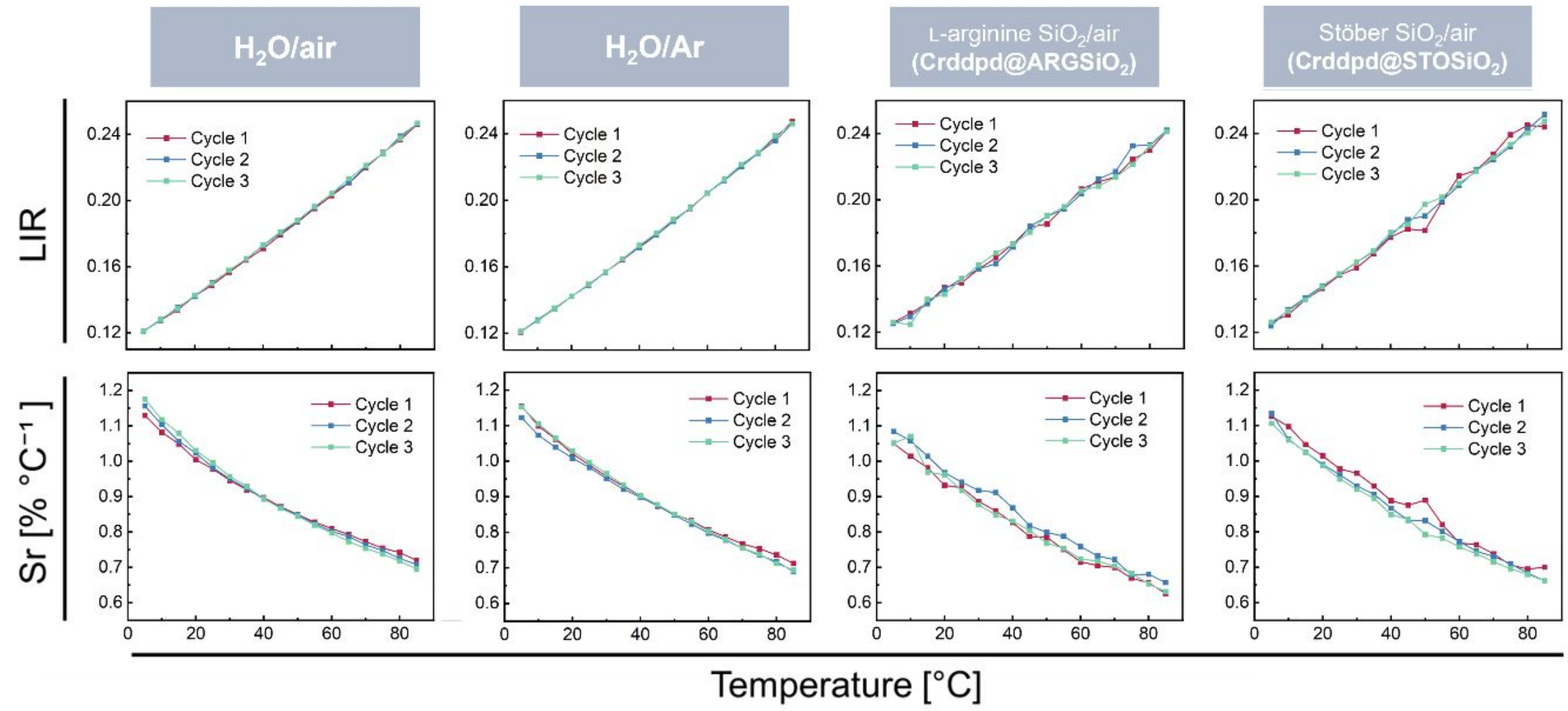

Figure S44. Temperature-dependent ratiometric response and relative sensitivity of the **Crddpd**-containing samples under different environments across repeated cycles. Top row: luminescence intensity ratio (LIR) as a function of temperature (5–85 °C). Bottom row: corresponding relative sensitivity S_r (% °C^{-1}) calculated from the fitted LIR–T dependence. Data are shown for three consecutive temperature cycles (Cycle 1–3) for a Cr^{III} in water under ambient air equilibrium (H_2O/air), Cr^{III} in argon-saturated water (H_2O/Ar), Cr^{III} incorporated in L-arginine SiO_2 NPs measured in H_2O/air (**Crddpd@ARGSiO$_2$**), and Cr^{III} incorporated in Stöber SiO_2 NPs measured in H_2O/air (**Crddpd@STOSiO$_2$**).

S5.2 Crddpd: lifetime-based temperature sensing

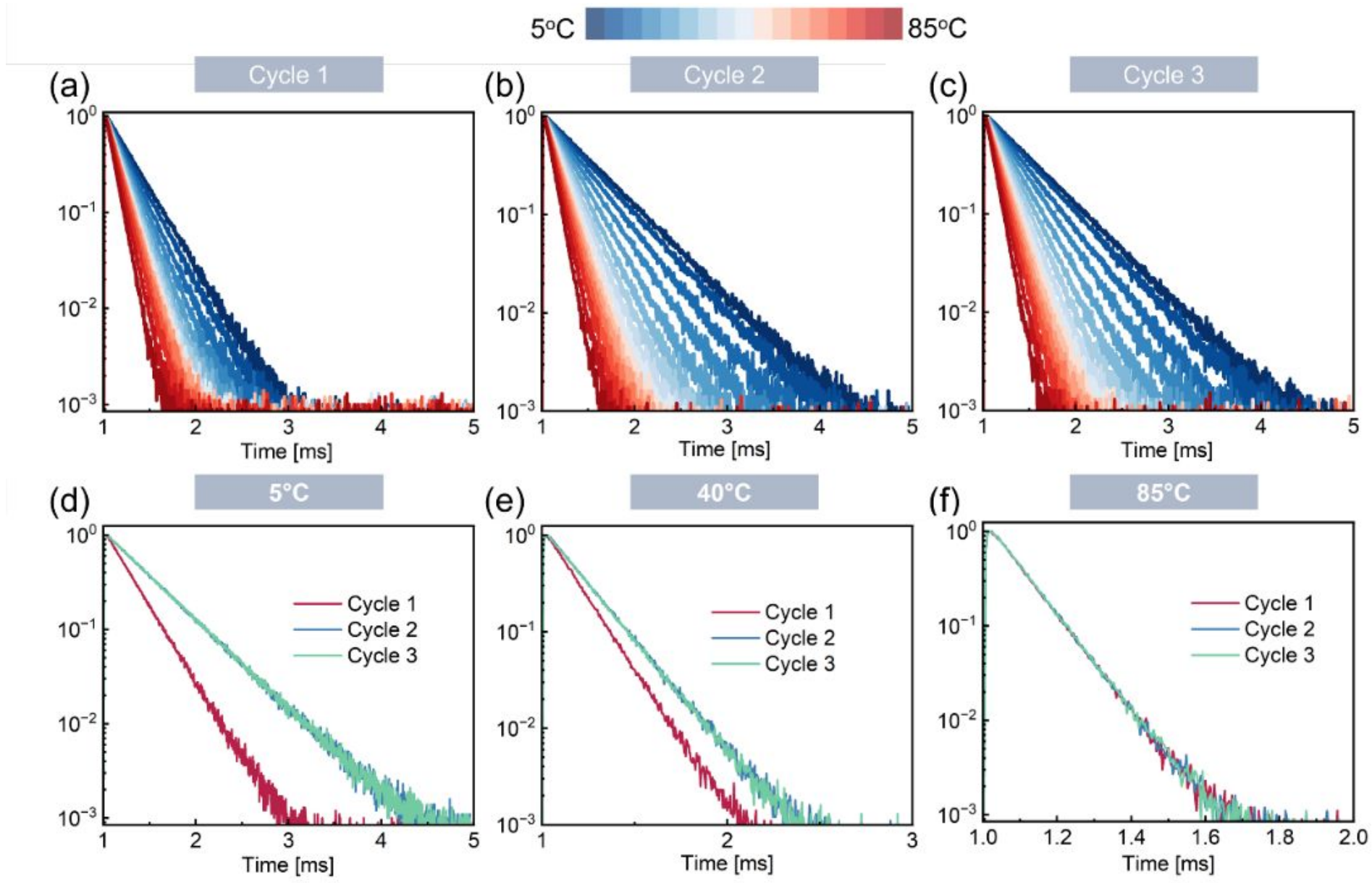

Figure S45. (a-c) Temperature-dependent time-resolved luminescence of the **Crddpd**-containing samples in water under ambient air equilibrium (H_2O/air) from 5 to 85 °C during three consecutive heating cycles (Cycle 1–3). Counts are shown on a logarithmic scale. (d–f) Overlay of the PL decay curves derived from cycles 1–3 at selected temperatures of (d) 5 °C, (e) 40 °C, and (f) 85 °C.

Table S6. Temperature-dependent mono-exponential fit parameters for the **Crddpd**-containing samples in water under ambient air equilibrium (H_2O/air). Extracted lifetime τ_1 (µs) and corresponding amplitude (Amp_1) obtained from reconvolution fitting (IRF included) of the time-resolved emission decays recorded during three consecutive heating cycles (Cycle 1–3) over 5–85 °C. The bottom row summarizes the fitted τ_1 ranges observed within each cycle.

Crddpd in H_2O/air						
	Cycle 1		Cycle 2		Cycle 3	
**T [°C]**	**τ_1 [µs]**	**Amp_1**	**τ_1 [µs]**	**Amp_1**	**τ_1 [µs]**	**Amp_1**
**5**	266	0.5074	460	0.5892	461	0.4784
**10**	240	0.5216	422	0.5721	422	0.5721
**20**	192	0.5322	309	0.5002	309	0.5033
**30**	162	0.5633	230	0.5270	230	0.5210
**40**	146	0.5416	186	0.5409	186	0.5433
**50**	136	0.5579	157	0.5399	158	0.5620
**60**	129	0.5699	138	0.5525	139	0.5781
**70**	117	0.5839	121	0.5827	120	0.5709
**80**	98	0.6034	97	0.5993	98	0.5913
**85**	85	0.6227	84	0.6297	85	0.6169
**Range**	**266 – 85 µs**		**460 – 84 µs**		**461 – 85 µs**	

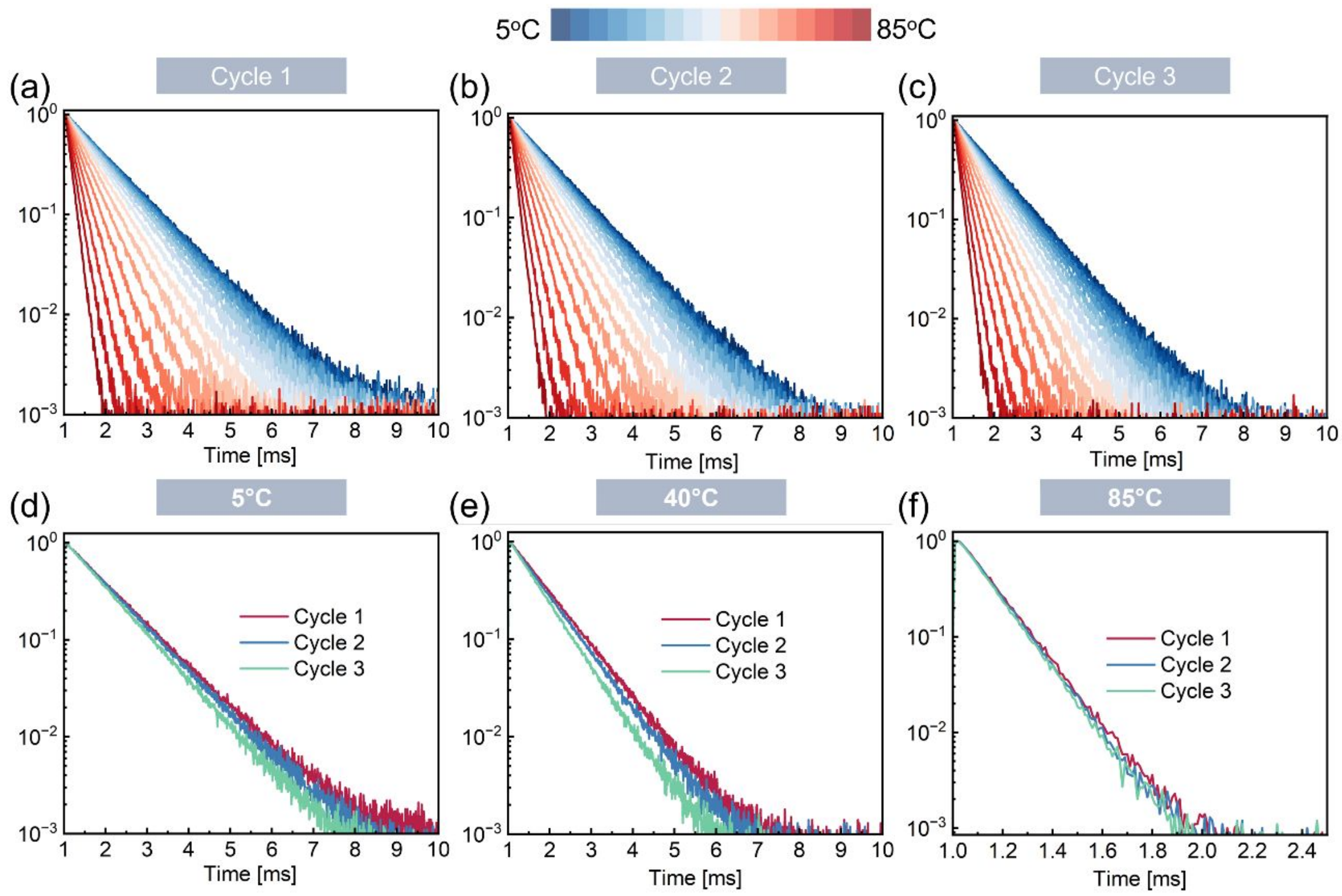

Figure S46. (a-c) Temperature-dependent time-resolved luminescence of the **Crddpd**-containing samples in water in argon-saturated conditions (H_2O/Ar) from 5 to 85 °C during three consecutive heating cycles (Cycle 1–3). Counts are shown on a logarithmic scale. (d–f) Overlay of the PL decay curves derived from cycles 1–3 at selected temperatures of (d) 5 °C, (e) 40 °C, and (f) 85 °C.

Table S7. Temperature-dependent mono-exponential fit parameters for the **Crddpd**-containing sample in water under an argon-saturated atmosphere (H_2O/Ar). Extracted lifetime τ_1 (µs) and corresponding amplitude (Amp_1) obtained from reconvolution fitting (IRF included) of the time-resolved emission decays recorded during three consecutive heating cycles (cycles 1–3) over 5–85 °C. The bottom row summarizes the fitted τ_1 ranges observed within each cycle.

Crddpd in H_2O/Ar						
	Cycle 1		Cycle 2		Cycle 3	
**T [°C]**	**τ_1 [µs]**	**Amp_1**	**τ_1 [µs]**	**Amp_1**	**τ_1 [µs]**	**Amp_1**
**5**	1008	0.5542	970	0.5621	900	0.5664
**10**	997	0.559	952	0.5617	880	0.5641
**20**	964	0.5584	911	0.5574	826	0.5651
**30**	901	0.5575	844	0.5642	749	0.5746
**40**	798	0.5660	747	0.5612	658	0.5713
**50**	646	0.5728	611	0.5755	541	0.5835
**60**	463	0.5868	442	0.5887	401	0.6011
**70**	292	0.6138	285	0.6261	265	0.6193
**80**	171	0.6576	164	0.6605	157	0.6642
**85**	127	0.6905	122	0.6996	119	0.6930
**Range**	**1008 – 127 µs**		**970 – 122 µs**		**900 – 119 µs**	

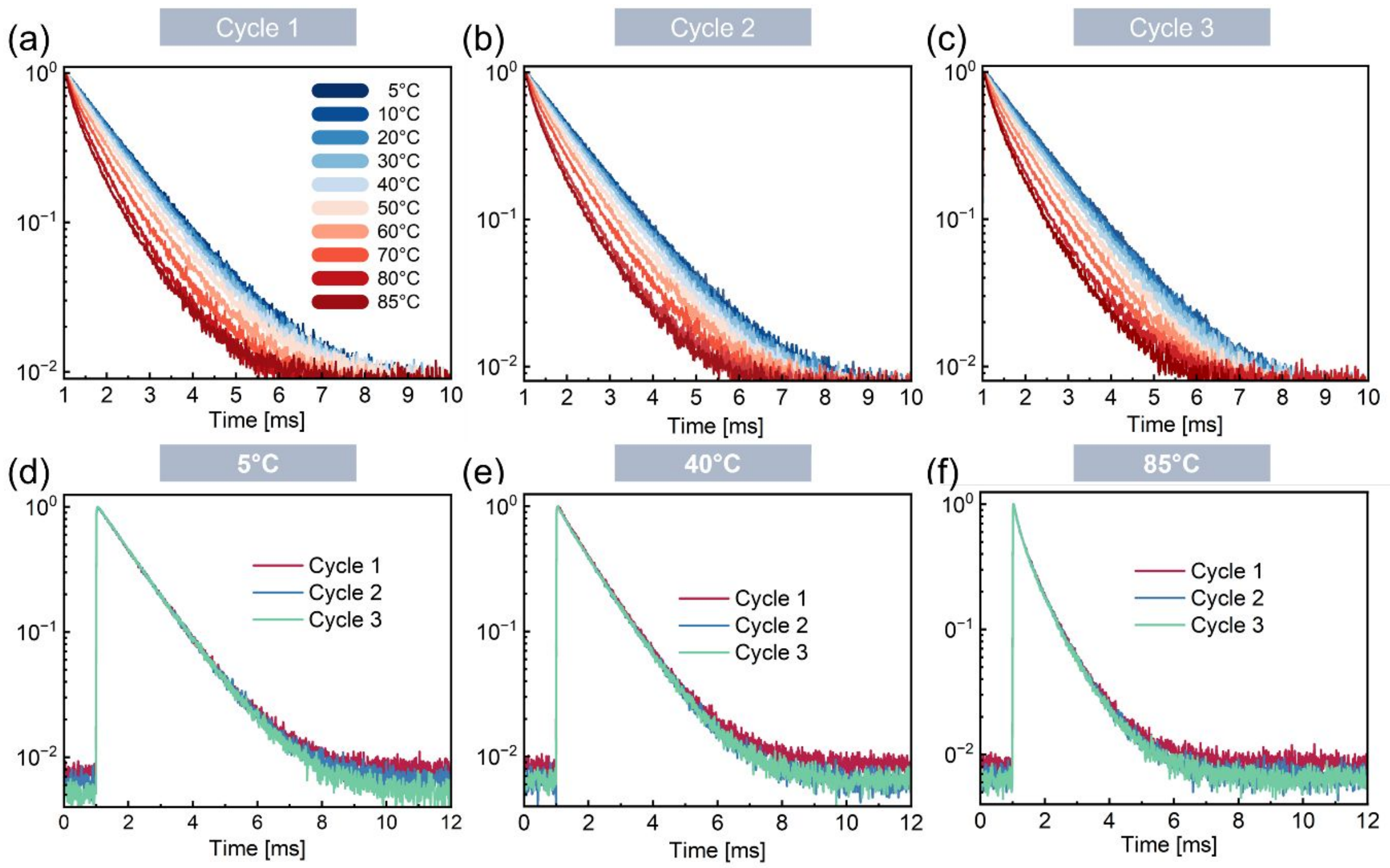

Figure S47. (a-c) Temperature-dependent time-resolved luminescence of the **Crddpd** incorporated in L-arginine SiO_2 NPs, dispersed in water under ambient air equilibrium (**Crddpd@ARGSiO$_2$**) from 5 to 85 °C during three consecutive heating cycles (Cycle 1–3). Counts are shown on a logarithmic scale. (d–f) Overlay of the PL decay curves from cycles 1–3 at selected temperatures of (d) 5 °C, (e) 40 °C, and (f) 85 °C.

Table S8. Temperature-dependent bi-exponential fit parameters for **Crddpd**-incorporated in L-arginine SiO_2 NPs dispersed in water under ambient air equilibrium (**Crddpd@ARGSiO₂**). Extracted lifetimes τ_1 (µs) and τ_2 (µs) and corresponding amplitudes (Amp_1, Amp_2) obtained from reconvolution fits (IRF included) of the PL decays recorded for three consecutive heating cycles (cycle 1–3) over 5–85 °C, as well as calculated average decay time τ_{avg}. This table contain only results obtained for the first cycle. The bottom row summarizes the fitted τ ranges observed within each cycle.

Cycle 1					
**T [°C]**	**τ_1 [µs]**	**Amp_1**	**τ_2 [µs]**	**Amp_2**	**τ_{AVG}**
**5**	1177	0.5546	-	-	1177
**10**	1166	0.5514	-	-	1166
**20**	1145	0.5582	-	-	1145
**30**	1163	0.4805	640	0.0740	1122
**40**	1135	0.4586	592	0.1030	1078
**50**	1087	0.4358	489	0.1175	1022
**60**	1031	0.4141	391	0.1482	954
**70**	987	0.3449	371	0.2134	870
**80**	810	0.3085	295	0.2668	775
**85**	877	0.2766	264	0.2889	730
**Range**	**1177 – 877 µs**		**640 – 264 µs**		**1177 – 730 µs**

Table S9. Temperature-dependent bi-exponential fit parameters for **Crddpd**-incorporated in L-arginine SiO_2 NPs dispersed in water under ambient air equilibrium (**Crddpd@ARGSiO₂**). Extracted lifetimes τ_1 (µs) and τ_2 (µs) and corresponding amplitudes (Amp_1, Amp_2) obtained from reconvolution fits (IRF included) of the PL decays recorded during three consecutive heating cycles (cycle 1–3) over 5–85 °C and calculated average decay time τ_{avg}. This table contain only results for the second cycle. The bottom row summarizes the fitted τ ranges observed within each cycle.

Cycle 2					
**T [°C]**	**τ_1 [µs]**	**Amp_1**	**τ_2 [µs]**	**Amp_2**	**τ_{AVG}**
**5**	1177	0.5545	-	-	1177
**10**	1171	0.5530	-	-	1171
**20**	1143	0.5484	-	-	1143
**30**	1161	0.4742	635	0.0827	1115
**40**	1119	0.4686	501	0.0881	1071
**50**	1100	0.4101	491	0.1453	1017
**60**	1039	0.3897	419	0.1751	944
**70**	967	0.3573	333	0.2082	861
**80**	900	0.3020	279	0.2593	770
**85**	880	0.2743	257	0.3036	728
**Range**	**1177 – 880 µs**		**635 – 257 µs**		**1177 – 728 µs**

Table S10. Temperature-dependent bi-exponential fit parameters for **Crddpd**-incorporated in L-arginine SiO_2 NPs dispersed in water under ambient air equilibrium (**Crddpd@ARGSiO$_2$**). Extracted lifetimes τ_1 (µs) and τ_2 (µs) and corresponding amplitudes (Amp_1, Amp_2) obtained from reconvolution fits (IRF included) of the PL decays recorded during three consecutive heating cycles (cycle 1–3) over 5–85 °C and calculated average decay time τ_{avg}. This table contain only results for the third cycle. The bottom row summarises the fitted τ ranges observed within each cycle.

Cycle 3					
**T [°C]**	**τ_1 [µs]**	**Amp_1**	**τ_2 [µs]**	**Amp_2**	**τ_{AVG}**
**5**	1181	0.5586	-	-	1181
**10**	1169	0.5493	-	-	1169
**20**	1144	0.5524	-	-	1144
**30**	1169	0.4636	638	0.0940	1116
**40**	1109	0.4770	489	0.0794	1067
**50**	1087	0.4232	470	0.1378	1011
**60**	1026	0.3941	392	0.1656	938
**70**	970	0.3423	329	0.2158	857
**80**	902	0.2968	276	0.2687	766
**85**	876	0.2715	256	0.3027	724
**Range**	**1181 – 876 µs**		**638 – 256 µs**		**1181 – 724 µs**

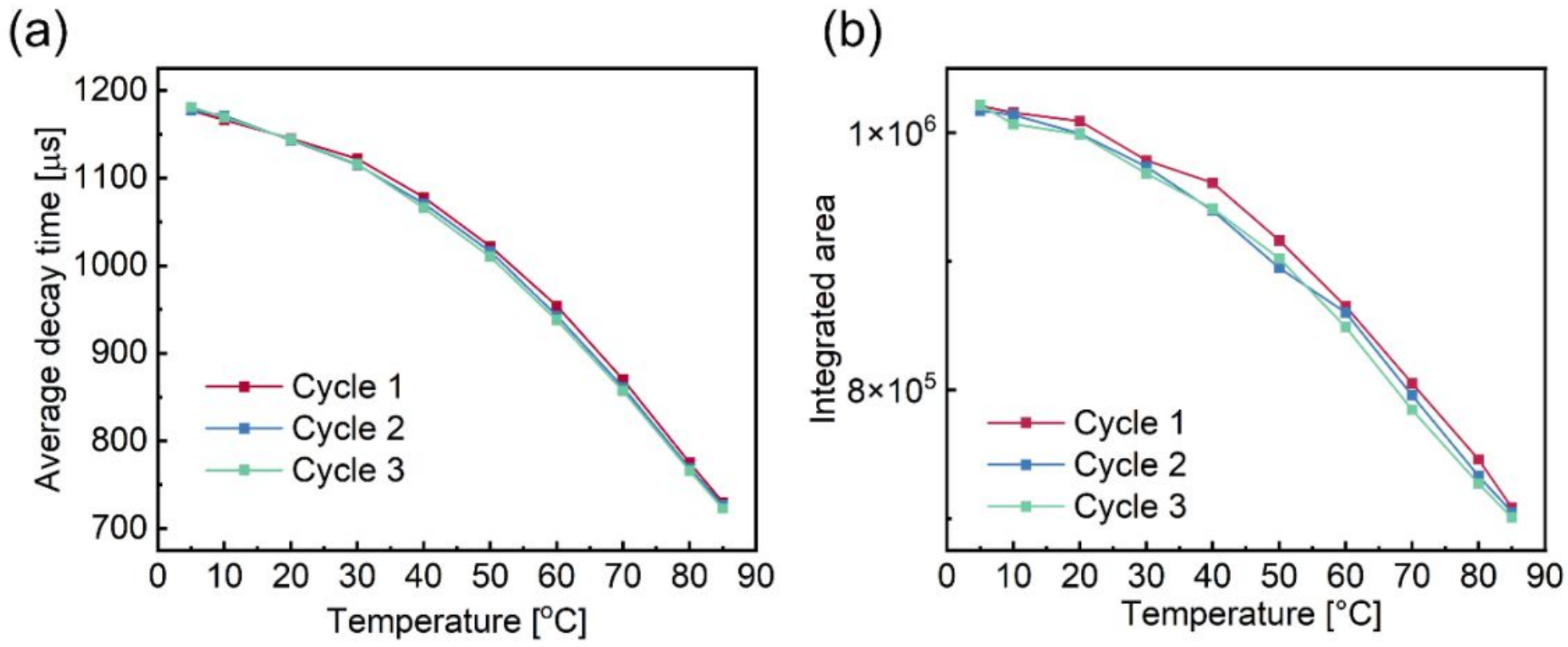

Figure S48. Lifetime-based temperature sensing with **Crddpd@ARGSiO$_2$** in air-saturated environment(a) Average decay time ⟨τ⟩ derived from a 4-exponential reconvolution fit. (b) Integral PL decays obtained by integrating the area below the normalized PL decay curves.

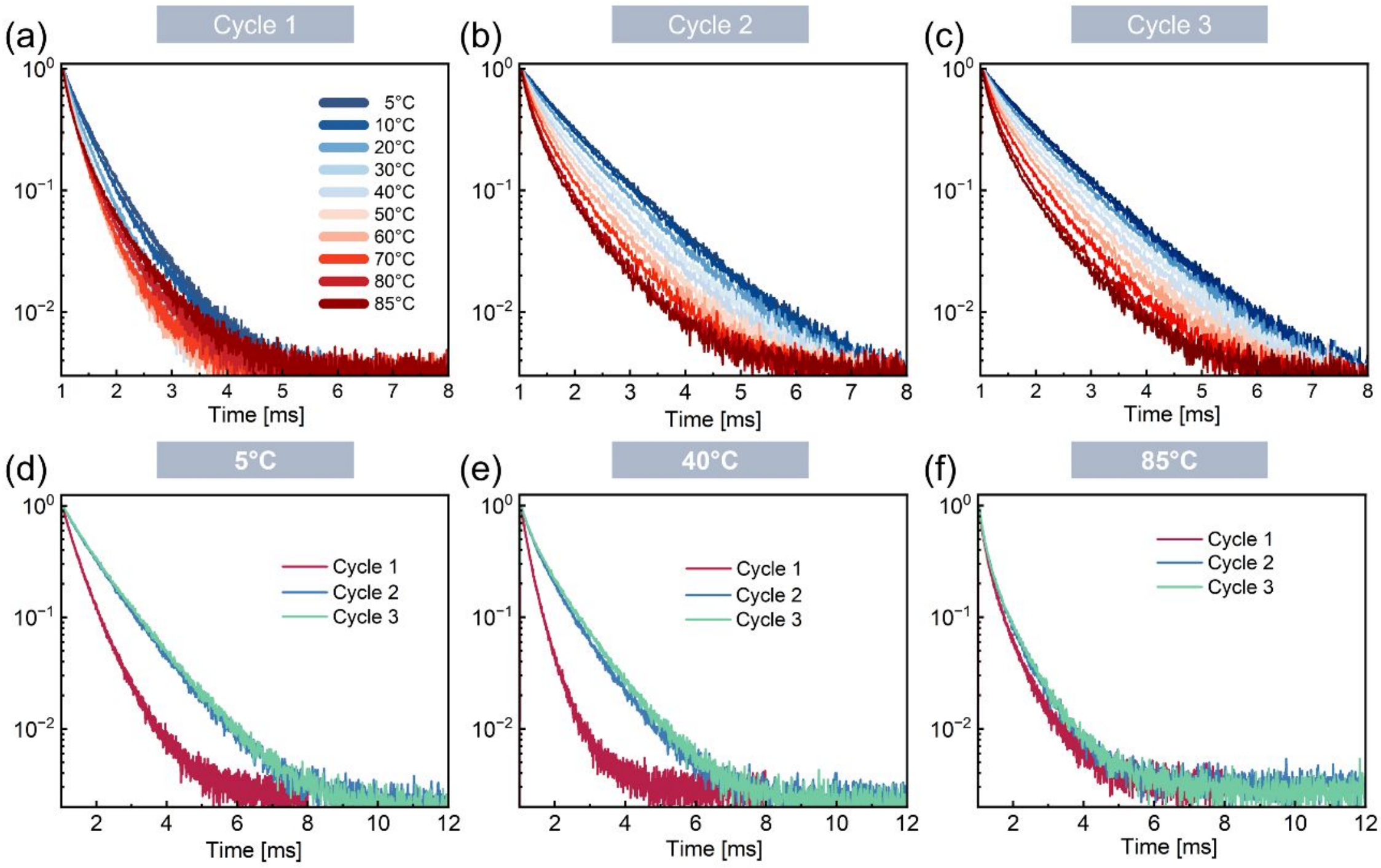

Figure S49. (a-c) Temperature-dependent time-resolved luminescence of **Crddpd** incorporated in Stöber SiO_2 NPs, dispersed in water under ambient air equilibrium (**Crddpd@STOSiO$_2$**) from 5 to 85 °C during three consecutive heating cycles (Cycle 1–3). Counts are shown on a logarithmic scale. (d–f) Overlay of decay curves from Cycles 1–3 at selected temperatures (d) 5 °C, (e) 40 °C, and (f) 85 °C.

Table S11. Temperature-dependent three-exponential fit parameters for **Crddpd** incorporated in Stöber SiO_2 NPs dispersed in water under ambient air equilibrium (**Crddpd@STOSiO$_2$**) for Cycle 1. Extracted lifetimes τ_1 (µs), τ_2 (µs) and τ_3 (µs) and the corresponding amplitudes (Amp_1, Amp_2, Amp_3) obtained from reconvolution fitting (IRF included) of the time-resolved emission decays recorded during three consecutive heating cycles (cycles 1–3) from 5–85 °Cas well as the calculated average decay time τ_{avg}. This table contain only results obtained for cycle 1. The bottom row summarizes the fitted τ ranges observed within each cycle.

**Cycle 1**					
**T [°C]**	**τ_1 [µs]**	**Amp_1**	**τ_2 [µs]**	**Amp_2**	**τ_{AVG}**
**5**	321	0.4849	702	0.1958	500
**10**	281	0.4926	640	0.1953	451
**20**	248	0.4102	599	0.1359	404
**30**	218	0.4226	553	0.1150	355
**40**	195	0.4042	497	0.1209	326
**50**	188	0.4747	473	0.1221	300
**60**	174	0.4657	448	0.1363	292
**70**	159	0.3811	450	0.1513	313
**80**	142	0.3650	485	0.1669	351
**85**	146	0.4856	556	0.1671	378
**Range**	**321 – 146 µs**		**702 – 556 µs**		**500 – 378 µs**

Table S12. Temperature-dependent three-exponential fit parameters for **Crddpd** incorporated in Stöber SiO_2 NPs dispersed in water under ambient air equilibrium (**Crddpd@STOSiO$_2$**) for cycle 2. Extracted lifetimes τ_1 (µs), τ_2 (µs) and τ_3 (µs) and the corresponding amplitudes (Amp_1, Amp_2, Amp_3) obtained from reconvolution fits (IRF included) of the PL decays recorded during three consecutive heating cycles (cycles 1–3) from 5–85 °C, as well as calculated average decay time τ_{avg}. This table contain only results obtained for cycle 2. The bottom row summarizes the fitted τ ranges observed within each cycle.

Cycle 2							
**T [°C]**	**τ_1 [µs]**	**Amp_1**	**τ_2 [µs]**	**Amp_2**	**τ_3 [µs]**	**Amp_3**	**τ_{AVG}**
**5**	1205	0.2016	762	0.2896	342	0.0750	959
**10**	1122	0.2696	659	0.2205	328	0.0941	930
**20**	1087	0.2284	718	0.1695	342	0.1842	875
**30**	1034	0.2269	573	0.1902	255	0.1865	811
**40**	990	0.2047	498	0.2119	200	0.1776	757
**50**	920	0.2047	394	0.2537	149	0.1497	695
**60**	857	0.1927	349	0.2624	131	0.1589	635
**70**	843	0.1466	357	0.2625	120	0.2207	578
**80**	851	0.0999	368	0.2482	118	0.2955	521
**85**	751	0.1292	273	0.2647	83	0.2746	492
**Range**	**1205 – 751 µs**		**762 – 273 µs**		**342 – 83 µs**		**959 – 492 µs**

Table S13. Temperature-dependent three-exponential fit parameters for **Crddpd**-incorporated in Stöber SiO_2 NPs dispersed in water under ambient air equilibrium (**Crddpd@STOSiO$_2$**) for cycle 3. Extracted lifetimes τ1 (µs), τ2 (µs) and τ3 (µs) and the corresponding amplitudes (Amp1, Amp2, Amp3) obtained from reconvolution fits (IRF included) of the PL decays recorded during three consecutive heating cycles (cycles 1–3) from 5–85 °C, as well as calculated average decay time τavg. This table contains only the results obtained for cycle 3. The bottom row summarizes the fitted τ ranges observed within each cycle.

Cycle 3							
**T [°C]**	**τ_1 [µs]**	**Amp_1**	**τ_2 [µs]**	**Amp_2**	**τ_3 [µs]**	**Amp_3**	**τ_{AVG}**
**5**	1126	0.3411	638	0.1512	392	0.0720	995
**10**	1139	0.3034	599	0.2101	251	0.0584	972
**20**	1145	0.2320	748	0.1954	303	0.1774	923
**30**	1054	0.2915	469	0.2065	188	0.1299	872
**40**	1035	0.2374	509	0.2020	182	0.1884	817
**50**	1011	0.1969	471	0.243	155	0.1800	760
**60**	958	0.1714	449	0.2336	158	0.2000	696
**70**	923	0.1385	424	0.2292	147	0.2500	628
**80**	865	0.1205	364	0.2496	115	0.3500	543
**85**	784	0.1288	295	0.2359	94	0.2985	515
**Range**	**1126 - 784 µs**		**638 – 295 µs**		**392 – 94 µs**		**995 – 515 µs**

Table S14. Reversibility of the temperature-dependent lifetime response of **Crddpd** in water under air (H_2O/air). Mono-exponential fit parameters (τ_1 µs; Amp_1) obtained from reconvolution fits (IRF included) of the PL decays recorded from 5–85 °C for three consecutive heating cycles (cycles 1–3) and for a fourth cycle measured after re-establishing the initial O_2 concentration by purging with compressed air (cycle 4). The recovery of τ_1 values in cycle 4 demonstrates the reversibility of the luminescence response after re-establishing air-equilibrated conditions.

Crddpd in H_2O/air								
	Cycle 1		Cycle 2		Cycle 3		Cycle 4 (after purging the pressed air)	
T [°C]	τ_1 [µs]	Amp_1	τ_1 [µs]	Amp_1	τ_1 [µs]	Amp_1	τ_1 [µs]	Amp_1
**5**	254	0.7	305	0.7	341	0.7	259	0.7
**10**	225	0.7	246	0.7	289	0.7	231	0.7
**20**	183	0.7	195	0.7	227	0.7	186	0.7
**30**	156	0.7	165	0.7	187	0.7	157	0.7
**40**	144	0.7	148	0.7	161	0.7	144	0.7
**50**	142	0.7	163	0.7	164	0.7	137	0.7
**60**	151	0.7	151	0.7	151	0.7	131	0.7
**70**	132	0.7	131	0.7	131	0.7	119	0.7
**80**	104	0.7	104	0.7	104	0.7	98	0.7
**85**	90	0.7	89	0.8	89	0.8	84	0.8

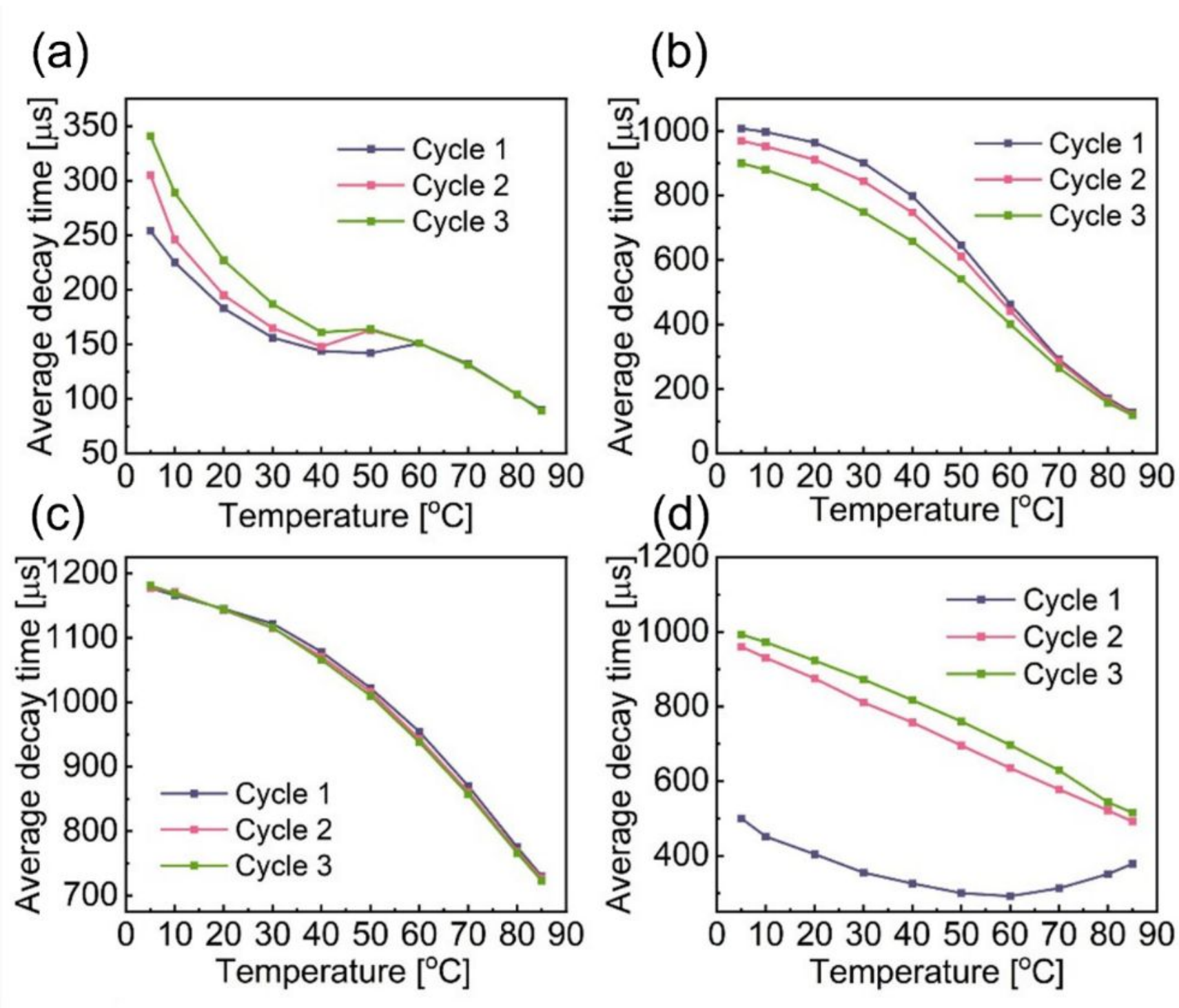

Figure S50. Comparison of the average decay time ⟨τ⟩ of **Crddpd** obtained from mono-and bi-exponential fits of the PL decay curves recorded for three consecutive heating cycles (λ_{exc} = 435 nm; 10,000 detected photons). The measurements with **Crddpd** were performed in (a) water/air, (b) water/Ar, and with (c) **Crddpd@ARGSiO₂**, and (d) **Crddpd@STOSiO₂.**

Although the acquisition times for time-resolved decays and steady-state spectra at each temperature differ, both readouts show very similar temperature-dependent trends (Figure 6(d, h, i, p) and Figure S50(a-d)). In water/air (Figure S50(a)), decay times increase slightly from cycle to cycle but converge at higher temperatures, mirroring the steady-state behaviour. In water/Ar (Figure S50(b)), the decay times slightly decrease within the three cycles, most likely due to gradual air diffusion into the cuvette, an effect amplified by the longer overall measurement time. For **Crddpd@ARGSiO$_2$** measured in air (Figure S50(c)), all three cycles agree well within the experimental uncertainty, also indicating a good stability of this sample. In contrast, **Crddpd@STOSiO$_2$** shows shorter decay times in the first cycle and an unexpected increase after heating, consistent with O_2 leaving the particles upon heating; subsequent cycles provide nearly identical results and yield an approximately linear decrease with temperature, with the third cycle revealing slightly higher, suggesting residual oxygen continues to diffuse out. A dedicated reversibility test (three cycles, re-oxygenation with pressed air, then an additional heating cycle) confirmed that the PL decay times resulting after re- introduction of O_2 closely match with the values measured in the first heating cycle (Figure S51).

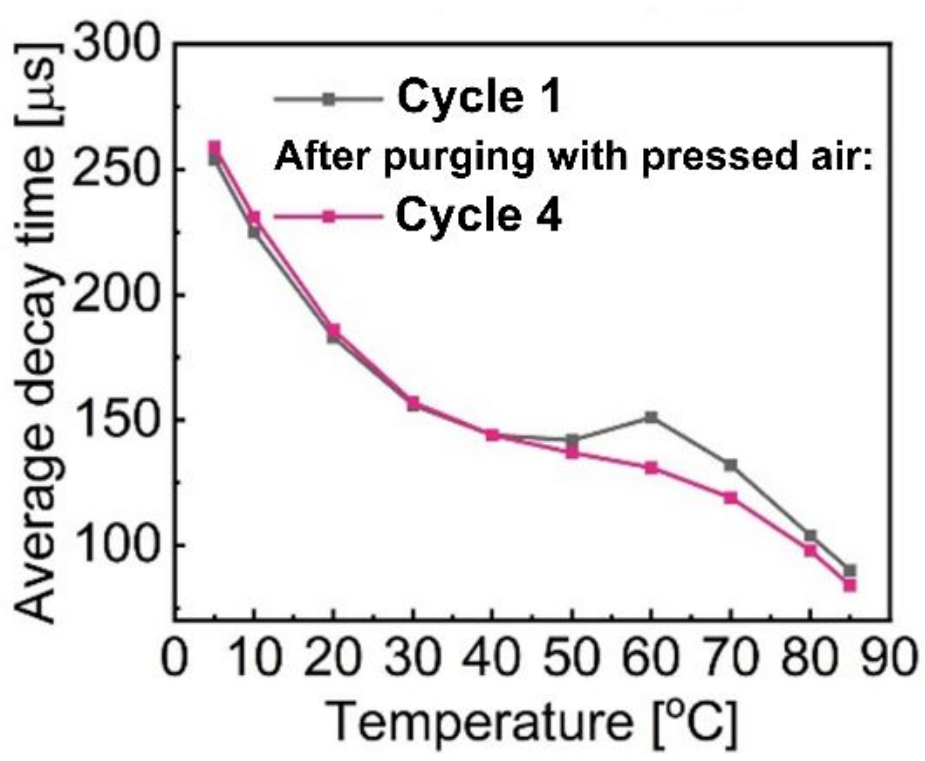

Figure S51. Reversibility test for **Crddpd** in air-saturated MilliQ water: ⟨τ⟩ recorded for the initial cycle (cycle 1) and after purging with pressed air after two cycles (cycle 4).

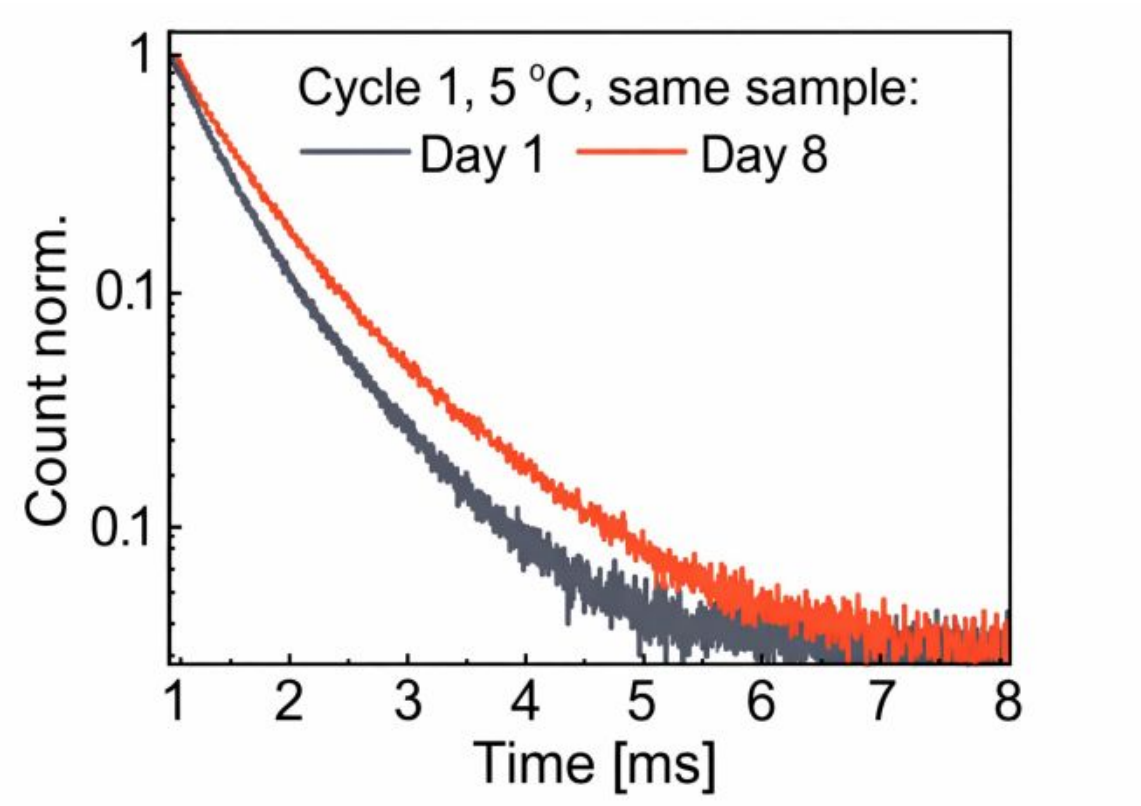

Figure S52. Aging studies performed with aging studies of **Crddpd@STOSiO$_2$** stored in air-saturated MilliQ water (pH 6.9) and monitored by time-resolved PL measurements.

Aging studies of **Crddpd@STOSiO$_2$** stored in air-saturated MilliQ water (pH 6.9) and monitored by time-resolved PL were performed. They revealed a measurable increase in the average lifetime under atmospheric conditions already within one week (Figure S52). This observation is in agreement with our previous findings and indicates that the local quenching environment evolves over time.

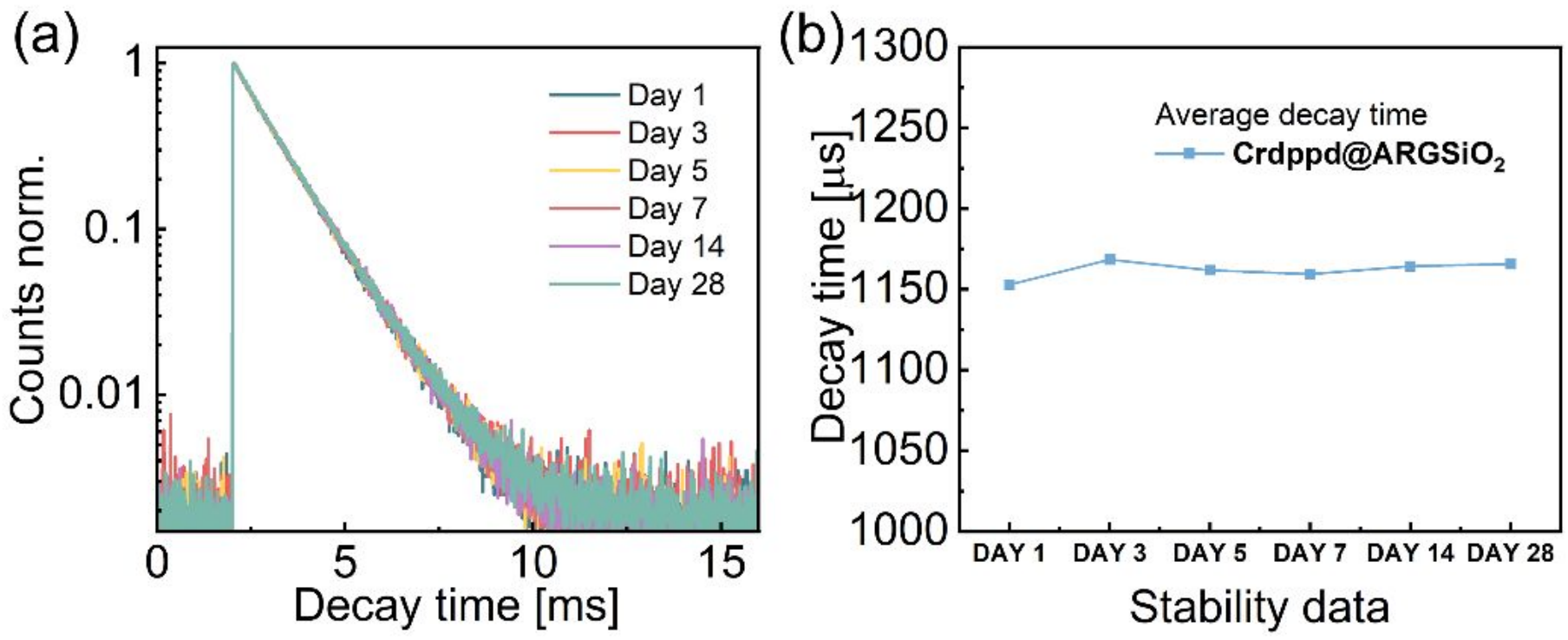

Figure S53. Ageing study of **Crddpd@ARGSiO$_2$** nanoparticles over 28 days. (a) Normalized PL decay curves recorded after 1, 3, 5, 7, 14, and 28 days of storage. (b) Intensity-weighted average decay times obtained from the corresponding decay fits.

The temporal stability of **Crddpd@ARGSiO$_2$** was assessed by monitoring the PL decay behavior over 28 days (Figure S53). The normalized decay curves remain nearly unchanged, and the intensity-weighted average decay time stays essentially constant at ca. 1.15–1.17 ms. This indicates that **Crddpd@ARGSiO$_2$** retains stable oxygen-shielded luminescence behavior over at least 28 days under the applied storage and measurement conditions.

S5.3. Normalized integrated-emission-area analysis of Crddpd and Ruphenpy during temperature cycling

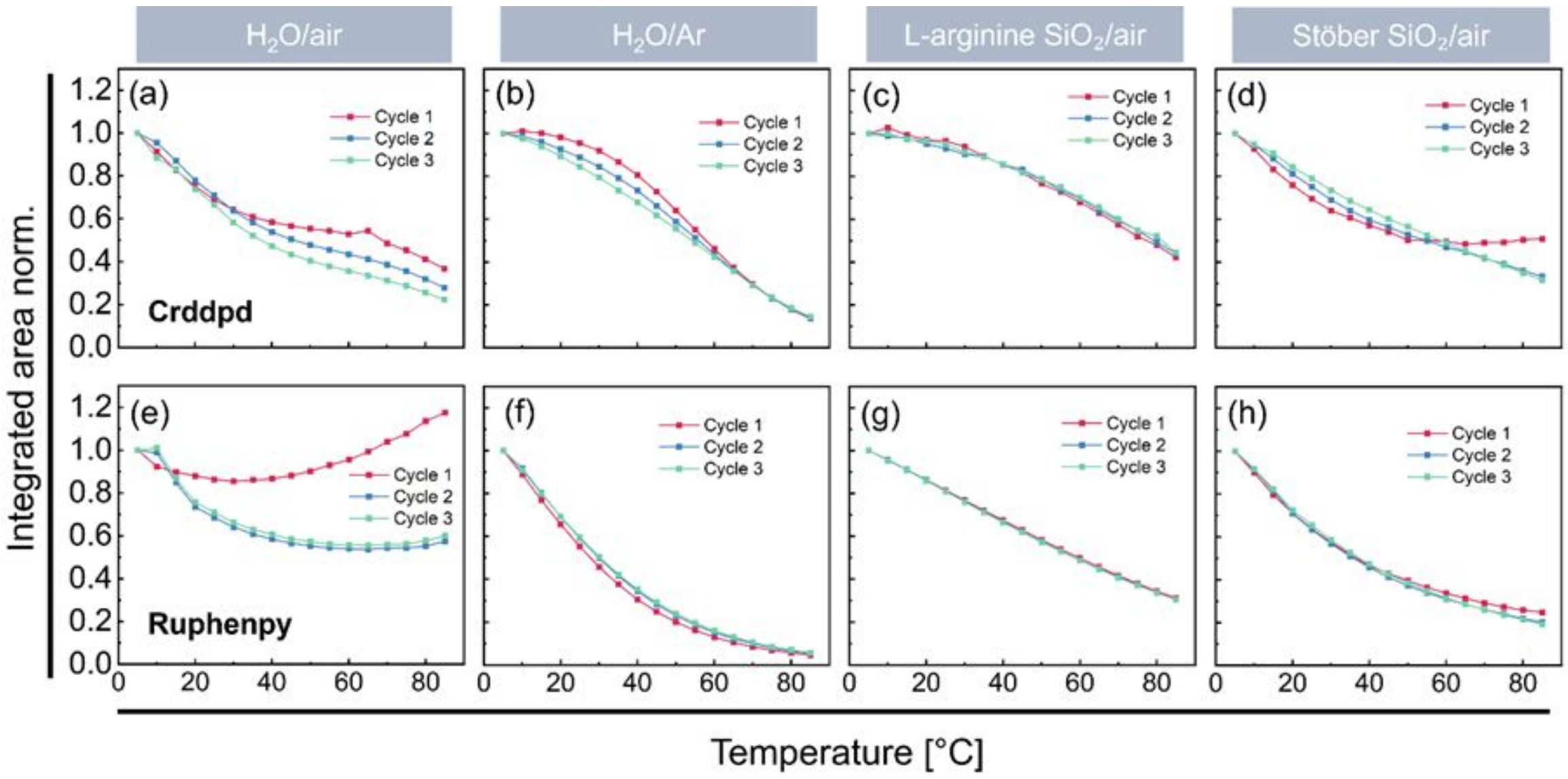

Figure S54. Normalized temperature-dependent integrated emission areas of **Crddpd** (a–d) and **Ruphenpy** (e–h) in air- and Ar-saturated aqueous solution and encapsulated in L-arginine- and Stöber-type SiO_2 NPs dispersed in air-saturated water. For each sample and each heating cycle, the integrated emission area was normalized to the corresponding value at 5 °C.

$$Relative\ decrease = \frac{A_{5°C} - A_{85°C}}{A_{5°C}} \times 100\ \% \qquad (11)$$

Table S15. Relative decrease of the normalized integrated emission area between 5 and 85 °C of **Crddpd** and **Ruphenpy** in air- and Ar-saturated aqueous solution and encapsulated in L-arginine- and Stöber-type SiO_2 NPs dispersed in air-saturated water. Values were extracted from the normalized temperature-dependent integrated-emission-area plots shown in Figure S53, and the relative decrease was calculated using Eq.11.

	Cycle 1	Cycle 2	Cycle 3
**Crddpd**			
H_2O/air	64 %	73 %	78 %
H_2O/Ar	87 %	86 %	86 %
L-Arginine SiO_2/air	56 %	56 %	56 %
Stöber SiO_2/air	49 %	67 %	69 %
**Ruphenpy**			
H_2O/air	-17 %	43 %	40 %
H_2O/Ar	96 %	95 %	94 %
L-Arginine SiO_2/air	69 %	70 %	70 %
Stöber SiO_2/air	75 %	80 %	81 %

The normalized plots and the calculated values provide additional insight into the intensity-based temperature response. Looking at the calculated data, the most unstable response for both luminophores occurs in air-saturated water, where the first cycle deviates significantly from the subsequent ones (**Crddpd** drifting from 64 % to 78 %). Most notably, the dyad in

this environment shows an unexpected initial signal increase (-17 %) before stabilizing around 40–43 %. Conversely, the L-Arginine SiO_2 provided the most stable slope without initial drift, maintaining a 56 % relative decrease for Crddpd and ~70 % for the dyad across all three cycles. In Stöber SiO_2, both luminophores showed a lower initial response in the first cycle (49 % for **Crddpd**; 75 % for **Ruphenpy**) before stabilizing at higher quenching rates (67–69 % and 80–81 %, respectively) in cycles 2 and 3. Finally, the most pronounced thermal quenching was observed for complexes in water under argon, reaching up to 87 % for Crddpd and 96 % for Ruphenpy; while this high sensitivity sounds promising for potential thermometry applications, practical usage of these thermometers in such strictly controlled environments would be complicated.

S5.4. Ruphenpy and Ruphen: luminescence intensity-based temperature sensing

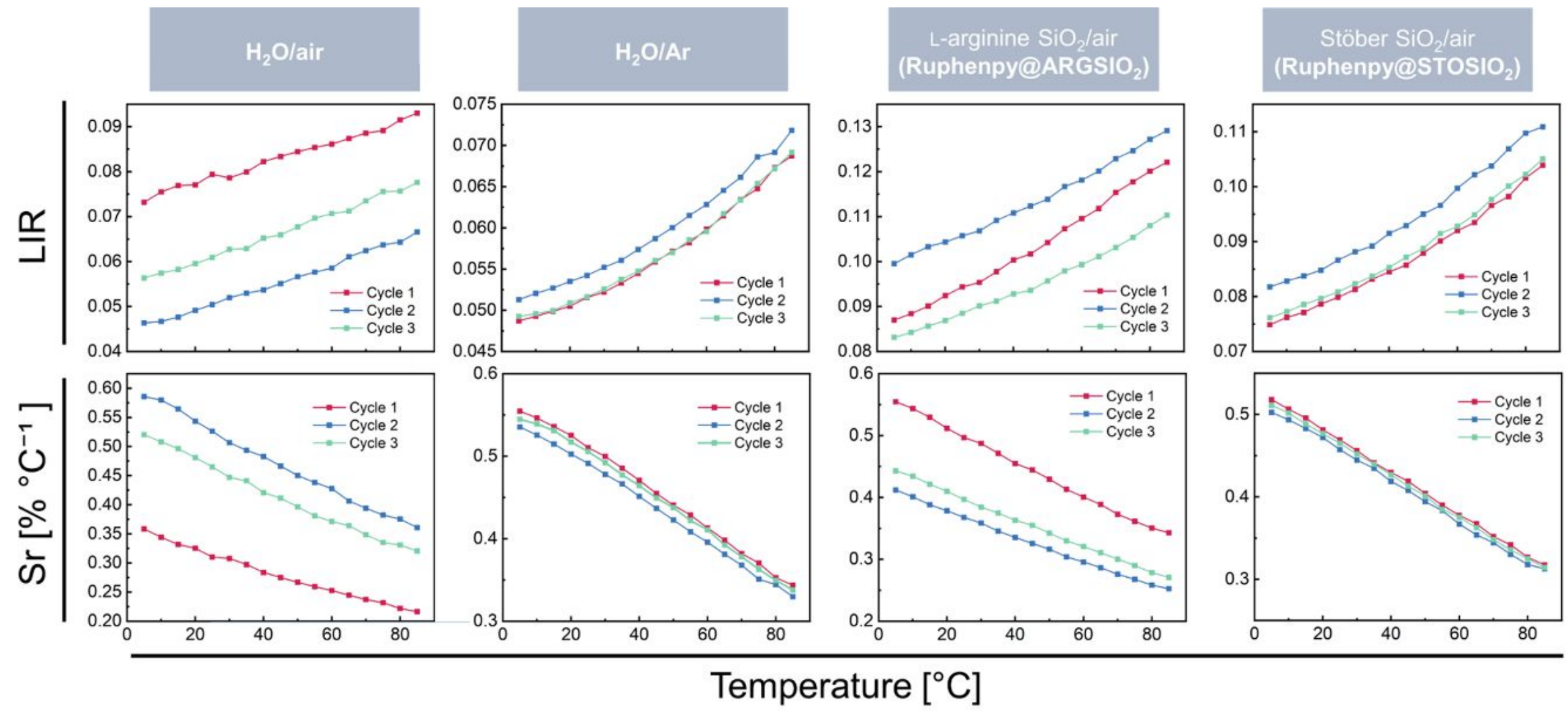

Figure S55. Temperature-dependent pseudo-ratiometric response and relative sensitivity of **Ruphenpy** under different environments across repeated heating cycles. **Top row**: luminescence intensity ratio (LIR) as a function of temperature (5–85 °C). **Bottom row**: corresponding relative sensitivity S_r (% °C^{-1}) calculated from the fitted LIR/T-dependence. Data are shown for three consecutive temperature cycles (cycles 1–3) for **Ruphenpy** in water under ambient air equilibrium (H_2O/air), **Ruphenpy** in argon-saturated water (H_2O/Ar), **Ruphenpy** incorporated in L-arginine SiO_2 NPs measured in H_2O/air (**Ruphenpy@ARGSiO$_2$**), and **Ruphenpy** incorporated in Stöber silica nanoparticles measured in H_2O/air (**Ruphenpy@STOSiO$_2$**).

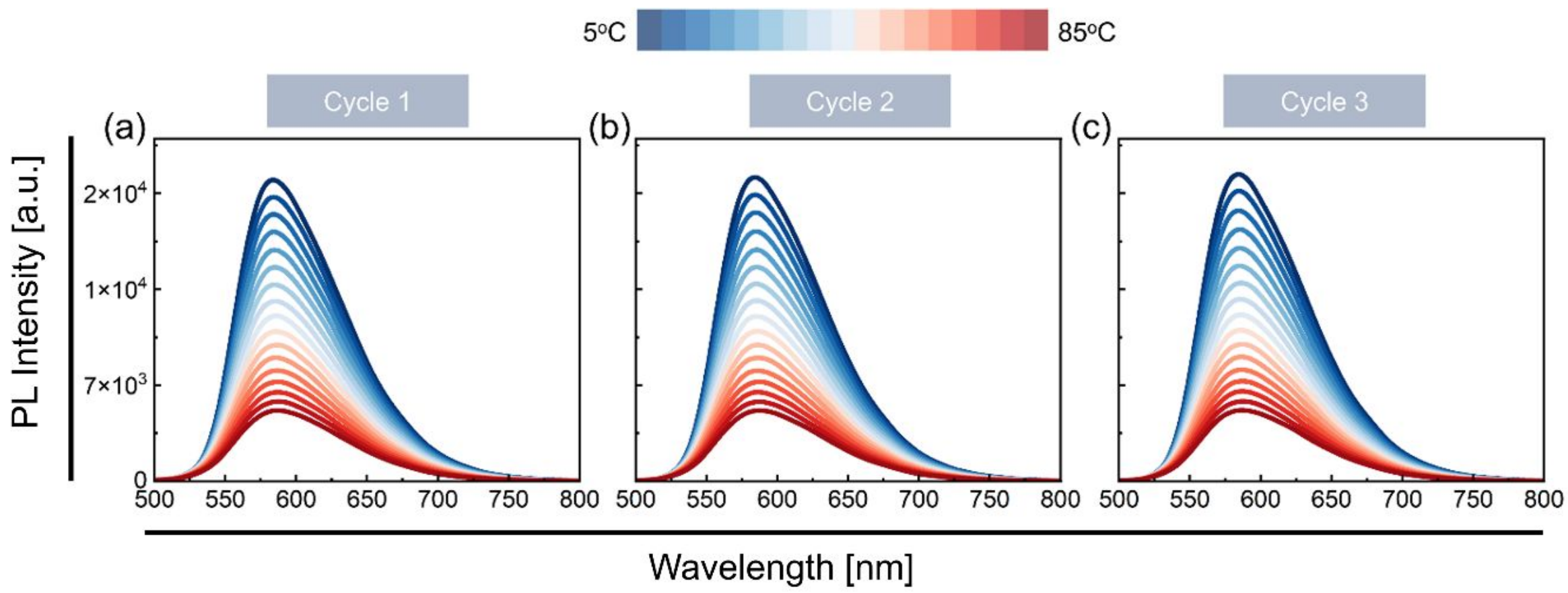

Figure S56. Temperature-dependent PL spectra of the **Ruphen** complex incorporated in L-arginine SiO_2 NPs in H_2O/air (**Ruphen@ARGSiO$_2$**) recorded over three consecutive heating–cooling cycles.

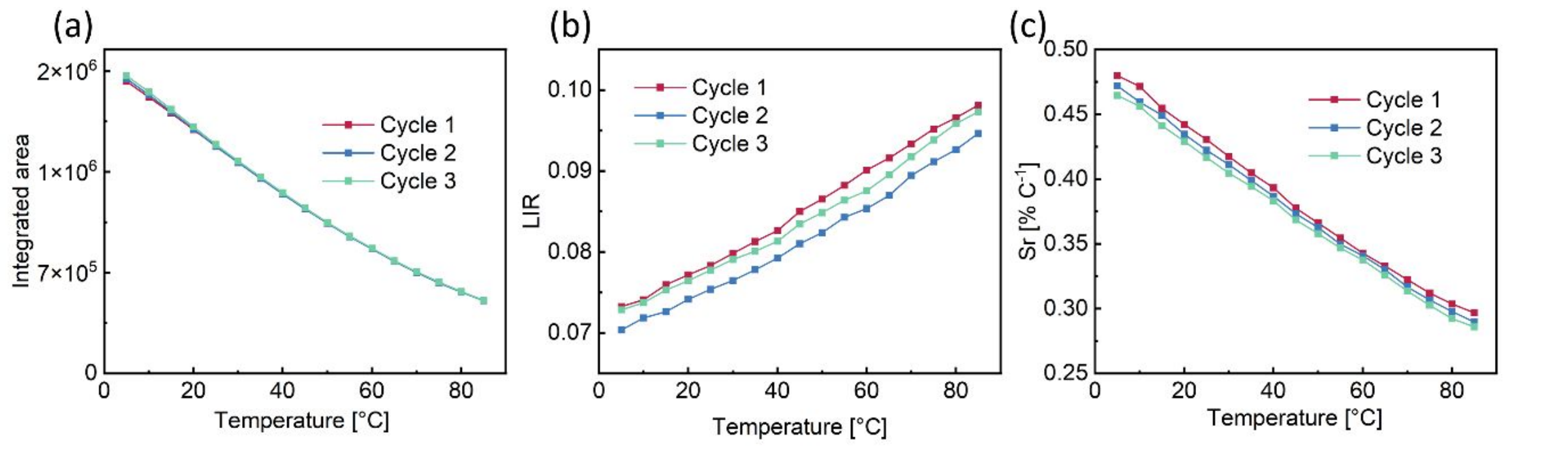

Figure S57. (a) Integrated emission area (500–800 nm) of **Ruphen@ARGSiO$_2$** as a function of temperature (5–85 °C). (b) Corresponding luminescence intensity ratio (LIR) derived from the pseudo-ratiometric analysis. (c) Relative temperature sensitivity S_r (% °C^{-1}) calculated from the fitted LIR–temperature dependence. Data are shown for three consecutive heating cycles (Cycle 1–3).

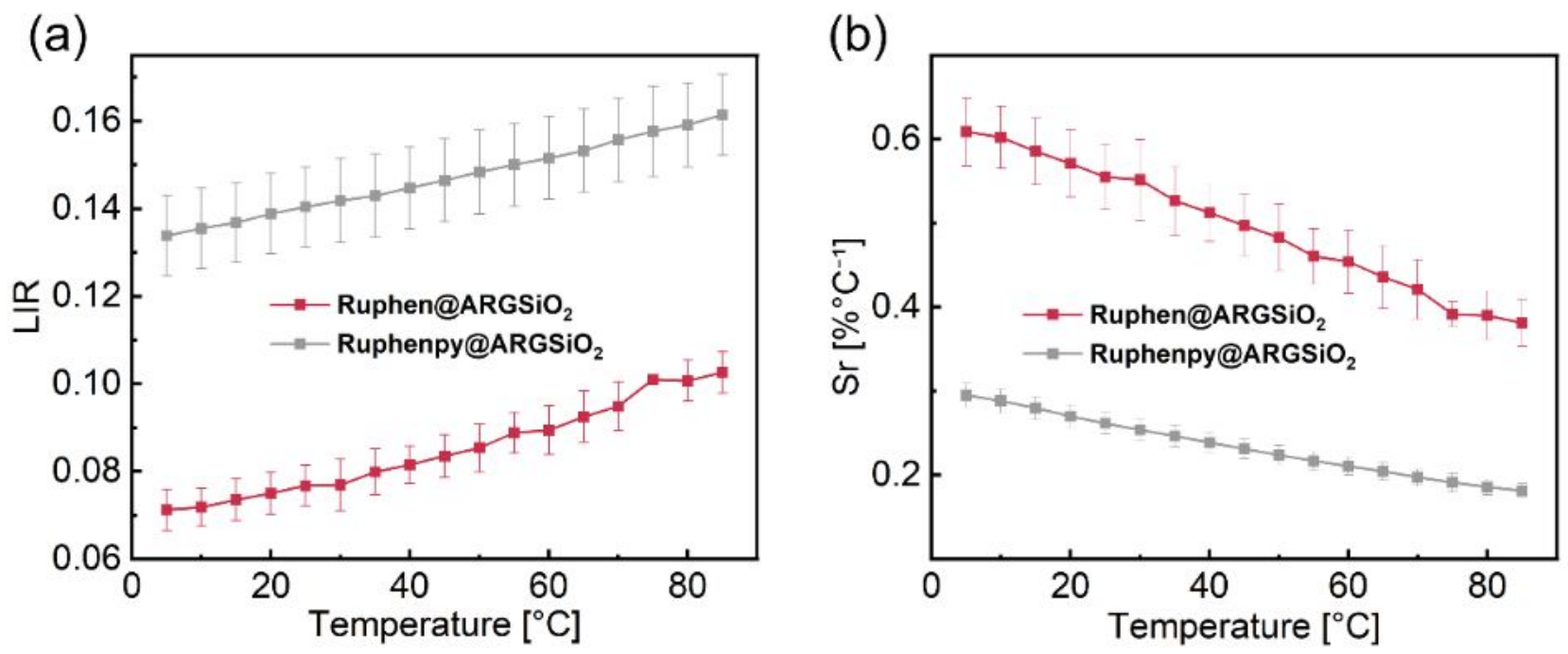

Figure S58. Comparison of the temperature-dependent pseudo-ratiometric response of **Ruphen**- and **Ruphenpy**-loaded L-arginine silica nanoparticles. (a) Luminescence intensity ratio (LIR) as a function of temperature (5–85 °C) for **Ruphen@ARGSiO$_2$** and **Ruphenpy@ARGSiO$_2$** derived from the triplicated first cycle only. (b) Corresponding relative sensitivity S_r (% °C^{-1}) calculated from the fitted LIR–temperature dependence. The error bars represent the standard deviation from triplicate measurements.

A comparison of the temperature dependence of S_r of **Ruphen@ARGSiO$_2$** and **Ruphenpy@ARGSiO$_2$** is shown in Figure S56. For both luminophores, S_r decreases steadily with increasing temperature, indicating a systematic change in the luminescence response across the measured range. **Ruphen** encapsulated in L-arginine SiO_2 NPs (**Ruphen@ARGSiO$_2$**) consistently exhibits higher S_r values (5 °C: 0.60% °C^{-1}, 85 °C: 0.38% °C^{-1}) than **Ruphenpy@ARGSiO$_2$** (5 °C: 0.29% °C^{-1}, 85 °C: 0.18% °C^{-1}). Overall, both datasets follow the same qualitative trend but clearly differ in S_r size.

S5.4 Ruphenpy: lifetime-based temperature sensing

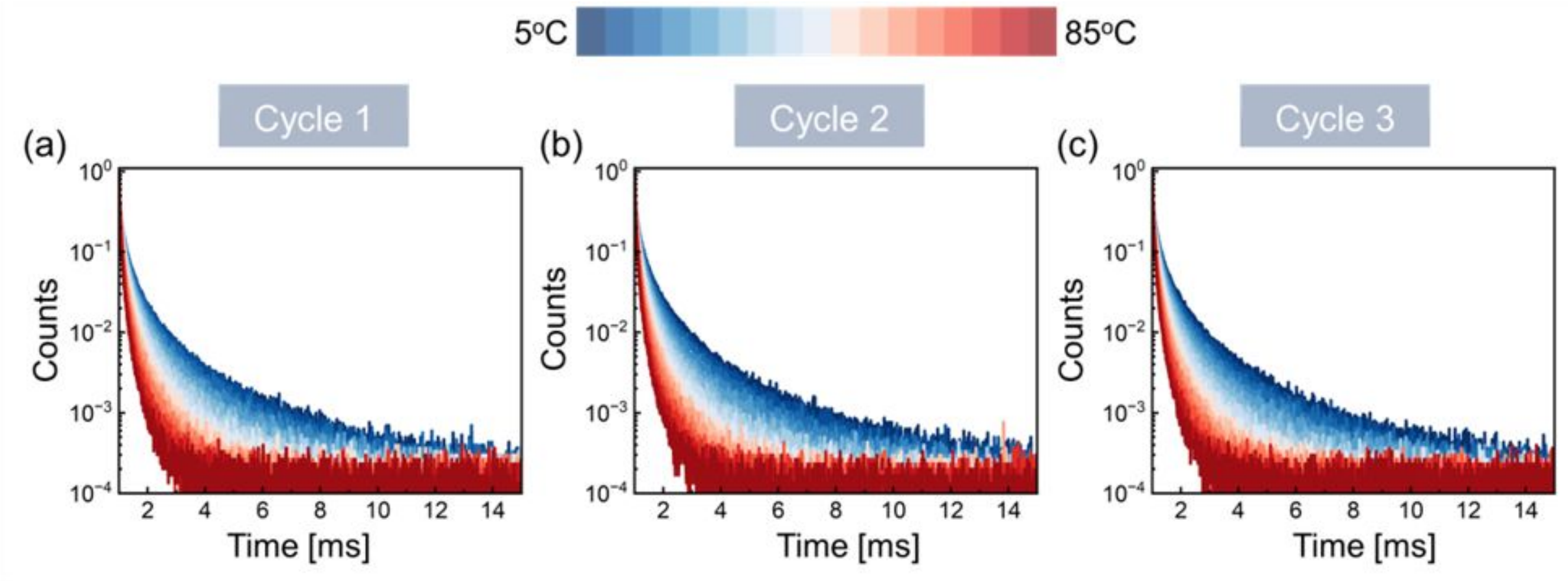

Figure S59. Temperature-dependent time-resolved PL decay curves of **Ruphenpy@ARGSiO$_2$** recorded at temperatures between 5 and 85 °C during three consecutive heating cycles: (a) cycle 1, (b) cycle 2, and (c) cycle 3. Counts are shown on a logarithmic scale.

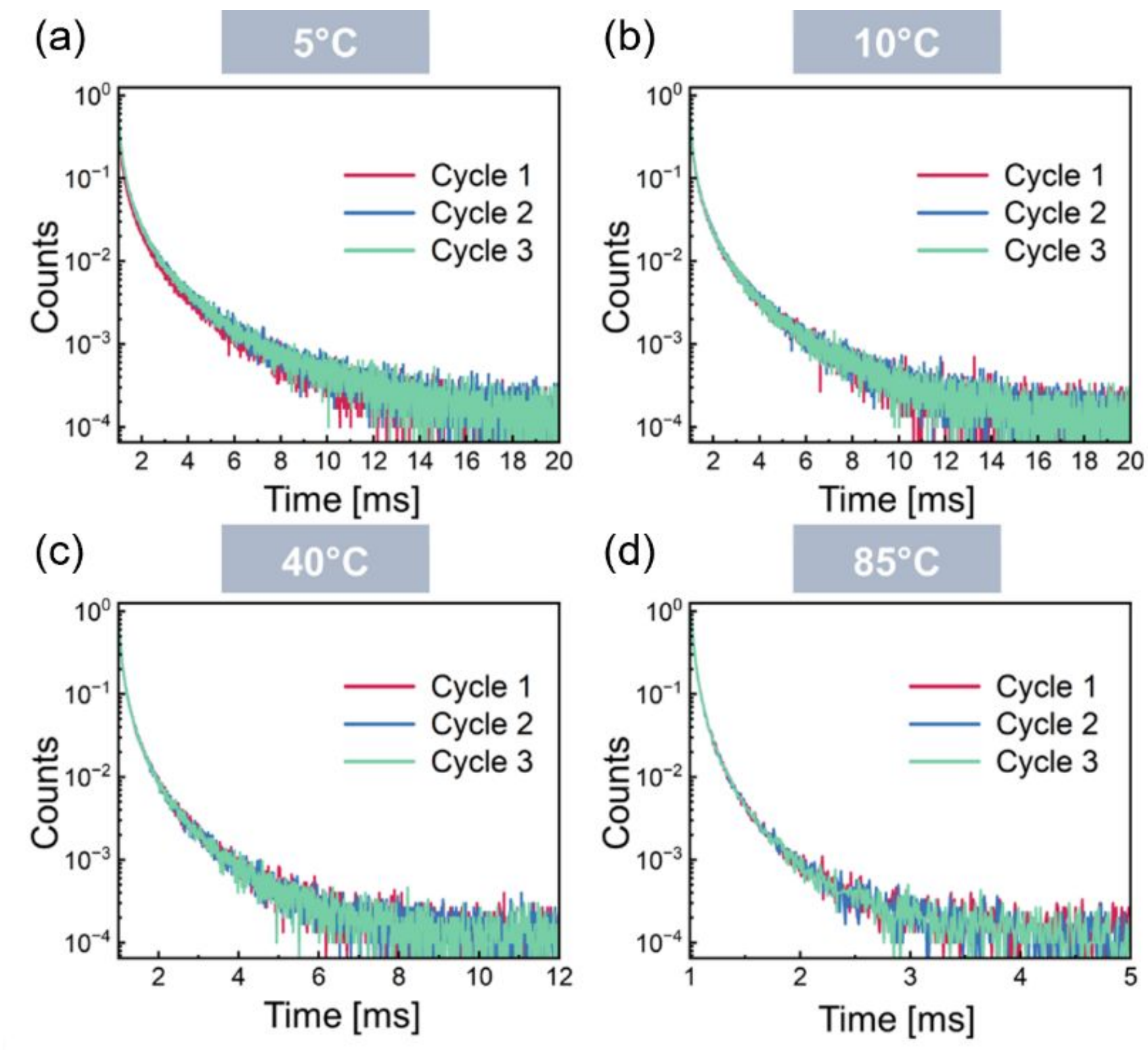

Figure S60. Reproducibility of the luminescence decay curves of **Ruphenpy@ARGSiO$_2$** at selected temperatures during three consecutive heating cycles (Cycle 1–3) at (a) 5 °C, (b) 10 °C, (c) 40 °C, and (d) 85 °C. The close overlap of the decay curves demonstrates good cycle-to-cycle reproducibility at each temperature.

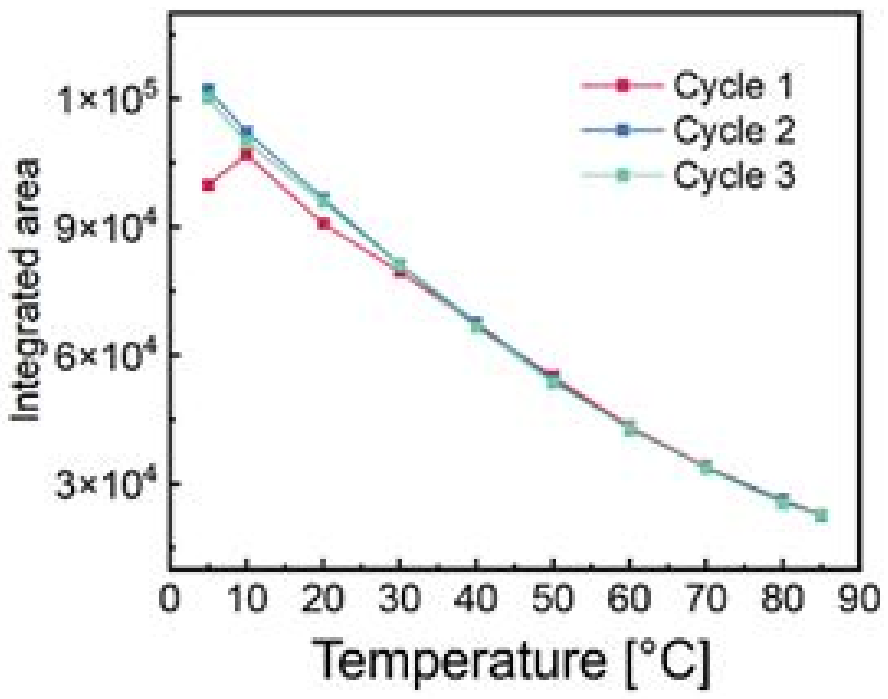

Figure S61. Initial version of Figure 5(b). Lifetime-based temperature sensing with **Ruphenpy@ARGSiO$_2$** in air-saturated: Integral PL decay curves obtained by integrating the area below the normalized PL decay curves.

Table S16. Lifetime-based temperature sensing with **Ruphenpy@ARGSiO$_2$** in air-saturated solution: Average decay time ⟨τ⟩ derived from a 4-exponential reconvolution fit and integral PL decay curves obtained by integrating the area below the normalized PL decay curves.

	Average decay time calculated from FAST 4-exponential reconvolution [μs]			Integrated area under the decay curve values		
	**Cycle 1**	**Cycle 2**	**Cycle 3**	**Cycle 1**	**Cycle 2**	**Cycle 3**
**5 °C**	554	562	571	99804	122185	119990
**10 °C**	523	501	522	106946	112193	109905
**20 °C**	371	404	399	90789	96740	95788
**30 °C**	308	298	301	79550	81209	80985
**40 °C**	229	226	225	67094	67631	66575
**50 °C**	171	165	161	55081	54371	53593
**60 °C**	144	125	127	43355	42686	42998
**70 °C**	96	96	88	33929	33767	33523
**80 °C**	69	79	66	26276	26128	25357
**85 °C**	54	54	55	22842	22504	22764

The value obtained from the integration below the decay curve measured at 5 °C during the first heating cycle (cycle 1) was excluded from the data shown in Figure 5 in the manuscript because it appeared to be an outlier, i.e., it was significantly lower than the values obtained for the other cycles, which were mutually consistent. The full dataset, including this value, is shown in Table S15.

References

(1) Wang, C.; Kitzmann, W. R.; Weigert, F.; Förster, C.; Wang, X.; Heinze, K.; Resch-Genger, U. Matrix Effects on Photoluminescence and Oxygen Sensitivity of a Molecular Ruby. *ChemPhotoChem* **2022**, *6* (6), e202100296. DOI: https://doi.org/10.1002/cptc.202100296.
(2) Otto, S.; Grabolle, M.; Förster, C.; Kreitner, C.; Resch-Genger, U.; Heinze, K. [Cr(ddpd)2]3+: A Molecular, Water-Soluble, Highly NIR-Emissive Ruby Analogue. *Angewandte Chemie International Edition* **2015**, *54* (39), 11572–11576. DOI: https://doi.org/10.1002/anie.201504894.
(3) Tyson, D. S.; Henbest, K. B.; Bialecki, J.; Castellano, F. N. Excited State Processes in Ruthenium(II)/Pyrenyl Complexes Displaying Extended Lifetimes. *The Journal of Physical Chemistry A* **2001**, *105* (35), 8154–8161. DOI: 10.1021/jp011770f.
(4) Sell, A. C.; Wetzel, J. C.; Schmitz, M.; Maijenburg, A. W.; Woltersdorf, G.; Naumann, R.; Kerzig, C. Water-soluble ruthenium complex-pyrene dyads with extended triplet lifetimes for efficient energy transfer applications. *Dalton Transactions* **2022**, *51* (28), 10799–10808.
(5) Stöber, W.; Fink, A.; Bohn, E. Controlled growth of monodisperse silica spheres in the micron size range. *Journal of colloid and interface science* **1968**, *26* (1), 62–69.
(6) Tavernaro, I.; Cavelius, C.; Peuschel, H.; Kraegeloh, A. Bright fluorescent silica-nanoparticle probes for high-resolution STED and confocal microscopy. *Beilstein Journal of Nanotechnology* **2017**, *8* (1), 1283–1296.
(7) Schmidt, S.; Tavernaro, I.; Cavelius, C.; Weber, E.; Kümper, A.; Schmitz, C.; Fleddermann, J.; Kraegeloh, A. Silica nanoparticles for intracellular protein delivery: A novel synthesis approach using green fluorescent protein. *Nanoscale research letters* **2017**, *12* (1), 545.
(8) Standardization, I. O. f. *Particle size analysis — Dynamic light scattering (DLS)*; 2025.
(9) Osipova, V.; Tavernaro, I.; Ge, L.; Kitzmann, W. R.; Heinze, K.; Reithofer, M. R.; Resch-Genger, U. Complete protection of NIR-luminescent molecular rubies from oxygen quenching in air by L-arginine-mediated silica nanoparticles. *Nano Research* **2025**, *18*.
(10) Statham, P. J. Limitations to accuracy in extracting characteristic line intensities from X-ray spectra. *Journal of research of the National Institute of Standards and Technology* **2002**, *107* (6), 531.
(11) Puccini, A.; Liu, N.; Hemmer, E. Praseodymium-doped nanoparticles: candidates for near-infrared-II double-and single-band nanothermometry. *ACS Materials Letters* **2024**, *6* (4), 1327–1337.
